# Sequential infection experiments for quantifying innate and adaptive immunity during influenza infection

**DOI:** 10.1371/journal.pcbi.1006568

**Published:** 2019-01-17

**Authors:** Ada W. C. Yan, Sophie G. Zaloumis, Julie A. Simpson, James M. McCaw

**Affiliations:** 1 School of Mathematics and Statistics, The University of Melbourne, Parkville, Victoria, Australia; 2 MRC Centre for Global Infectious Disease Analysis, Department of Infectious Disease Epidemiology, School of Public Health, Imperial College London, London, United Kingdom; 3 Centre for Epidemiology and Biostatistics, Melbourne School of Population and Global Health, The University of Melbourne, Parkville, Victoria, Australia; 4 Modelling and Simulation, Infection and Immunity Theme, Murdoch Childrens Research Institute, The Royal Children’s Hospital, Parkville, Victoria, Australia; University of Georgia, UNITED STATES

## Abstract

Laboratory models are often used to understand the interaction of related pathogens via host immunity. For example, recent experiments where ferrets were exposed to two influenza strains within a short period of time have shown how the effects of cross-immunity vary with the time between exposures and the specific strains used. On the other hand, studies of the workings of different arms of the immune response, and their relative importance, typically use experiments involving a single infection. However, inferring the relative importance of different immune components from this type of data is challenging. Using simulations and mathematical modelling, here we investigate whether the sequential infection experiment design can be used not only to determine immune components contributing to cross-protection, but also to gain insight into the immune response during a single infection. We show that virological data from sequential infection experiments can be used to accurately extract the timing and extent of cross-protection. Moreover, the broad immune components responsible for such cross-protection can be determined. Such data can also be used to infer the timing and strength of some immune components in controlling a primary infection, even in the absence of serological data. By contrast, single infection data cannot be used to reliably recover this information. Hence, sequential infection data enhances our understanding of the mechanisms underlying the control and resolution of infection, and generates new insight into how previous exposure influences the time course of a subsequent infection.

## Author summary

The resolution of an influenza infection requires different components of the immune response to work together. Despite recent advances in mathematical modelling, we do not well understand how much each broad immune component contributes to this process at a given time. In this study, we show that laboratory data on the amount of virus over the course of a single infection is insufficient for inferring the contribution of each broad immune component. However, if the animals are exposed to two different virus strains with only days separating exposures, then the timing and strength of protection provided by the first infection against the second provides crucial additional information. We show how mathematical models can be used to recover the timing and strength of each immune component, thus enhancing our understanding of how an infection is controlled, and how a previous exposure changes the time course of a subsequent infection.

## Introduction

The influenza virus infects epithelial cells in the respiratory tract, causing respiratory symptoms such as coughing and sneezing, and systemic symptoms such as fever. Three main components of the immune response—innate, humoral adaptive and cellular adaptive immunity—work together to control an infection. Experiments have revealed the contribution of each major immune component to resolution of an infection, by suppressing each immune component in turn [1–5]. However, current mathematical models do not agree on how each major immune component contributes quantitatively.

A study by Dobrovolny *et al*. [1] highlights these discrepancies. The study showed that eight existing viral dynamics models [6–13] made different qualitative predictions when different components of the immune response were removed. Each model failed to reproduce the effect of removing at least one of the three components discussed above. The discrepancies arose because many models were only fitted to viral load data from a single infection.

It has been shown that many models can fit the viral load for a single infection well, including models without a time-dependent immune response which are thought to be less biologically realistic [6]; however, if data for multiple initial conditions are available, the viral load may have more features to distinguish between competing models [14–16]. One way of altering the initial conditions is through a previous or ongoing infection. We previously conducted a series of experiments where ferrets were sequentially infected with two influenza strains [17, 18]. When a short time interval (1–14 days) separated exposures, a primary infection protected against a subsequent infection. This protection likely arose through cross-immunity, whereby the immune response stimulated by one strain also protects against infection with another.

While immune markers indicated the approximate timing of each arm of the immune response [19], the strength of cross-protection due to each component was difficult to measure experimentally. We hypothesised that mathematical models can be used to gain further insight from these types of experiments. Few existing models include interactions between influenza strains on short timescales; hence, we constructed viral dynamics models to reproduce the qualitative observations of these experiments [20, 21]. The models also reproduce observations from a range of experiments where immune components were suppressed [22].

Here, we use simulations to show that these mathematical models allow us to extract the timing and strength of cross-protection from sequential infection data. By attributing cross-protection to specific immune components, the models lead to new insight into how previous exposure influences the time course of a subsequent infection. Moreover, we find that compared to single infection experiments, sequential infection experiments provide richer information on host immunity, and thus are potentially a powerful tool to study immune-mediated control of a primary infection.

## Results

### Synthetic data

As a first step to compare the information made available by sequential infection versus single infection experiments, we generated synthetic datasets for each scenario. Mimicking the experimental procedure of Laurie *et al*. [17], we generated a sequential infection dataset where ferrets were exposed to two influenza strains, with intervals of 1, 3, 5, 7, 10 and 14 days between exposures; and a single infection dataset where ferrets were exposed once only. Details are given in the Materials and Methods section. Using synthetic data means that we know the ‘true’ contribution of each immune component in resolving a single infection, and the ‘true’ extent of cross-protection between infections.

**Fig 1:**
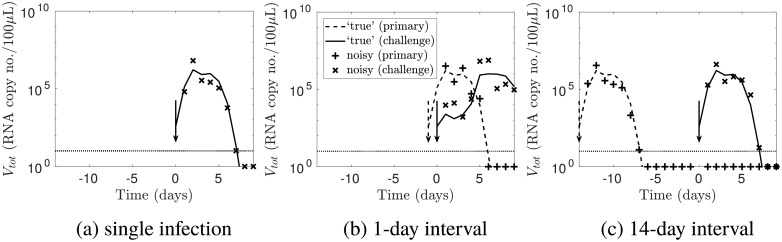
A subset of the synthetic data. (a) The line shows the simulated ‘true’ viral load for a single infection, with the arrow showing the time of exposure. The simulated viral load with noise is shown as crosses. The horizontal line indicates the observation threshold (10 RNA copy no./100*μ*L); observations below this threshold are plotted below this line. Values below the observation threshold were treated as censored. (b—c) For sequential infections with the labelled inter-exposure interval, the dashed and dotted lines show the simulated ‘true’ viral load for a primary and challenge infection respectively; the arrows show the times of the primary and challenge exposures. The simulated viral load with noise is shown as crosses.

Fig 1 shows a subset of the synthetic data. For a single infection, the viral load trajectory can be split into exponential growth, plateau and decay phases. For short inter-exposure intervals (1–5 days), infection with the challenge virus was delayed; for long inter-exposure intervals (7–14 days), infection with the challenge virus was unaffected. These features of the synthetic data match the qualitative results of Laurie *et al*. [17] for infection with influenza A followed by influenza B, or vice versa. The parameter values were chosen such that the delay was due to innate immunity. This choice was made because experimentally, innate immune markers such as type I interferon were observed to be elevated 1–5 days after a primary infection [19], and our previous mathematical model incorporating the innate immune response made predictions consistent with the observed temporary immunity [20]. The full set of synthetic data is provided in S1 Fig.

### Verification of the fitting procedure

In this section, we first verify that we could recover the simulated ‘true’ viral load by fitting our model to the data. In the next section, we will sample from the joint posterior distributions thus obtained to extract the contribution of each immune component.

**Fig 2:**
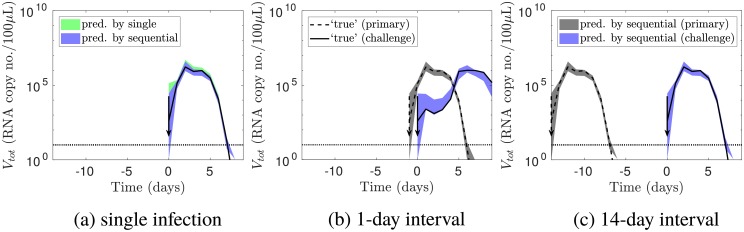
Verification that the fitting procedure recovered the viral load. (a) For a single infection, the blue and green areas are the 95% credible intervals for the viral load (in the absence of noise), as predicted by the models fitted to the sequential infection and single infection data respectively. (b—c) For sequential infections with the labelled inter-exposure interval, the grey and blue areas show the 95% credible intervals for the primary and challenge viral load respectively, predicted by the model fitted to sequential infection data. The other elements of the figure are identical to Fig 1: the dashed and dotted lines show the simulated ‘true’ viral load for a primary and challenge infection respectively; the arrows show the times of the primary and challenge exposures; and the horizontal line indicates the observation threshold.

Fig 2a shows that the simulated ‘true’ viral load was recovered accurately when fitting the model to either sequential infection or single infection data. The blue and green (overlapping) areas are 95% credible intervals predicted by the models fitted to the sequential infection and single infection data respectively. Both shaded areas included the simulated ‘true’ viral load, shown as the dotted line. This consistency indicates that the fitting procedure accurately recovered the viral load.

Fig 2b and 2c confirm that fitting to sequential infection data accurately recovered the viral load for different inter-exposure intervals. The grey and blue areas show the 95% credible intervals for the primary and challenge viral load respectively.

### Comparing the immunological information in each dataset

Next, we compared the behaviour of the fitted models to the behaviour of the ‘true’ parameters, to determine the information in each dataset on

- the effect of each immune component in controlling a single infection;
- cross-protection between strains; and
- each immune component’s contribution to cross-protection.

#### The effect of each immune component in controlling a single infection

**Fig 3:**
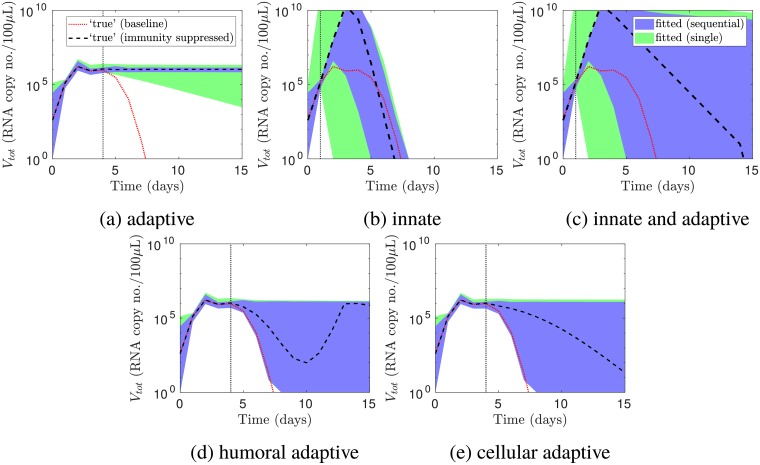
Predicting the viral load for a single infection when various immune components were absent. The vertical lines indicate, for the ‘true’ parameter values, the times at which the immune components labelled under each panel took effect. These times were determined by when the viral load for the baseline model (red dotted line) deviated from the viral load when the immune components were absent (black dashed line). These times were recovered using sequential infection data in all of the panels (95% prediction intervals for the viral load in blue), while the timing of adaptive immunity in (a) was recovered using single infection data (intervals in green). In addition, the viral load when adaptive immunity was suppressed was accurately predicted using sequential infection data (a). However, the viral load was not accurately predicted using either dataset in the remaining scenarios (b—e). Prediction intervals were constructed without measurement noise.

In Fig 3, we removed various immune components from the model. We then compared predictions of the viral load for a single infection by the models fitted to the two datasets.

First, we showed how the viral load trajectory for the ‘true’ parameters changed when adaptive immunity was suppressed. We defined the ‘baseline’ as the viral load when all immune components were present (red dotted lines in Fig 3, which are the same as the black lines in Figs 1a and 2a). Suppressing adaptive immunity prevented resolution of the infection (black dashed line in Fig 3a), which was consistent with findings of a previous experiment [5]. The viral load deviated from the baseline trajectory at 4 days post-exposure (vertical line), indicating that this was the time at which adaptive immunity took effect.

We then asked whether the models fitted to the two datasets predicted this change. Chronic infection in the absence of adaptive immunity was only predicted using sequential infection data (Fig 3a). Single infection data did not enable consistent prediction of this outcome, as indicated by the broadening prediction interval. However, both datasets enabled recovery of the time at which the viral loads in the presence and absence of adaptive immunity deviated (the vertical line in Fig 3a). Hence, the timing of adaptive immunity was accurately estimated using either dataset.

In Fig 3b–3e, we repeated this procedure, suppressing (b) innate immunity, (c) all immunity, (d) humoral adaptive immunity, or (e) cellular adaptive immunity. When innate immunity was suppressed, the peak viral load increased and the recovery time decreased. The increase in peak viral load was consistent with previous studies where innate immunity was suppressed [2, 23]. The decrease in recovery time was not observed in these studies, and may be an artefact of modelling adaptive immunity as independent of innate immunity. When both innate and adaptive immunity were suppressed, the peak viral load increased, and resolution of the infection was delayed. These changes were consistent with a previous experiment where innate immunity was suppressed [2].

Fig 3b shows that sequential infection data enabled accurate inference of when the viral loads in the presence and absence of innate immunity deviated, hence recovering the timing of innate immunity. By contrast, the model fitted to single infection data predicted that the viral loads could deviate much earlier. Neither model accurately predicted how the infection resolved in the absence of innate immunity; however, the prediction intervals for the model fitted to sequential infection data were tighter, and the peak viral load was consistently predicted to be higher than for the baseline model. Similarly, when both innate and adaptive immunity were absent, the model fitted to sequential infection data recovered the timing of overall immunity, but could not predict the viral load in the absence of immunity (Fig 3c).

Without innate immunity, the viral load peaks due to target cell depletion, and without any immune response, the infection resolves due to target cell depletion. The lack of predictive ability indicates that both datasets lack information on how target cells would hypothetically become depleted, and how this depletion would affect the viral load, in the absence of the immune response. One is thus cautioned against using parameter values from a model fitted to data in immunocompetent hosts to make predictions in situations where target cells may become severely depleted, such as if individuals are immunocompromised.

Fig 3d and 3e show that neither dataset enabled prediction of how the viral load changed when (d) the humoral adaptive immune response or (e) the cellular adaptive immune response was removed. This implies that sequential infection data (of the type reported in Laurie *et al*. [17]) cannot be used to distinguish the contributions of antibodies and cellular adaptive immunity to resolution of infection. In detail, the ‘true’ parameters predicted that when humoral adaptive immunity was disabled, the viral load rebounded instead of continuing to decrease (black dashed line in Fig 3d). When cellular adaptive immunity was disabled, resolution of the infection was delayed (black dashed line in Fig 3e). The fitted model’s predictions ranged from no delay to a chronic infection.

#### Cross-protection between strains

Given the above mixed results, we then tested whether sequential infection data accurately captured the timing and extent of cross-protection, by simulating the viral load for inter-exposure intervals other than those where data was provided. We selected new inter-exposure intervals of 2 and 20 days; the former lay between inter-exposure intervals included in the original data (1, 3, 5, 7, 10 and 14 days), while the latter lay outside this range. Then, using the models fitted to the original data (that is, not re-fitting to the new data), we predicted the challenge viral load for these new inter-exposure intervals. Because a primary infection could greatly affect a challenge infection, but not vice versa, we focused on the behaviour of the challenge infection.

**Fig 4:**
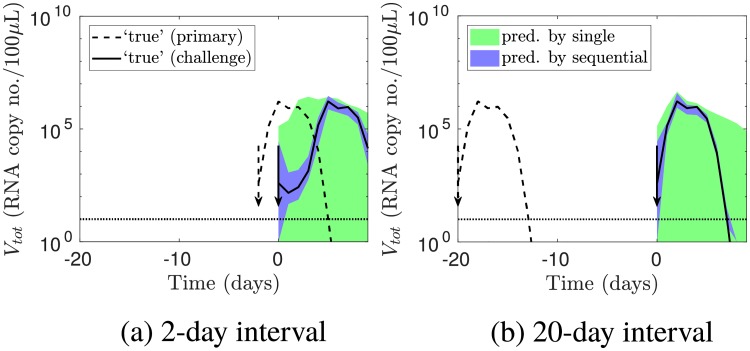
Predicting the outcomes of further sequential infection experiments. Sequential infection data, but not single infection data, enabled prediction of further sequential infection experiment outcomes. The lines show the simulated ‘true’ viral loads for inter-exposure intervals of (a) 2 and (b) 20 days. The shaded areas show the 95% prediction intervals for the challenge viral load.

Fig 4 shows that predictions by the model fitted to sequential infection data (blue areas) were accurate. By contrast, predictions using single infection data (green) did not agree well with the ‘true’ viral load. Note that to predict cross-protection using single infection data, we used the model assumptions that innate immunity was completely non-specific and antibodies were completely strain-specific, and considered the optimistic scenario where we had independent, perfect information about the proportion of cellular adaptive immunity that was cross-reactive (details in the Materials and methods section). Even then, single infection data did not accurately capture cross-protection.

#### Each immune component’s contribution to cross-protection

Having shown that the sequential infection data captures the timing and extent of cross-protection between strains, we then asked whether such cross-protection could be attributed to the ‘correct’ mechanisms (the same mechanisms as given by the ‘true’ parameters). These mechanisms are

- target cell depletion due to the infection and subsequent death of cells;
- innate immunity; and
- cellular adaptive immunity.

In our model, antibodies are strain-specific and thus do not contribute to cross-protection.

**Fig 5:**
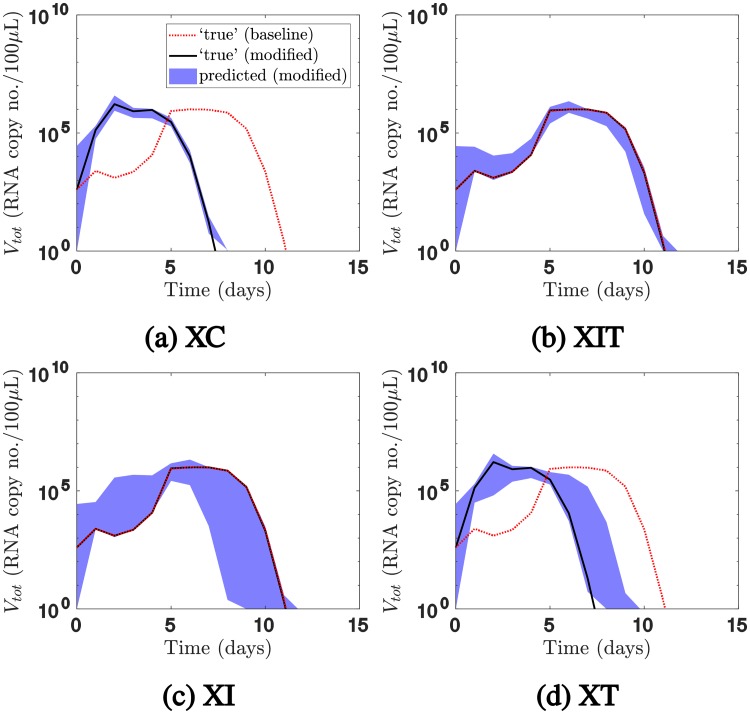
Predictions of the challenge viral load for a one-day inter-exposure interval when the mechanisms mediating cross-protection were restricted. The black solid lines show the challenge viral load for the ‘true’ parameter values when the mechanisms mediating cross-protection were restricted using (a) model XC, (b) model XIT, (c) model XI, or (d) model XT. The red dotted lines show the viral load for the baseline model. Comparing the two sets of lines revealed that innate immunity mediated cross-protection, whereas cellular adaptive immunity and target cell depletion did little to mediate cross-protection. The model fitted to sequential infection data accurately predicted the challenge outcomes for models XC and XIT, but not model XI or model XT (95% prediction intervals shown). It thus correctly attributed cross-protection to target cell depletion and/or innate immunity, but could not definitively distinguish between the two. For clarity, the viral load for the primary infection is not presented in this figure.

Before analysing the behaviour of the fitted models, we quantified how each immune component contributed to cross-protection for the ‘true’ parameters. In Fig 5, for a one-day inter-exposure interval, we plotted in red the challenge viral load for the baseline model (the original model fitted to the data, where all three of the above immune components can mediate cross-protection). We observed that the challenge infection was delayed relative to a primary infection. We then modified the baseline model such that only a subset of immune components mediates cross-protection, as detailed in the Materials and Methods section. We used the modified model to predict the viral load (in black), and compared it with the baseline viral load. (The blue areas will be discussed shortly).

For example, in Fig 5a, we modified the baseline model such that only cellular adaptive immunity, and not target cell depletion or innate immunity, can mediate cross-protection. We denoted this modified model ‘model XC’. Unlike the baseline model (red dotted line), the challenge viral load for model XC was not delayed (black solid line); in fact, it closely resembled that for a single infection. Comparing the two simulations led to the conclusion that cellular adaptive immunity did not play a major part in cross-protection.

We then modified the baseline model such that both target cell depletion and innate immunity can mediate cross-protection, but cellular adaptive immunity cannot do so. We denoted this model ‘model XIT’. The challenge viral loads according to model XIT and the baseline model were very similar (overlapping lines in Fig 5b). Hence, for the ‘true’ parameters, cross-protection was mediated by innate immunity and/or target cell depletion.

To distinguish between these two mechanisms, we constructed model XI, where only innate immunity, and not target cell depletion or cellular adaptive immunity, can mediate cross-protection. Once again, the challenge viral load was very similar to the baseline model (overlapping lines in Fig 5c). We also constructed model XT, where only target cell depletion, and not innate immunity or cellular adaptive immunity, can mediate cross-protection. The challenge viral load for model XT was not delayed, and resembled that for a single infection (Fig 5d). We concluded that the cross-protection was largely mediated by innate immunity.

Having demonstrated the utility of the modified models, we returned to the original question of whether sequential infection data could be used to distinguish between mechanisms for cross-protection. We sampled parameter sets from the joint posterior distributions obtained by fitting the baseline model to sequential infection data, and used them as inputs for models XC, XIT, XI and XT respectively, to generate the blue areas in Fig 5. If the fitted parameters and the ‘true’ parameters predict the same infection outcomes under the modified models, then the fitted model attributes cross-protection to the ‘correct’ mechanisms.

Models XC and XIT made the same predictions using the fitted parameters (shaded area) and the ‘true’ parameters (black line), so sequential infection data enabled us to accurately attribute cross-protection to target cell depletion and/or innate immunity, rather than cellular adaptive immunity (Fig 5a and 5b). On the other hand, the fitted parameters did not consistently predict the challenge outcome for models XI and XT (Fig 5c and 5d). Hence, we could not use sequential infection data to consistently rule out the possibility that cross-protection occurred due to both target cell depletion and innate immunity. However, only a very small proportion of trajectories sampled from the joint posterior distribution incorrectly attributed the delay to both target cell depletion and innate immunity (S2 Fig). Moreover, the fitted model was able to rule out the possibility that target cell depletion alone was responsible for cross-protection, as the 95% credible interval for model XT does not include the 95% credible interval for the baseline viral load.

Similarly, we were unable to disentangle different mechanisms of innate immunity from the sequential infection data alone (S3 Fig and S1 File).

For infection with heterologous influenza A strains, rather than the influenza A and B strains discussed thus far, we hypothesise that innate and cellular adaptive immunity contribute to cross-protection at different inter-exposure intervals [24]. S2 File presents the same analysis for this scenario, where we were able to unravel these different contributions using sequential infection data.

Our findings are robust to the ‘true’ parameters chosen, given that the parameters capture the qualitative observations by Laurie *et al*. [17]. S5 File presents the same analysis for a different set of ‘true’ parameters where the degree of cross-reactivity in the cellular adaptive immune response is low. For the most part, the same qualitative results were obtained: sequential infection data enabled inference of the timing of innate and adaptive immunity, prediction of the viral load for further experiments with different inter-exposure intervals, and prediction of the viral load for models XC and XIT. The main difference was that the model fitted to sequential infection data was only able to capture the timing of adaptive immunity, and not how the infection would resolve if adaptive immunity were removed. A possible explanation for the different result is that for the parameters in the main text, the viral load showed a clear plateau while innate but not adaptive immunity was active, enabling the fitted model to predict that the viral load would stay at that plateau in the absence of adaptive immunity. By contrast, the viral load for the different set of parameters in the supplementary material did not show a clear plateau during the innate immunity phase, possibly reducing the fitted model’s ability to infer the viral load in the absence of adaptive immunity. Another minor difference was that the immune components whose timing could be inferred using single infection data were different from in the main text, although the overall finding—that sequential infection data enabled inference of the timing of more immune components—was the same.

For a given set of ‘true’ parameters, repeating the entire study with different simulated noisy datasets did not change our findings (S6 File).

Because our model does not capture every biological detail of the experimental system, we also tested whether our findings were robust to model misspecification. We generated data using a model from a study by Zarnitsyna *et al*. [25], modified to include a variable degree of cross-reactivity between strains. We then fitted the model in the present study to the generated data. Even though the data was generated using a different model, we were still able to infer the timing of innate and adaptive immunity, predict the outcome of further experiments with different inter-exposure intervals, and distinguish the contributions of different components of the immune response to cross-protection. This analysis is presented in S7 File.

In summary, the synthetic sequential infection data enabled accurate inference of the contribution of cellular adaptive immunity to cross-protection, as well as the combined contributions of target cell depletion and innate immunity. However, using this data alone, we could not conclusively distinguish the contributions of innate immunity and target cell depletion to cross-protection, or distinguish the contributions of different innate immune mechanisms.

## Discussion

### Advantages of sequential infection experiments

In this study, we have simulated experiments which investigate the interaction of influenza strains through sequential infections, then explored how mathematical models could be applied to the data to gain insight into immune mechanisms. Our analysis has shown that the sequential infection study design, compared to the single infection study design, provides richer information for inferring the timing and strength of each immune component.

We have identified three main advantages of sequential infection data. The first advantage is in inferring how each immune component helps to resolve a single infection. We found that the synthetic sequential infection data captures the timing of innate and adaptive immunity during a single infection, and thus enables accurate prediction of the outcomes of some *in silico* experiments where immune components were removed. In contrast, we could not consistently infer the timing and strength of innate immunity from single infection data. Moreover, single infection data contains information only on the timing of adaptive immunity, but not the effects of suppressing adaptive immunity.

The second advantage is that sequential infection data contains more information on the effects of cross-protection. We were able to use the model fitted to the sequential infection data to precisely predict outcomes of further such experiments using the same strains but different inter-exposure intervals. Using the model fitted to the single infection data greatly reduced predictive power.

The third advantage is in inferring the contribution of each immune component to this cross-protection. For the dataset in the main text, we were able to infer that cellular adaptive immunity played little role in cross-protection, and that innate immunity and/or target cell depletion led to the observed cross-protection. We also showed that target cell depletion alone could not explain this cross-protection.

Collectively, the above findings strongly suggest that analysing real sequential infection data using mathematical models will help infer the timing and strength of host immunity, which are difficult to measure directly in laboratory experiments. Such mathematical models will not only have the ability to explain observed experimental outcomes, but the ability to predict outcomes of new experiments, which can then be tested in the laboratory. These findings are particularly important as sequential infection experiments are increasingly being used to study the role of the immune response during infection with influenza and other respiratory pathogens [18, 26].

### Limitations of sequential infection experiments

This study has highlighted some limitations of quantifying the immune response using virological data from sequential infection experiments alone.

Firstly, using the synthetic sequential infection data, we could not discriminate between the effects of cellular and humoral adaptive immunity in controlling a primary infection. If the effects of cellular and humoral adaptive immunity need to be distinguished, such as to predict the effects of vaccines that boost these components separately, quantities other than the viral load may need to be measured.

Secondly, we could not definitively rule out the possibility that target cell depletion contributed significantly to cross-protection. We were also unable to distinguish the roles of different innate immune mechanisms in cross-protection. Some modelling applications may require the strengths of different innate immune mechanisms to be known separately. An example of such an application is modelling the effect of treatments that modulate the innate immune response, such as the toll-like receptor-2 agonist Pam2Cys which has been shown to stimulate innate immune signals and reduce influenza-associated mortality and morbidity in animal studies [27].

In this simulation-based study, we were able to compare inferred quantities to a ‘ground truth’, to understand which quantities were inferred accurately, and which inferences may need to be treated with caution. For example, in S4 File, we show that the marginal posterior distributions for some parameters exhibit bias, such as that for the basic reproduction number *R*_0_. These apparent biases reflect correlations in the joint posterior distribution, and would be difficult to identify without a simulation-based study. This approach is thus crucial for sound interpretation of future studies fitting models to experimental data.

In addition to total viral load data, the study by Laurie *et al*. [17] also included infectious viral load measurements for single infection ferrets, and serological responses and cytokine levels at limited time points. Inclusion of this data could help to address the above limitations; the utility of this additional data can be assessed by further simulation-based studies.

New experiments could also be conducted to improve parameter estimates, leading to more accurate inference of the timing and strength of immune components. Previous studies have measured viral decay rates *in vitro* and incorporated these estimates into model fitting [28, 29]. *In vitro* studies can also directly measure the time course of those immune mechanisms which are active *in vitro* [30].

### Future work and concluding summary

Now that we have shown how mathematical models can increase the utility of sequential infection experiments, fitting the model to the experimental data by Laurie *et al*. [17] is a priority. A simulation-estimation study alone cannot validate the mathematical model used, or infer the effects of host immunity against the pathogens in the experiments. However, this simulation-based study ensures that results will be interpreted appropriately when the models are fitted to data.

We have demonstrated that compared to single infection experiments, the sequential infection study design helps us to better understand cross-protection on short timescales. Further, data from sequential infection experiments helps to discriminate between existing models for a primary infection, leading to an improved understanding of the control and resolution of infection.

## Materials and methods

### The model

#### Viral dynamics

The viral dynamics model is based on a model we previously published [24]. It incorporates three major components of the immune response—innate, humoral adaptive and cellular adaptive.

**Fig 6:**
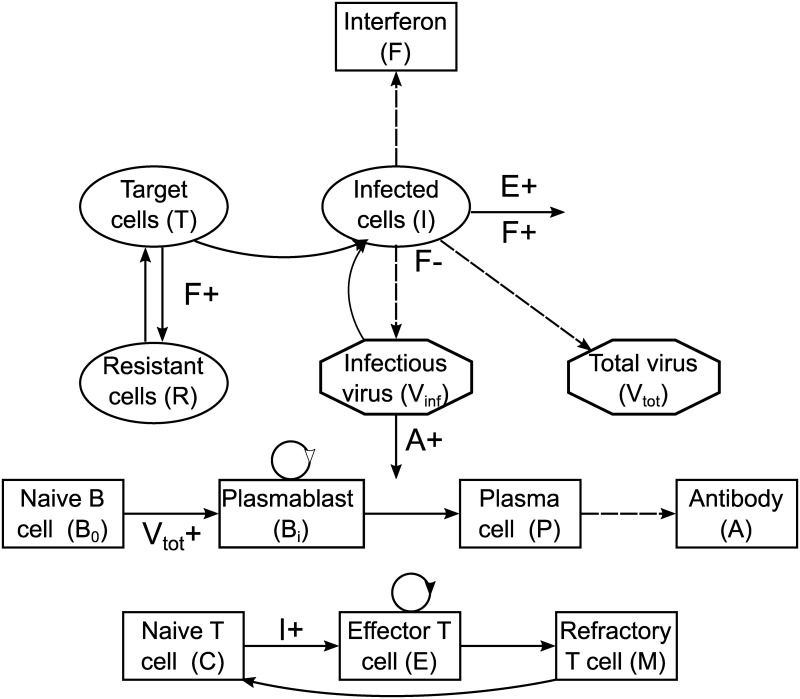
The within-host influenza model for a single strain. (Top) Viral dynamics and innate immune response; (middle) humoral adaptive immune response; (bottom) cellular adaptive immune response. Solid arrows indicate transitions between compartments or death (shown only for immune-enhanced death processes); dashed arrows indicate production; plus signs indicate an increased transition rate due to the indicated compartment.

Fig 6 shows a compartmental diagram of the model for a single strain. The system is described by a coupled set of ordinary differential equations (Eqs 1–4).

$$\begin{array}{c} \begin{array}{cc} \frac{dT}{dt} & =g\left(T+R\right)(1-\frac{T+R+I}{T_{0}})-\beta V_{inf}T+\rho R-\phi FT,\\ \frac{dI}{dt} & =\beta V_{inf}T-(\delta_{I}+\kappa_{F}F+\kappa_{E}E)I,\\ \frac{dV_{inf}}{dt} & =\frac{p_{Vinf}}{1+sF}I-\left(\delta_{Vinf}+\kappa_{A}A+\beta T\right)V_{inf},\\ \frac{dV_{tot}}{dt} & =\frac{p_{Vinf}p_{Vratio}\alpha }{1+sF}I-\delta_{Vtot}V_{tot}-\alpha \beta TV_{inf}. \end{array} \end{array}$$ (1)

Eq 1 describes the dynamics of target cells (*T*), infected cells (*I*) and virions (*V*_*inf*_ and *V*_*tot*_ for infectious and total virions respectively). Virions (*V*_*inf*_) bind to target cells (*T*) to infect them; infected cells (*I*) produce virions; and infected cells and virions both decay at a constant rate. Target cells also regrow, with an imposed carrying capacity. Immunity is mediated by the compartments *R* (resistant cells), *F* (type I interferon), *A* (antibodies) and *E* (effector CD8^+^ T cells), the dynamics of which will be described shortly. Descriptions of model parameters are given in S1–S4 Tables, and in our previous publication [24].

The compartment *V*_*inf*_ refers to the number of infectious virions in the host; however, an infected cell produces both infectious and non-infectious virions, the latter of which arise due to defects introduced during the viral replication process [31, 32]. Moreover, in the experiments conducted by Laurie *et al*. [17], the total viral load, rather than the number of infectious virions, was measured. An additional complication is that the concentration of total nasal wash, rather than the absolute number of virions, was measured. Hence, we include an equation for the total virion concentration *V*_*tot*_.

Innate immunity is mediated through type I interferon (*F*), the production of which is stimulated by infected cells. Three effects of type I interferon are modelled through Eqs 1 and 2:

- rendering target cells temporarily resistant to infection (*T* → *R*);
- decreasing the production rate of virions from infected cells; and
- increasing the decay rate of infected cells.

$$\begin{array}{c} \begin{array}{cc} \frac{dR}{dt} & =\phi FT-\rho R,\\ \frac{dF}{dt} & =I-\delta_{F}F. \end{array} \end{array}$$ (2)

The humoral adaptive immune response is mediated by antibodies (*A*), which bind to virions and neutralise them, rendering them non-infectious. Naive B cells (*B*_0_) are stimulated by virus to proliferate and differentiate into plasma cells (*P*), which produce antibodies. Eq 3 describes these processes.

$$\begin{array}{c} \begin{array}{cc} \frac{dB_{0}}{dt} & =-\frac{V_{tot}}{k_{B}+V_{tot}}\beta_{B}B_{0},\\ \frac{dB_{1}}{dt} & =\frac{V_{tot}}{k_{B}+V_{tot}}\beta_{B}B_{0}-(\frac{n_{B}}{\tau_{B}}+\delta_{B})B_{1},\\ \frac{dB_{i}}{dt} & =\frac{2n_{B}B_{i-1}}{\tau_{B}}-(\frac{n_{B}}{\tau_{B}}+\delta_{B})B_{i},\;i=2,\ldots ,n_{B},\\ \frac{dP}{dt} & =\frac{2n_{B}B_{n_{B}}}{\tau_{B}}-\delta_{B}P,\\ \frac{dA}{dt} & =P-\delta_{A}A. \end{array} \end{array}$$ (3)

The cellular adaptive immune response is mediated by effector CD8^+^ T cells (*E*). Infected cells stimulate the differentiation of effector CD8^+^ T cells from their naive counterparts (*C*); effector CD8^+^ T cells then increase the death rate of infected cells. Some effector CD8^+^ T cells remain after a primary infection as memory CD8^+^ T cells. After a refractory period (represented by the *M* stage), they are modelled as having the same dynamics as naive cells, and can be re-stimulated to become effector CD8^+^ T cells upon challenge. Eq 4 describes these processes.

$$\begin{array}{c} \begin{array}{cc} \frac{dC}{dt} & =\frac{M}{\tau_{M}}-\frac{I/k_{C}}{1+I/k_{C}}\beta_{C}C,\\ \frac{dE_{1}}{dt} & =\frac{I/k_{C}}{1+I/k_{C}}\beta_{C}C-(\frac{n_{E}}{\tau_{E}}+\delta_{E})E_{1},\\ \frac{dE_{i}}{dt} & =\frac{2n_{E}E_{i-1}}{\tau_{E}}-(\frac{n_{E}}{\tau_{E}}+\delta_{E})E_{i},\;i=2,\ldots ,n_{E}-1,\\ \frac{dE_{n_{E}}}{dt} & =\frac{2n_{E}E_{n_{E}-1}}{\tau_{E}}-\delta_{E}E_{n_{E}},\\ E & =\sum_{i=1}^{n_{E}}E_{i},\\ \frac{dM}{dt} & =\epsilon \delta_{E}E_{n_{E}}-\delta_{E}M-\frac{M}{\tau_{M}}. \end{array} \end{array}$$ (4)

When more than one strain co-infects the host, the strains interact in three ways:

- competition for target cells, which become depleted due to the infection and subsequent death of cells;
- innate immunity, which acts across all strains; and
- cellular adaptive immunity, which can be partly cross-reactive.

Activation of each of these mechanisms by the primary virus lowers the effective reproduction number of the challenge strain, but to different extents depending on parameter values. Note that because the model includes target cell regrowth, infection with the challenge virus can become established despite target cell depletion due to the death of infected cells. Each naive CD8^+^ T cell pool can be stimulated by one or more virus strains, depending on model parameters; cross-reactivity arises when a T cell pool can be stimulated by more than one virus strain. The clearance of infected cells by effector CD8^+^ T cells is similarly strain-specific. The antibody response is modelled as completely strain-specific, with no cross-reactivity between strains. It is thus unnecessary to include long-term humoral adaptive immunity. Extension of the model to include the potential effects of antibody-mediated cross-protection (as reviewed by [33]) is the subject of future work.

S4 Fig illustrates the model for two strains and three T cell pools; the equations are given in S1 File. Three T cell pools is a parsimonious choice, to allow for one pool to be cross-reactive between strains and two pools to be strain-specific, one for each strain.

#### Observation model

Observations were simulated from the ‘true’ viral load by adding lognormal noise and imposing a detection threshold. Mathematically, the measured viral load *y*_*qfk*_ for each virus *q* = 1, 2, …,*Q*, ferret *f* = 1, 2, …, *F* and measuring time point *t*_*qfk*_ where *k* = 1, 2, …, *K*_*qf*_ is given by

$$\begin{array}{c} \begin{array}{cc} y_{qfk} & =\{\begin{array}{c} V_{totq}\left(t_{qfk},u_{f},\beta \right)10^{e_{qfk}}\;\text{when}\;V_{totq}\left(t_{qfk},u_{f},\beta \right)10^{e_{qfk}}\geq \Theta \\ 0\;\text{otherwise} \end{array}\\ \text{where}\;e_{qfk} & \overset{i.i.d.}{∼}N\left(0,\sigma \right). \end{array} \end{array}$$ (5)

***β*** is a vector of parameter values, *u*_*f*_ is the inter-exposure interval for ferret *f*, *e*_*qfk*_ is the measurement error, and Θ is the detection threshold. *V*_*totq*_(*t*_*qfk*_, *u*_*f*_, ***β***) is the solution to the two-strain version of Eqs 1–4 for the *V*_*totq*_ compartment at time *t*_*qfk*_ for the given parameter values and inter-exposure intervals. Θ takes the value 10 RNA copies/100*μ*L in the experiments by Laurie *et al*. [17]. A measured viral load of 0 denotes that the viral load is below the detection threshold.

Therefore the likelihood of the model given the data is

$$\begin{array}{c} \begin{array}{cc} P(y|\theta ) & =\prod_{q=1}^{Q}\prod_{f=1}^{F}\prod_{k=1}^{K_{qf}}P\left(y_{qfk}|\theta \right)\;\text{where}\\ P(y_{qfk}|\theta ) & =\{\begin{array}{cc} \frac{1}{\sqrt{2\sigma^{2}\pi }}\text{exp}\{-\frac{[{\text{log}}_{10}y_{qfk}-{\text{log}}_{10}V_{totq}\left(t_{qfk},u_{f},\beta \right){]}^{2}}{2\sigma^{2}}\} & \text{if}\;y_{qfk}\geq \Theta ,\\ \int_{0}^{\Theta }\frac{1}{\sqrt{2\sigma^{2}\pi }}\text{exp}\{-\frac{[{\text{log}}_{10}x-{\text{log}}_{10}V_{totq}\left(t_{qfk},u_{f},\beta \right){]}^{2}}{2\sigma^{2}}\}dx & \text{if}\;y_{qfk}=0,\\ 0 & \text{otherwise}. \end{array} \end{array} \end{array}$$ (6)

In the second line of Eq 6, the likelihood when the data is below the detection threshold is obtained by integrating the probability density function from 0 to the detection threshold, i.e. treating the data below the threshold as censored [34]. The vector ***θ*** contains the parameters ***β***, the inter-exposure intervals *u*_*f*_, the time points *t*_*qfk*_, and the standard deviation *σ* of the measurement error.

### Simulated experiments

The model and the chosen ‘true’ parameters were used to generate synthetic data akin to that in Laurie *et al*. [17]. For six ferrets, intervals of 1, 3, 5, 7, 10 and 14 days separated exposures to two influenza strains. In addition, thirteen ferrets were exposed to a single influenza strain only. The sequential infection dataset consists of the viral load for the six sequential infection ferrets and one single infection ferret; the single infection dataset consists of the viral load for the thirteen single infection ferrets. The number of single infection ferrets was chosen such that the number of exposures to influenza virus is the same in each dataset, and so the number of data points is roughly the same.

### Selection of model parameters to generate synthetic data

The ‘true’ parameter values chosen to generate the synthetic data are given in S1–S4 Tables. The parameters were assumed to be identical between the two strains, except for the parameters governing cross-reactivity in the cellular adaptive immune response. In addition to the criteria discussed in the Results section, the parameters were chosen to reproduce qualitative behaviour for a single infection when immune components are suppressed:

- when the innate adaptive immune response is absent (*F* → 0), the peak viral load increases [2];
- when the humoral adaptive immune response is absent (*A* → 0), the viral load rebounds [3];
- when the cellular adaptive immune response is absent (*E* → 0), resolution of the infection is delayed [4]; and
- when both arms of the adaptive immune response are absent (*A*, *E* → 0), chronic infection ensues [5].

For an extensive evaluation of a very similar model’s behaviour under these types of conditions (for single infection events), see [22].

In addition to parameter values, initial values were required when simulating infections. For a single infection, the initial values for all compartments in Eqs 1–4 except *T*, *V*_*inf*_, *V*_*tot*_, *C* and *B*_0_ were zero. The initial values of *C* and *B*_0_ (naive T and B cells respectively) were normalised to 1. The initial values of *T* and *V*_*inf*_ (the number of target cells and the concentration of infectious virions respectively) were estimated parameters. The initial concentration of total virus was then *V*_*tot*_(0) = *γαV*_*inf*_(0), where *γ* and *α* were conversion parameters described in S1 Table. For sequential infections, the conditions at the time of the primary exposure were as above; the system was integrated until the time of the challenge exposure, at which *V*_*inf*,2_(0) infectious virions for the challenge strain was added to the system, and the total concentration of the challenge strain was set to *V*_*tot*,2_(0).

### Model fitting

#### Parameters to be estimated

All model parameters were estimated, except for the following parameters which were either fixed or a function of other estimated parameters. We fixed two parameters—the number of plasmablast division cycles (*n*_*B*_) and the number of effector CD8^+^ T cell division cycles (*n*_*E*_)—to be 5 [35, 36] and 20 [37] respectively. In addition, when fitting the model to single infection data, we considered the optimistic scenario where we had independent, perfect information about the proportion of cellular adaptive immunity during a primary infection that was cross-reactive with the challenge strain. As one T cell pool was cross-reactive between strains and two pools were strain-specific, this amounted to fixing the proportion of cellular adaptive immunity attributed to each T cell pool. We did so by fixing the numbers of infected cells for half-maximal stimulation of naive CD8^+^ T cells *k*_*Cj*1_ to their ‘true’ values for each T cell pool *j*. Then when we extended the model to two strains, we set *k*_*Cjq*_ to these same ‘true’ values. We then calculated the clearance rates of infected cells by effector CD8^+^ T cells *κ*_*Ejq*_ by taking the fitted value of *κ*_*E*11_, and applying the formula *κ*_*Ejq*_ = *κ*_*E*11_ *k*_*C*11_/*k*_*Cjq*_ (see S1 File).

Instead of fitting the infectivity (*β*) and the production rate of infectious virions from an infected cell (*p*_*Vinf*_), we fitted the basic reproduction number *R*_0_ (Eq 7) and the initial viral load growth rate *r* (Eq 9), as we hypothesised that these were more intimately linked to features of the viral load curve. Practically speaking, we proposed a new value for *R*_0_ (or *r*), calculated the corresponding values of *β* and *p*_*Vinf*_, solved the model equations, calculated the likelihood of the data given the parameters, and accepted or rejected the new value for *R*_0_ (or *r*).

The basic reproduction number *R*_0_ is the mean number of secondary infected cells due to (the virions produced by) a single infected cell. The expression for *R*_0_ is

$$R_{0}=\frac{\beta T_{0}p_{Vinf}}{\left(\delta_{Vinf}+\beta T_{0}\right)\delta_{I}},$$ (7)

and is the same as that for a model without a time-dependent immune response [38].

The viral load during early infection can be approximated by

$$V=V_{0}\text{exp}\left(rt\right).$$ (8)

Arenas *et al*. [39] showed using a simulation-estimation study that this parameter was well estimated even when only viral load data was available.

The expression for *r*, derived by linearising Eq 1 around the disease-free equilibrium [40], is

$$r=-\frac{\delta_{Vinf}+\beta T_{0}+\delta_{I}}{2}+\frac{\sqrt{{\left(\delta_{I}-\delta_{Vinf}-\beta T_{0}\right)}^{2}+4\beta T_{0}p_{Vinf}}}{2}.$$ (9)

#### Prior distributions

We began with a uniform distribution in parameter space whose bounds along each dimension are given in S1–S4 Tables. Note that parameter estimation was performed in a parameter space where all parameters except for the standard deviation of the measurement error *σ* were log transformed. Then, we excluded regions of parameter space where the parameters log_10_ *β* and log_10_ *p*_*Vinf*_, which were not directly estimated but were instead recovered from Eqs 7 and 9, were outside the bounds given in S1 Table.

The priors were deliberately chosen to be wide because previous parameter estimates came from a range of experimental systems, and parameters with similar physical definitions could vary in value depending on the model used. The bounds for viral replication parameters were based on those by Petrie *et al*. [41] where the equivalent parameters exist. Otherwise, where multiple estimates existed in the literature (as cited in the tables), the bounds were chosen to encompass all of them. Where we could only find a single estimate, bounds spanning at least an order of magnitude were chosen (unless the parameter is a pure rate parameter, as discussed shortly). Where no estimate was found, we assigned very wide bounds spanning much more than one order of magnitude. In general, the bounds for pure rate parameters (those with units day^−1^ only) were chosen to be narrower as their order of magnitude was known, whereas bounds for parameters such as *R*_0_ were much wider.

Furthermore, for computational efficiency, some minimal constraints on the behaviour of the viral load and timing of various immune components were incorporated into the prior distribution. These constraints were imposed because parameter sets that generate ‘unreasonable’ viral load trajectories for a single infection caused large delays in numerical integration of the two-strain differential equations. The inclusion criteria were that for a single infection,

- the total viral load rises by at least one order of magnitude during infection;
- the total viral load peaks 0–7 days post-exposure;
- the humoral adaptive immune response is not active too early (five days post-exposure, the neutralisation rate of virus by antibodies, *κ*_*A*_*A*, does not exceed 10^3^ day^−1^); and
- the cellular adaptive immune response is not active too early (five days post-exposure, the clearance rate of infected cells by effector CD8^+^ T cells, $\sum_{j=1}^{J}\kappa_{Ej1}E_{j}$, does not exceed 10^3^ day^−1^).

If the viral load trajectory (in the absence of measurement noise) predicted by a parameter set does not fulfil all of these conditions, the value of the prior distribution is zero at that point in parameter space.

#### Model fitting algorithm

We fitted the model using the Metropolis algorithm [42, 43] embedded within a Gibbs sampler structure [44], implemented in Octave 3.8.2 [45]. To evaluate the likelihood, Eqs 1–4 were solved using the CVODE solvers developed by [46], implemented in MATLAB [47]. Of the available solvers, a backward differentiation formula method in variable order, variable step, fixed leading coefficient form was chosen. Extinction was enforced by defining an infection to have resolved if both the number of infected cells and virions was below 0.1.

To assess convergence, three chains were run in parallel using different starting parameter values ***θ***_0_ drawn from the prior distribution. The procedure for determining the number of iterations for which to run the chains is detailed in S1 Text. For efficient mixing, the proposal distributions were tuned such that the proportion of accepted proposals was not too low or too high, as detailed in S1 Text. For each of the three chains, parallel tempering (as developed by [48] and reviewed by [49]) was implemented to improve exploration of parameter space. The number of iterations before testing whether to swap chains in the parallel tempering process was set to 10. During the calibration period for the proposal distributions, the temperatures were also calibrated [50], as detailed in S1 Text. Once convergence was reached, the effective sample size was calculated for each chain (using the iterations that were kept following the burn-in process) using the effectiveSize function in the coda [51] package in R [52]. Convergence diagnostics for the chains are shown in S3 File.

The marginal posterior distributions in this study are plotted in S4 File using all samples from the chains (after burn-in), without thinning. When using the joint posterior distribution to make predictions, we used 10^4^ parameter sets corresponding to uniformly spaced iterations in each of the chains.

Results were visualised using MATLAB R2015b [53]. Code to reproduce all figures is provided at https://bitbucket.org/ada_yan/sim_est_immunity.

### Model predictions

First, to determine whether the fitted model captured the timing and strength of each immune component during a primary infection, we used parameter sets from the joint posterior distribution to simulate the viral load during a single infection, using a modified model where either

- adaptive immunity is suppressed;
- innate immunity is suppressed;
- innate and adaptive immunity are suppressed;
- cellular adaptive immunity is suppressed; or
- humoral adaptive immunity is suppressed.

95% prediction intervals were constructed using these simulations.

Second, to determine whether the fitted model captured cross-protection between strains, we used parameter sets from the joint posterior distribution to simulate different inter-exposure intervals.

Third, to determine whether the fitted model captured the contribution of each immune component to cross-protection between strains, we used parameter sets from the joint posterior distribution to simulate the viral load during sequential infection, using a modified model where either

- cross-protection is only mediated by cellular adaptive immunity, and not target cell depletion or innate immunity (model XC);
- cross-protection is mediated by innate immunity, but not target cell depletion or cellular adaptive immunity (model XI);
- cross-protection is mediated by target cell depletion, but not innate immunity or cellular adaptive immunity (model XT); or
- cross-protection is mediated by target cell depletion and/or innate immunity, but not cellular adaptive immunity (model XIT).

Details of models XC, XI, XT and XIT are provided in S1 File.

Table 1 summarises the model modifications in this section.

**Table 1:** Summary of model modifications for predictions.

Model	Target cells	Interferon	Antibodies	T cells
Baseline	shared	shared	separate	partly shared
No adaptive immunity	shared	shared	none	none
No innate immunity	shared	none	separate	partly shared
No immunity	shared	none	none	none
No cellular adaptive immunity	shared	shared	separate	none
No humoral adaptive immunity	shared	shared	none	partly shared
XC	separate	separate	separate	partly shared
XI	separate	shared	separate	separate
XT	shared	separate	separate	separate
XIT	shared	shared	separate	separate

‘Shared’ denotes that the compartment in the table header interacts with all virus strains. For example, if interferon are ‘shared’, all virus strains induce production of the same interferon, and interferon’s antiviral effects apply to all strains. ‘Separate’ denotes that the compartment interacts with one virus strain only. For example, if interferon are ‘separate’, each virus strain induces the production of a separate pool of interferon, and the antiviral effects of each pool of interferon apply only to that strain. T cells being ‘partly shared’ denotes that some T cell pools interact with one virus strain only, while other T cell pools are stimulated by more than one strain and clear cells infected by any of those strains. ‘None’ denotes that the compartment is removed from the model.

## Supporting information

S1 Fig The full set of synthetic data. (a) The line shows the simulated ‘true’ viral load for a single infection, with the arrow showing the time of exposure. The simulated viral loads with noise for the thirteen single infection ferrets are shown as crosses. The horizontal line indicates the observation threshold (10 RNA copy no./100*μ*L); observations below this threshold are plotted below this line. Values below the observation threshold were treated as censored. (b—g) For sequential infections with the labelled inter-exposure interval, the dashed and dotted lines show the simulated ‘true’ viral load for a primary and challenge infection respectively; the arrows show the times of the primary and challenge exposures. The simulated viral load with noise is shown as crosses. The sequential infection dataset consists of the viral load for the six sequential infection ferrets and one single infection ferret; the single infection dataset consists of the viral load for the thirteen single infection ferrets.
(PDF)

S2 Fig Trajectories for models (a) XI and (b) XT generated using 100 uniformly sampled parameter sets from the MCMC chains after burn-in, for the model fitted to sequential infection data. The green trajectory incorrectly attributed the delay observed in the baseline model to both target cell depletion and innate immunity.
(PDF)

S3 Fig Sequential infection data did not enable accurate prediction of the challenge viral load for a modified model where only one of the three innate immune mechanisms mediates cross-protection. The challenge viral load for the ‘true’ parameter values and a modified model where cross-protection is mediated by only one innate immune mechanism (models XI1–XI3, red line) was compared to the viral load for the baseline model (black line). (a—c) show results for models XI1–XI3 respectively. At a one-day inter-exposure interval, the delay in the baseline model occurred due to a combination of innate immune mechanisms 2 and 3. Prediction intervals for the viral load for models XI1–XI3 according to the model fitted to sequential infection data (blue areas) did not accurately recover the viral load according to the ‘true’ parameters. Hence, the fitted model did not attribute cross-immunity to the correct mechanisms of the innate immune response.
(PDF)

S4 Fig Compartmental diagram for two strains and three T cell pools. Cells infected with influenza strain 1 stimulate naive CD8^+^ T cells in pools 1 and 3, and are cleared by effector CD8^+^ T cells in these pools. Cells infected with influenza strain 2 stimulate naive CD8^+^ T cells in pools 2 and 3, and are cleared by effector CD8^+^ T cells in these pools.
(PDF)

S1 Text More details on the model fitting procedure. (PDF)

S2 Text Notes on biologically plausible ranges for the parameters *p*_*Vratio*_, *α* and *γ*, as provided in S1 Table. (PDF)

S1 File Two-strain model equations for the baseline and modified models, model modifications from our previous study, and compartmental diagrams for the modified models. (PDF)

S2 File Results for an additional set of parameters where the degree of cross-reactivity in the cellular adaptive immune response is high. (PDF)

S3 File Convergence diagnostics for the MCMC chains. (PDF)

S4 File Marginal posterior distributions for the model parameters. (PDF)

S5 File Results for a different set of ‘true’ parameters where the degree of cross-reactivity in the cellular adaptive immune response is low. (PDF)

S6 File Results for different noisy datasets with the same ‘true’ parameters. (PDF)

S7 File Results for a data set generated using a different model. (PDF)

S1 Table Viral replication parameter values and prior bounds. Note that the values and prior bounds are given in logarithmic space. For example, the value of log_10_ *R*_0_ was log_10_4.9 and the prior bounds were [0, 3]. Hence, the value of *R*_0_ was 4.9 and the prior bounds of *R*_0_ were [1, 1000]. *β* and *p*_*Vinf*_ were not directly fitted, but their values as recovered from Eqs 7 and 9 could not exceed the bounds given. Because total virions include infectious virions, the total virion decay rate should be slower than the infectious virion decay rate. Hence, the difference between the infectious and total virion decay rates *δ*_*Vinf*_ − *δ*_*Vtot*_, rather than the infectious virion decay rate *δ*_*Vinf*_, was fitted to ensure that the former quantity was positive. Notes on biologically plausible ranges for the parameters *p*_*tot*_, *α* and *γ* are given in S2 Text.
(PDF)

S2 Table Innate immune response parameter values and prior bounds. (PDF)

S3 Table Values and prior bounds for the cross-reactivity parameters in the cellular adaptive immune response. The number of infected cells for half-maximal stimulation of naive/memory CD8^+^ T cells *k*_*Cjq*_ and the clearance rate of infected cells by effector CD8^+^ T cells *κ*_*E*11_.
(PDF)

S4 Table Adaptive immune response and observation model parameter values and prior bounds. (PDF)
