# Supplementary material for: Sequential infection experiments for quantifying innate and adaptive immunity during influenza infection
Source: PLoS Comput Biol. 2019 Jan 17;15(1):e1006568. doi: 10.1371/journal.pcbi.1006568 (PMC6353225; doi:10.1371/journal.pcbi.1006568)
Supplement: S1 Fig — (a) The line shows the simulated ‘true’ viral load for a single infection, with the arrow showing the time of exposure. The simulated viral loads with noise for the thirteen single infection ferrets are shown as crosses. The horizontal line indicates the observation threshold (10 RNA copy no./100μL); observations below this threshold are plotted below this line. Values below the observation threshold were treated as censored. (b—g) For sequential infections with the labelled inter-exposure interval, the dashed and dotted lines show the simulated ‘true’ viral load for a primary and challenge infection respectively; the arrows show the times of the primary and challenge exposures. The simulated viral load with noise is shown as crosses. The sequential infection dataset consists of the viral load for the six sequential infection ferrets and one single infection ferret; the single infection dataset consists of the viral load for the thirteen single infection ferrets. (PDF) [file pcbi.1006568.s001.pdf]

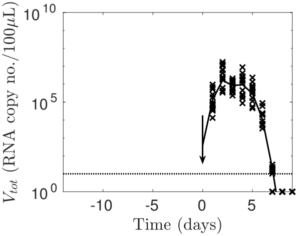

(a) single infection (challenge only)

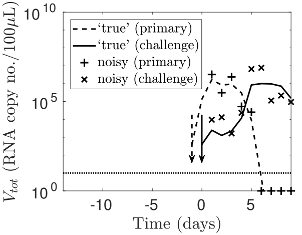

(b) 1-day interval

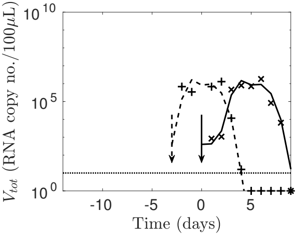

(c) 3-day interval

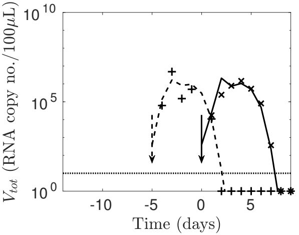

(d) 5-day interval

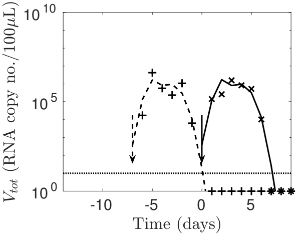

(e) 7-day interval

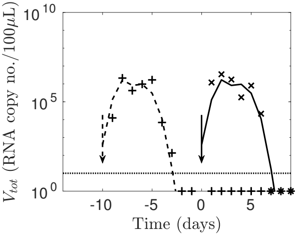

(f) 10-day interval

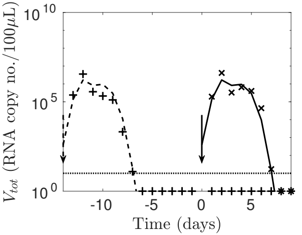

(g) 14-day interval
