# Supplementary figures and images for: Sequential infection experiments for quantifying innate and adaptive immunity during influenza infection
Source: PLoS Comput Biol. 2019 Jan 17;15(1):e1006568. doi: 10.1371/journal.pcbi.1006568 (PMC6353225; doi:10.1371/journal.pcbi.1006568)

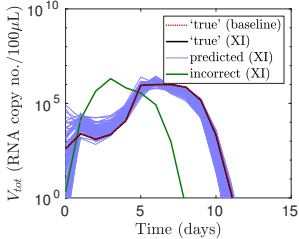

(a) XI

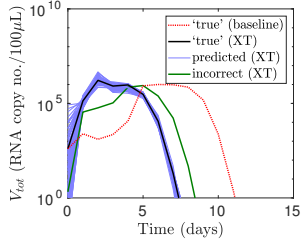

(b) XT

Supplement: S2 Fig — The green trajectory incorrectly attributed the delay observed in the baseline model to both target cell depletion and innate immunity. (PDF) [file pcbi.1006568.s002.pdf]

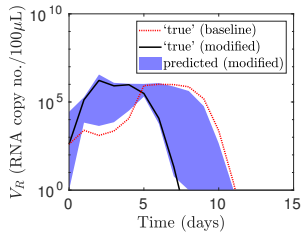

(a) XI1

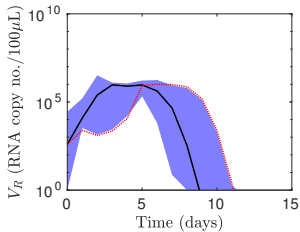

(b) XI2

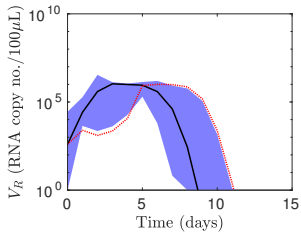

(c) XI3

Supplement: S3 Fig — The challenge viral load for the ‘true’ parameter values and a modified model where cross-protection is mediated by only one innate immune mechanism (models XI1–XI3, red line) was compared to the viral load for the baseline model (black line). (a—c) show results for models XI1–XI3 respectively. At a one-day inter-exposure interval, the delay in the baseline model occurred due to a combination of innate immune mechanisms 2 and 3. Prediction intervals for the viral load for models XI1–XI3 according to the model fitted to sequential infection data (blue areas) did not accurately recover the viral load according to the ‘true’ parameters. Hence, the fitted model did not attribute cross-immunity to the correct mechanisms of the innate immune response. (PDF) [file pcbi.1006568.s003.pdf]

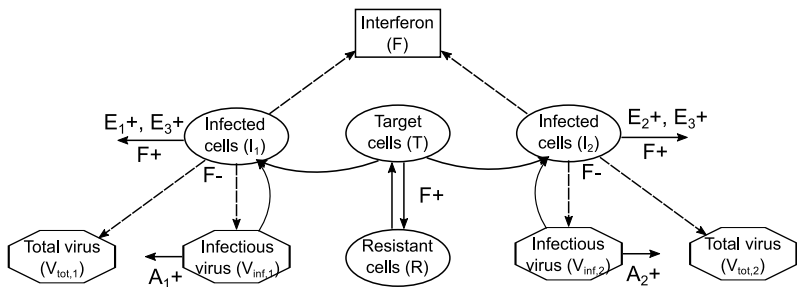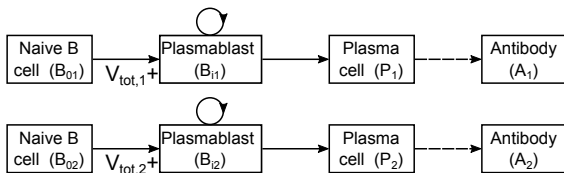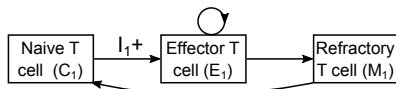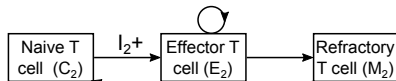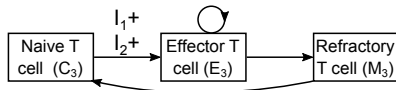

Supplement: S4 Fig — Cells infected with influenza strain 1 stimulate naive CD8+ T cells in pools 1 and 3, and are cleared by effector CD8+ T cells in these pools. Cells infected with influenza strain 2 stimulate naive CD8+ T cells in pools 2 and 3, and are cleared by effector CD8+ T cells in these pools. (PDF) [file pcbi.1006568.s004.pdf]
