# Supplementary material for: Sequential infection experiments for quantifying innate and adaptive immunity during influenza infection
Source: PLoS Comput Biol. 2019 Jan 17;15(1):e1006568. doi: 10.1371/journal.pcbi.1006568 (PMC6353225; doi:10.1371/journal.pcbi.1006568)
Supplement: S1 Text — (PDF) [file pcbi.1006568.s005.pdf]

# Sequential infection experiments for quantifying innate and adaptive immunity during influenza infection

Text S1:
More details on the model fitting procedure

## Determining the number of iterations for which to run the chains

The three MCMC chains were truncated at $k$ iterations, starting at $k = 10^4$. The first half of the chains were discarded as burn-in [1], and the 95% confidence interval for the potential scale reduction factor (PSRF) [2] for each parameter was calculated using the `gelman.diag` function in the `coda` package version 0.18-1 [3] in R version 3.3.2 [4]. If the upper bound of the 95% confidence interval for the PSRF was below 1.1 for all parameters, the chains were declared to have converged, and the process ended. All iterations of the three chains from $k/2+1$ onwards were then taken as draws from the joint posterior distribution. Otherwise, $k$ was increased by $10^4$, and the process was repeated (with the original chains starting from the end of the calibrating stage). If the full number of iterations in the chain was reached and convergence still had not occurred, the chains were run for more iterations.

## Tuning of the proposal distributions

A calibration period was introduced where the widths of the proposal distributions were adjusted. Initially, the proposal distribution for each parameter was set to be a uniform distribution centred at the current value of the parameter, of width equal to the bounds of the marginal prior distribution for that parameter. During calibration, the proposal distribution remained centred at the most current value of the parameter; only the width of each distribution changed. Every 100 iterations, the proportion of acceptance events during those 100 iterations for each parameter was checked. If the acceptance proportion was too low (a threshold was set at 0.3), the width of the proposal distribution was multiplied by 0.75; if the acceptance proportion was too high (above 0.5), the width of the proposal distribution was multiplied by 1.5. These thresholds were chosen because when each parameter is sampled in turn, for specific classes of likelihood functions, the optimal acceptance rate has been shown to be 0.44 [5]. However,

we emphasise that tuning the acceptance rate merely enabled convergence to occur within fewer iterations; attaining the optimal acceptance rate precisely was not essential. The calibration stage lasted for $10^4$ iterations. The widths of the proposal distributions then stayed fixed, and the initial $10^4$ iterations were discarded.

# Calibration of temperatures for parallel tempering

For each simulation, $w = 7$ parallel chains were used, with temperatures between 1 and 50 evenly spread in log space. Every 100 iterations, for each pair of chains $k, k+1$ for $k = m, m+2, \ldots, w-1$, the proportion of proposed swaps during those 100 iterations which were accepted was checked. ($m$ alternated between 1 and 2 every 100 iterations.) If the acceptance proportion was too low (below 0.1), the difference in temperatures $T_k$ and $T_{k+1}$ was multiplied by a factor of 0.75 (the temperatures for chains $k+1, \ldots, w$ are shifted along), and if the acceptance proportion was too high (above 0.4), the difference in temperatures was multiplied by a factor of 1.5. This procedure was based on an optimal acceptance proportion of 20–23% [6, 7].

# References


1. Gelman A, Carlin JB, Stern HS, Rubin DB. Bayesian Data Analysis. 2nd ed. London, UK: Chapman & Hall/CRC; 2004.

2. Gelman A, Rubin DB. Inference from iterative simulation using multiple sequences. Stat Sci. 1992;7(4):457–472.

3. Plummer M, Best N, Cowles K, Vines K. CODA: convergence diagnosis and output analysis for MCMC. R News. 2006;6(1):7–11.

4. R Core Team. R: A Language and Environment for Statistical Computing; 2016. Available from: `https://www.R-project.org/`.

5. Gelman A, Roberts GO, Gilks WR. In: Bernardo JM, Berger JO, Dawid AP, Smith AFM, editors. Efficient Metropolis jumping rules. New York: Oxford University Press; 1995. p. 599–607.

6. Rathore N, Chopra M, De Pablo JJ. Optimal allocation of replicas in parallel tempering simulations. J Chem Phys. 2005;122(2):024111. doi:10.1063/1.1831273.

7. Kone A, Kofke DA. Selection of temperature intervals for parallel-tempering simulations. J Chem Phys. 2005;122(20):206101. doi:10.1063/1.1917749.
