# Supplementary material for: Sequential infection experiments for quantifying innate and adaptive immunity during influenza infection
Source: PLoS Comput Biol. 2019 Jan 17;15(1):e1006568. doi: 10.1371/journal.pcbi.1006568 (PMC6353225; doi:10.1371/journal.pcbi.1006568)
Supplement: S2 Text — (PDF) [file pcbi.1006568.s006.pdf]

Sequential infection experiments for quantifying innate and adaptive immunity during influenza infection

Text S2:
Notes on biologically plausible ranges for the parameters p_{Vratio}, α and γ

First, we examine the parameter α. The concentration of virions in nasal wash is lower than the concentration of virions in the respiratory tract by a factor of $a = 1 - 100$ [1]. The volume of the ferret upper respiratory tract, which was assumed to be the site of infection, is approximately $v = 1$mL [2], while the concentration of nasal wash was reported per $b = 0.1$mL [3]. We assumed that 1 RNA copy number corresponded to one (infectious or non-infectious) virion. Hence, the number of RNA copies/100μL of nasal wash corresponding to one virion in the respiratory tract (α) was $(b/v)/a$. Based on the bounds for a and b, the bounds for α were then $[10^{-3}, 10^{-1}]$.

Next, we examine the parameter γ. Petrie *et al.* [4] used the bounds $[10^0, 10^5]$ to fit the initial ratio of total to infectious virions. These bounds were in units of RNA copy number/TCID$_{50}$ (f). These bounds were based on the variability across different inocula given to donor ferrets in the experiments by Butler *et al.* [5]; the study by Petrie *et al.* [4] analysed data from these experiments. However, in our study, γ was required to be in units of RNA copy number/virion, as we accounted for the loss of a single virion due to its infecting a target cell. Assays to measure the amount of infectious virus cannot measure the number of virions directly; hence, we needed to estimate the conversion factor between TCID$_{50}$ and virions (h). At the minimum, one virion was required to establish an infection; hence, the lower bound for the number of virions corresponding to 1 TCID$_{50}$ was 1 virion. Handel *et al.* [1] estimated that 1 TCID$_{50}$ corresponds to 1–100 virions, so the bounds for h were $[1, 100]$. Hence, the initial ratio of total to infectious virions in the respiratory tract in units of RNA copy number/virion (γ), which by definition had a lower bound of 1, was $\gamma = f/h$. Based on the bounds for f and h, the bounds for γ were then $[10^0, 10^5]$.

Lastly, we tackle the parameter p_{Vratio}. Petrie *et al.* [4] used the bounds $[10^0, 10^6]$ to fit the ratio of production of total to infectious virions, in units of RNA copy number/TCID$_{50}$ (q). These bounds were based on the variability of this ratio in the data provided by Butler *et al.* [5]. The ratio of production of total to infectious virions in units of RNA copy number/virion (p_{Vratio}), which also by definition had a lower bound of 1, was then $p_{Vratio} = q/h$. Based on

the bounds for h and q, the bounds for p_{Vratio} were then $[10^0, 10^6]$.

References

1. Handel A, Longini Jr IM, Antia R. Neuraminidase inhibitor resistance in influenza: assessing the danger of its generation and spread. PLoS Comput Biol. 2007;3(12):e240. doi:10.1371/journal.pcbi.0030240.

2. Petrie SM, Guarnaccia T, Laurie KL, Hurt AC, McVernon J, McCaw JM. Reducing uncertainty in within-host parameter estimates of influenza infection by measuring both infectious and total viral load. PLoS ONE. 2013;8(5):e64098. doi:10.1371/journal.pone.0064098.

3. Laurie KL, Guarnaccia TA, Carolan LA, Yan AWC, Aban M, Petrie S, et al. Interval between infections and viral hierarchy are determinants of viral interference following influenza virus infection in a ferret model. J Infect Dis. 2015;212(11):1701–1710. doi:10.1093/infdis/jiv260.

4. Petrie SM, Butler J, Barr IG, McVernon J, Hurt AC, McCaw JM. Quantifying relative within-host replication fitness in influenza virus competition experiments. J Theor Biol. 2015;382:259–271. doi:10.1016/j.jtbi.2015.07.003.

5. Butler J, Hooper KA, Petrie S, Lee R, Maurer-Stroh S, Reh L, et al. Estimating the fitness advantage conferred by permissive neuraminidase mutations in recent oseltamivir-resistant A(H1N1)pdm09 influenza viruses. PLoS Pathog. 2014;10(4):e1004065. doi:10.1371/journal.ppat.1004065.
