# Supplementary material for: Sequential infection experiments for quantifying innate and adaptive immunity during influenza infection
Source: PLoS Comput Biol. 2019 Jan 17;15(1):e1006568. doi: 10.1371/journal.pcbi.1006568 (PMC6353225; doi:10.1371/journal.pcbi.1006568)
Supplement: S1 File — (PDF) [file pcbi.1006568.s007.pdf]

Sequential infection experiments for quantifying innate and adaptive immunity during influenza infection

File S1:
Two-strain model equations for the baseline and modified models, model modifications from our previous study, and compartmental diagrams for the modified models

The two-strain baseline model

The model equations are given by

$$
\begin{aligned}
\frac{dT}{dt} &= g(T+R)\left(1-\frac{T+R+\sum_{q=1}^{2} I_q}{T_0}\right) - \sum_{q=1}^{2} \beta V_{inf,q} T + \rho R - \phi F T,\\
\frac{dI_q}{dt} &= \beta V_{inf,q} T - \left(\delta_I + \kappa_F F + \sum_{j=1}^{J} \kappa_{Ejq} E_j\right) I_q, \qquad q=1,2,\\
\frac{dV_{inf,q}}{dt} &= \frac{p_{Vinf}}{1+sF} I_q - (\delta_{Vinf} + \kappa_A A_q + \beta T) V_{inf,q},\\
\frac{dV_{tot,q}}{dt} &= \frac{p_{Vinf} p_{Vratio} \alpha}{1+sF} I_q - \delta_{Vtot} V_{tot,q} - \alpha \beta T V_{inf,q},\\
\frac{dR}{dt} &= \phi F T - \rho R,\\
\frac{dF}{dt} &= \sum_{q=1}^{2} I_q - \delta_F F,\\
\frac{dB_{0q}}{dt} &= -\frac{V_{tot,q}}{k_B + V_{tot,q}} \beta_B B_{0q},\\
\frac{dB_{1q}}{dt} &= \frac{V_{tot,q}}{k_B + V_{tot,q}} \beta_B B_{0q} - \left(\frac{n_B}{\tau_B} + \delta_B\right) B_{1q},\\
\frac{dB_{iq}}{dt} &= \frac{2 n_B B_{i-1,q}}{\tau_B} - \left(\frac{n_B}{\tau_B} + \delta_B\right) B_{iq}, \qquad i=2,...,n_B,\\
\frac{dP_q}{dt} &= \frac{2 n_B B_{n_B,q}}{\tau_B} - \delta_B P_q,\\
\frac{dA_q}{dt} &= P_q - \delta_A A_q,\\
\frac{dC_j}{dt} &= \frac{M_j}{\tau_M} - \frac{\sum_{q=1}^{2} I_q / k_{Cjq}}{1+\sum_{q=1}^{2} I_q / k_{Cjq}} \beta_C C_j,\\
\frac{dE_{1j}}{dt} &= \frac{\sum_{q=1}^{2} I_q / k_{Cjq}}{1+\sum_{q=1}^{2} I_q / k_{Cjq}} \beta_C C_j - \left(\frac{n_E}{\tau_E} + \delta_E\right) E_{1j},\\
\frac{dE_{ij}}{dt} &= \frac{2 n_E E_{i-1,j}}{\tau_E} - \left(\frac{n_E}{\tau_E} + \delta_E\right) E_{ij}, \qquad i=2,...,n_E-1,\\
\frac{dE_{n_E}}{dt} &= \frac{2 n_E E_{n_E-1,j}}{\tau_E} - \delta_E E_{n_E},\\
E_j &= \sum_{i=1}^{n_E} E_{ij},\\
\frac{dM_j}{dt} &= \epsilon \delta_E E_{n_E} - \delta_E M_j - \frac{M_j}{\tau_M}.
\end{aligned}
\tag{1}
$$

For the baseline model, the number of T cell pools is $J = 3$. To reduce the number of independent cross-reactivity parameters, we impose the relationship

$\kappa_{Ejq} = \kappa_{E11} k_{C11} / k_{Cjq}$.

Modifications to the model by Yan *et al.* [1]

The model in Eq. 1 was modified from a previous study [1]. The model has been documented completely in the present study, but for clarity we document the modifications made.

First, we simplified the memory CD8$^+$ T cell compartments by assuming that parameter values for central memory CD8$^+$ T cells were identical to those of naive CD8$^+$ T cells.

Second, one of the aims of the simulation-estimation study was to see whether fitting the model to experimental data would allow us to disentangle the three innate immune mechanisms included in the model by Cao *et al.* [2]. The model in the previous study [1] only included one innate immune mechanism as innate immunity was not the focus of that study. In the present study, we re-included all three innate immune mechanisms.

Third, as discussed in the Materials and Methods section of the main text, in the experiments conducted by Laurie *et al.* [3], the concentration of total viral load, rather than the number of infectious virions, was measured. Hence, we included an additional equation for total viral load concentration.

Fourth, the only compartment in the model for which time series data was available for all ferrets is the total viral load V_{totq}. Given this limitation, we combined some model parameters and normalised some model compartments, to reduce the number of model parameters and increase structural identifiability of the model.

In our previous study [1], the strength of cellular adaptive immunity was a function of the avidity a_j and the epitope abundance d_{jq}, but these two quantities only appeared in the combinations

$$\begin{aligned} k_{Cjq} &= \frac{\tilde{k}_C}{a_j d_{jq}}, \\ \kappa_{Ejq} &= \tilde{\kappa}_E a_j d_{jq}, \end{aligned} \tag{2}$$

where $\tilde{k}_C$ and $\tilde{\kappa}_E$ were baseline values for the number of infected cells required for half-maximal stimulation of naive CD8$^+$ T cells and the clearance rate of infected cells by effector CD8$^+$ T cells respectively. Hence, we need only specify k_{Cjq} rather than a_j and d_{jq} separately. Furthermore, of the parameters κ_{Ejq}, we need only specify κ_{E11}, from which the remaining values κ_{Ejq} can be obtained from the relation $\kappa_{Ejq} = \kappa_{E11} \frac{k_{C11}}{k_{Cjq}}$.

The compartments F, B, P, A, C, E and M were not measured directly, so we normalised them to reduce the number of model parameters. The adaptive immune compartments were scaled such that the initial values of C and B_0 are 1. F was scaled such that the production rate of interferon by infected cells is 1 day^{-1}.

Models XC, XI, XT and XIT

Figure A shows compartmental diagrams for models XC, XI, XT and XIT. In model XC, cross-protection is mediated by cellular adaptive immunity only, and not target cell depletion or innate immunity. Unlike the baseline model, type I interferon F_q are strain-specific. Cells infected with strain q induce the production of interferon specific to that strain. The effects of interferon F_q — rendering target cells temporarily resistant to infection; lowering the production rate of virions; and increasing the death rate of cells — only apply to strain q. In addition, each strain now targets a separate pool of uninfected cells; the size of each target cell pool is T_0 (identical for both strains). This alternative model is not meant to reflect a biologically realistic situation; however, it enables *in silico* thought experiments to determine, for a given set of parameters, the contribution of each immune component to cross-protection.

Explicitly, the changed equations (relative to Eq. 1) for model XC are given by

$$
\begin{aligned}
\frac{dT_q}{dt} &= g\left(T_q + R_q\right)\left(1 - \frac{T_q + R_q + I_q}{T_0}\right) - \beta T_q V_{inf,q} - \phi F_q T_q + \rho R_q, \qquad q = 1, 2,\\
\frac{dI_q}{dt} &= \beta V_{inf,q} T_q - \left(\delta_I + \kappa_F F_q + \sum_{j=1}^{J} \kappa_{Ejq} E_j\right) I_q,\\
\frac{dV_{inf,q}}{dt} &= \frac{p_{Vinf}}{1 + sF_q} I_q - \left(\delta_{Vinf} + \kappa_A A_q + \beta T_q\right) V_{inf,q},\\
\frac{dV_{tot,q}}{dt} &= \frac{p_{Vinf} p_{Vratio} \alpha}{1 + sF_q} I_q - \delta_{Vtot} V_{tot,q} - \alpha \beta T_q V_{inf,q},\\
\frac{dR_q}{dt} &= \phi F_q T_q - \rho R_q,\\
\frac{dF_q}{dt} &= I_q - \delta_F F_q.
\end{aligned} \tag{3}
$$

The equations for B_{iq}, P_q, A_q, C_j, E_{ij} and M_j remain unchanged.

In model XI, cross-protection is mediated by innate immunity, but not target cell depletion or cellular adaptive immunity. The model is altered from the baseline model such that the number of T cell pools is $J = 4$. T cell pools 3 and 4 have the same parameters as pool 3 in the baseline model, but T cell pool 3 is stimulated by, and targets, cells infected with strain 1 only, while T cell pool 4 is stimulated by, and targets, cells infected with strain 2 only. (Explicitly, the cross-reactivity parameters for T cell pools 3 and 4 are $k_{C31} = k_{C42}$ equal to $k_{C31} = k_{C32}$ in the baseline model; $k_{C32} = k_{C41} = \infty$; $\kappa_{E31} = \kappa_{E42}$ equal to $\kappa_{E31} = \kappa_{E32}$ in the baseline model; and $\kappa_{E32} = \kappa_{E42} = 0$.) The cross-reactivity parameters for pools 1 and 2 remain the same. In addition, each strain now has its own target cell pool.

The equations for model XI which are altered relative to Eq. 1 are

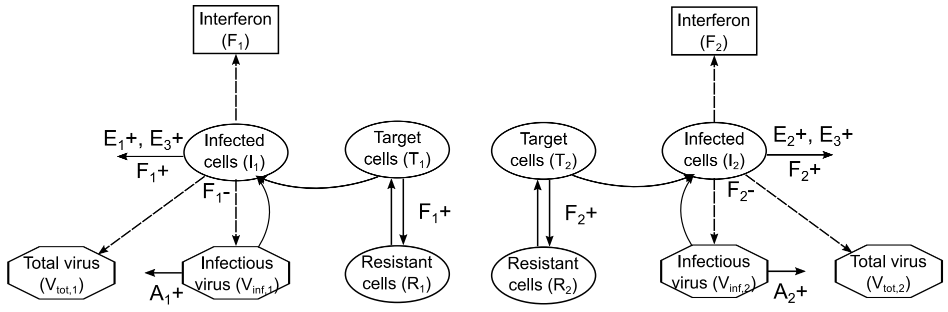

(i) Model XC

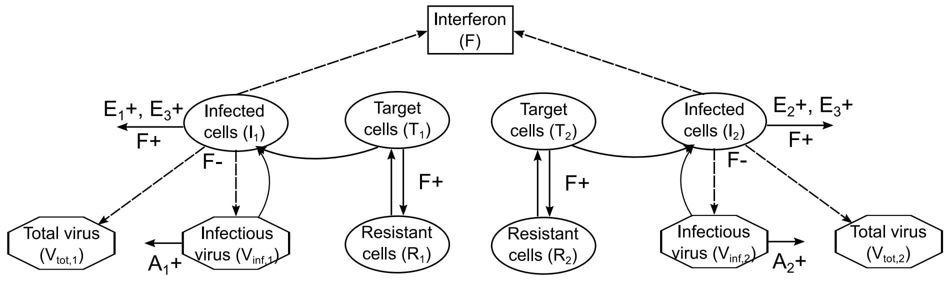

(ii) Model XI

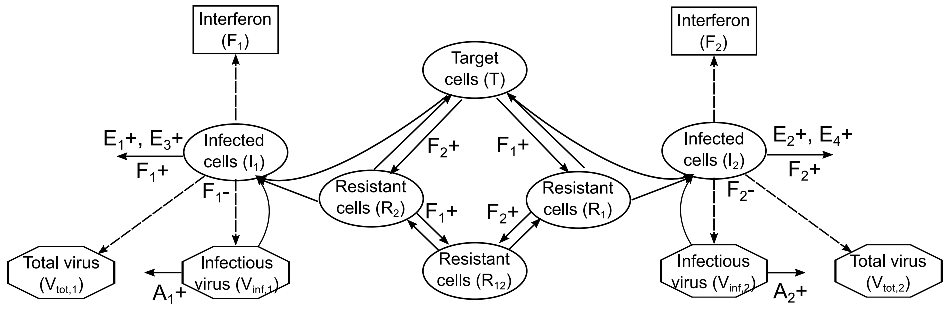

(iii) Model XT

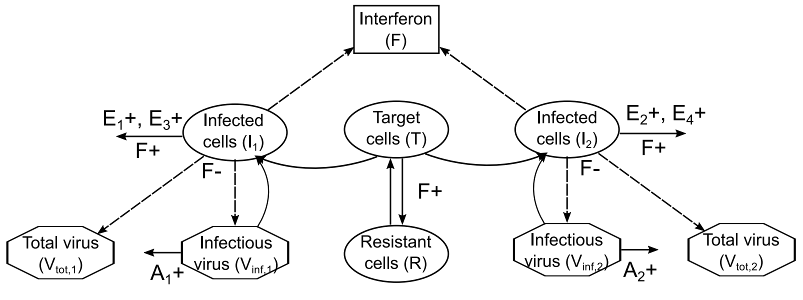

(iv) Model XIT

Figure A: **Compartmental diagrams for models XC, XI, XT and XIT.**

$$
\begin{aligned}
\frac{dT_q}{dt} &= g\left(T_q + R_q\right)\left(1 - \frac{T_q + R_q + I_q}{T_0}\right) - \beta T_q V_{inf,q} - \phi F T_q + \rho R_q, \qquad q = 1, 2,\\
\frac{dI_q}{dt} &= \beta V_{inf,q} T_q - \left(\delta_I + \kappa_F F + \sum_{j=1}^{J} \kappa_{Ejq} E_j\right) I_q,\\
\frac{dV_{inf,q}}{dt} &= \frac{p_{Vinf}}{1 + sF} I_q - \left(\delta_{Vinf} + \kappa_A A_q + \beta T_q\right) V_{inf,q},\\
\frac{dV_{tot,q}}{dt} &= \frac{p_{Vinf} p_{Vratio} \alpha}{1 + sF} I_q - \delta_{Vtot} V_{tot,q} - \alpha \beta T_q V_{tot,q},\\
\frac{dR_q}{dt} &= \phi F T_q - \rho R_q.
\end{aligned}
\tag{4}
$$

In the model where cross-protection is mediated by target cell depletion only, the model is altered from the baseline model such that the number of T cell pools is $J = 4$. In addition, type I interferon F_q are strain-specific. Cells infected with strain q induce the production of interferon specific to that strain. The effects of interferon F_q — rendering target cells temporarily resistant to infection; lowering the production rate of virions; and increasing the death rate of cells — only apply to strain q. However, the virus strains still share a target cell pool, so to implement target cells becoming temporarily resistant to infection with strain q, we now explicitly track refractory cells resistant to each strain, R_q, and refractory cells resistant to both strains, R_{12}.

The changed equations (relative to Eq. 1) for model XT are

$$
\begin{aligned}
\frac{dT}{dt} &= g\left(T + \sum_{q=1}^{2} R_q + R_{12}\right)\left[1 - \frac{T + \sum_{q=1}^{2}(R_q + I_q) + R_{12}}{T_0}\right] \\
&\quad - \beta T \sum_{q=1}^{2} V_{inf,q} - \phi \sum_{q=1}^{2} F_q T + \rho \sum_{q=1}^{2} R_q, \\
\frac{dI_q}{dt} &= \beta V_{inf,q}\left(T + \sum_{q' \neq q} R_{q'}\right) - \left(\delta_I + \kappa_F F_q + \sum_{j=1}^{J} \kappa_{Ejq} E_j\right) I_q, \qquad q = 1,2 \\
\frac{dV_{inf,q}}{dt} &= \frac{p_{Vinf}}{1 + sF_q} I_q - \left(\delta_{Vinf} + \kappa_A A_q + \beta T\right) V_{inf,q}, \\
\frac{dV_{tot,q}}{dt} &= \frac{p_{Vinf} p_{Vratio} \alpha}{1 + sF_q} I_q - \delta_{Vtot} V_{tot,q} - \alpha \beta T V_{inf,q}, \\
\frac{dR_q}{dt} &= \phi F_q T - \rho R_q - \phi \sum_{q' \neq q} F_{q'} R_q + \rho R_{12} - \beta \sum_{q' \neq q} V_{inf,q'} R_q, \\
\frac{dR_{12}}{dt} &= \phi \sum_{q=1}^{2} \sum_{q' \neq q} F_{q'} R_q - 2\rho R_{12}, \\
\frac{dF_q}{dt} &= I_q - \delta_F F_q.
\end{aligned}
$$

In the model where cross-protection is mediated by target cell depletion and/or innate immunity, but not cellular adaptive immunity (model XIT), the cellular adaptive immune response is no longer cross-reactive. Like for model XI, the model is altered from the baseline model such that the number of T cell pools is $J = 4$. However, like the baseline model, target cells are shared between the two strains. Hence, the alterations in Eq. 4 are not made for model XIT.

Models where only one innate immune mechanism mediates cross-protection

We define the models XI1–XI3 (Fig. B) such that in each model, cells infected with strain q induce the production of interferon specific to strain q. In model XIy, cross-protection is only mediated by innate immune mechanism y, and not by the other innate immune mechanisms, target cell depletion, or cellular adaptive immunity. To recap, the three mechanisms are:

1. rendering target cells temporarily resistant to infection ($T \rightarrow R$);
2. decreasing the production rate of virions from infected cells; and
3. increasing the decay rate of infected cells.

Equations 5–7 show the equations which are changed in models XI1–XI3 relative to the baseline model (Eq. 1).

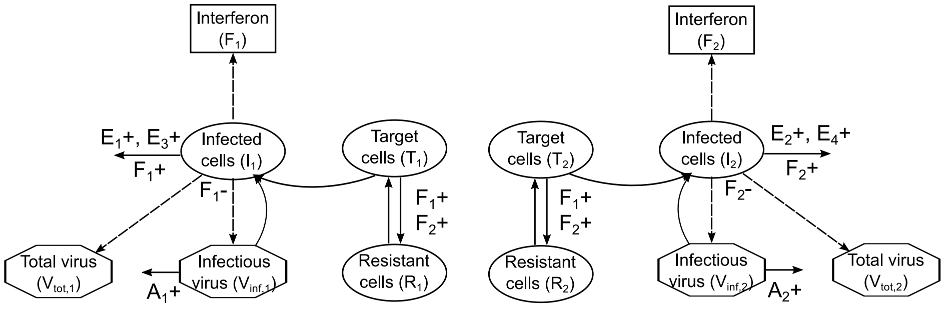

(i) Model XI1: cross-protection mediated by interferon rendering target cells temporarily resistant to infection

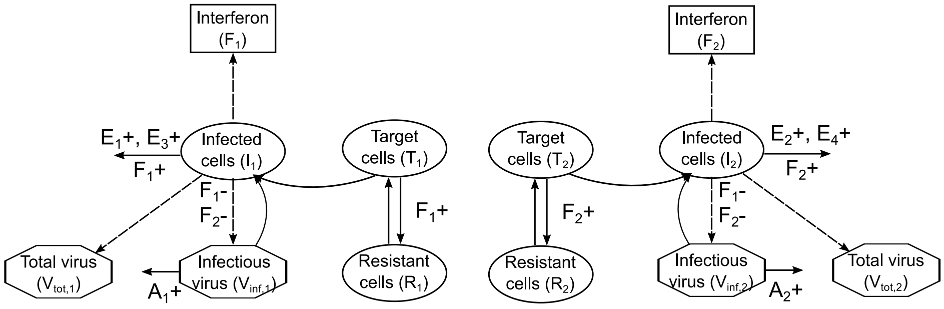

(ii) Model XI2: cross-protection mediated by interferon decreasing the production rate of virions from infected cells

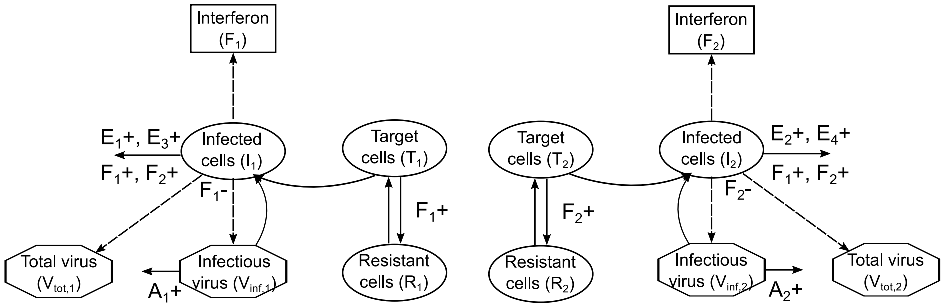

(iii) Model XI3: cross-protection mediated by interferon increasing the clearance rate of infected cells

Figure B: Three alternative models, where in model XIy, cross-protection is only mediated by innate immune mechanism y, and not by the other innate immune mechanisms, target cell depletion, or cellular adaptive immunity.

$$
\begin{aligned}
\frac{dT_q}{dt} &= g\left(T_q + R_q\right)\left(1 - \frac{T_q + R_q + I_q}{T_0}\right) - \beta T_q V_{inf,q} - \phi\left(\sum_{q'=1}^{2} F_{q'}\right) T_q + \rho R_q, \\
\frac{dI_q}{dt} &= \beta V_{inf,q} T_q - \left(\delta_I + \kappa_F F_q + \sum_{j=1}^{J} \kappa_{Ejq} E_j\right) I_q, \\
\frac{dV_{inf,q}}{dt} &= \frac{p_{Vinf}}{1 + sF_q} I_q - \left(\delta_{Vinf} + \kappa_A A_q + \beta T_q\right) V_{inf,q}, \\
\frac{dV_{tot,q}}{dt} &= \frac{p_{Vinf} p_{Vratio} \alpha}{1 + sF_q} I_q - \delta_{Vtot} V_{tot,q} - \alpha\beta T_q V_{inf,q}, \qquad q = 1, 2, \\
\frac{dR_q}{dt} &= \phi\left(\sum_{q'=1}^{2} F_{q'}\right) T_q - \rho R_q, \\
\frac{dF_q}{dt} &= I_q - \delta_F F_q.
\end{aligned} \tag{5}
$$

$$
\begin{aligned}
\frac{dT_q}{dt} &= g\left(T_q + R_q\right)\left(1 - \frac{T_q + R_q + I_q}{T_0}\right) - \beta T_q V_{inf,q} - \phi F_q T_q + \rho R_q, \\
\frac{dI_q}{dt} &= \beta V_{inf,q} T_q - \left(\delta_I + \kappa_F F_q + \sum_{j=1}^{J} \kappa_{Ejq} E_j\right) I_q, \\
\frac{dV_{inf,q}}{dt} &= \frac{p_{Vinf}}{1 + s\left(\sum_{q'=1}^{2} F_{q'}\right)} I_q - \left(\delta_{Vinf} + \kappa_A A_q + \beta T_q\right) V_{inf,q}, \\
\frac{dV_{tot,q}}{dt} &= \frac{p_{Vinf} p_{Vratio} \alpha}{1 + s\left(\sum_{q'=1}^{2} F_{q'}\right)} I_q - \delta_{Vtot} V_{tot,q} - \alpha\beta T_q V_{inf,q}, \qquad q = 1, 2, \\
\frac{dR_q}{dt} &= \phi F_q T_q - \rho R_q, \\
\frac{dF_q}{dt} &= I_q - \delta_F F_q.
\end{aligned} \tag{6}
$$

$$
\begin{aligned}
\frac{dT_q}{dt} &= g\left(T_q + R_q\right)\left(1 - \frac{T_q + R_q + I_q}{T_0}\right) - \beta T_q V_q - \phi F_q T_q + \rho R_q, \\
\frac{dI_q}{dt} &= \beta V_q T_q - \left[\delta_I + \kappa_F \left(\sum_{q'=1}^{2} F_{q'}\right) + \sum_{j=1}^{J} \kappa_{Ejq} E_j\right] I_q, \\
\frac{dV_{inf,q}}{dt} &= \frac{p_{Vinf}}{1 + sF_q} I_q - \left(\delta_{Vinf} + \kappa_A A_q + \beta T_q\right) V_{inf,q}, \\
\frac{dV_{tot,q}}{dt} &= \frac{p_{Vinf} p_{Vratio} \alpha}{1 + sF_q} I_q - \delta_{Vtot} V_{tot,q} - \alpha \beta T_q V_{inf,q}, \qquad q = 1, 2, \\
\frac{dR_q}{dt} &= \phi F_q T_q - \rho R_q, \\
\frac{dF_q}{dt} &= I_q - \delta_F F_q.
\end{aligned}
\tag{7}
$$

References

1. Yan AWC, Cao P, Heffernan JM, McVernon J, Quinn KM, La Gruta NL, et al. Modelling cross-reactivity and memory in the cellular adaptive immune response to influenza infection in the host. J Theor Biol. 2017;413:34–49. doi:10.1016/j.jtbi.2016.11.008.

2. Cao P, Yan AWC, Heffernan JM, Petrie S, Moss RG, Carolan LA, et al. Innate immunity and the inter-exposure interval determine the dynamics of secondary influenza virus infection and explain observed viral hierarchies. PLoS Comput Biol. 2015;11(8):e1004334. doi:10.1371/journal.pcbi.1004334.

3. Laurie KL, Guarnaccia TA, Carolan LA, Yan AWC, Aban M, Petrie S, et al. Interval between infections and viral hierarchy are determinants of viral interference following influenza virus infection in a ferret model. J Infect Dis. 2015;212(11):1701–1710. doi:10.1093/infdis/jiv260.
