# Supplementary material for: Sequential infection experiments for quantifying innate and adaptive immunity during influenza infection
Source: PLoS Comput Biol. 2019 Jan 17;15(1):e1006568. doi: 10.1371/journal.pcbi.1006568 (PMC6353225; doi:10.1371/journal.pcbi.1006568)
Supplement: S2 File — (PDF) [file pcbi.1006568.s008.pdf]

Sequential infection experiments for quantifying innate and adaptive immunity during influenza infection

File S2:
Additional results for a sequential infection, high cross-reactivity dataset

The sequential infection, high cross-reactivity dataset

This file shows results corresponding to the main text, for a sequential infection dataset where the degree of cross-reactivity in the cellular adaptive immunity is high. For short inter-exposure intervals (1–3 days), the initial growth of the challenge virus was suppressed, but both infections resolved simultaneously; for medium inter-exposure intervals (5–7 days), the challenge infection was prevented; and for long inter-exposure intervals (7–14 days), the challenge infection was shortened. These features of the synthetic data match the qualitative results by Laurie *et al.* [1] for infection with heterologous influenza A strains.

We used the same parameter values for the low and high cross-reactivity datasets, except for the cross-reactivity parameters. Instead of the parameter values given in Table S3, $\log_{10} k_{C11} = \log_{10} k_{C22} = 6$ and $\log_{10} k_{C13} = \log_{10} k_{C23} = 5.05$. The single infection data is thus the same for the low and high cross-reactivity datasets.

Figure A shows a subset of the synthetic data.

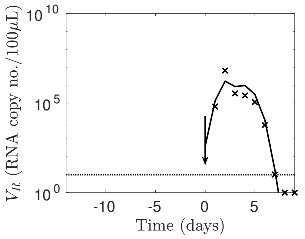

(i) single infection (challenge only)

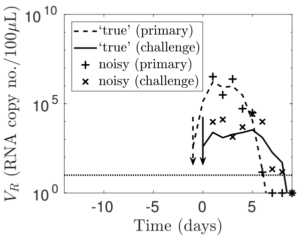

(ii) 1-day interval

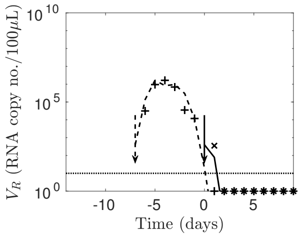

(iii) 7-day interval

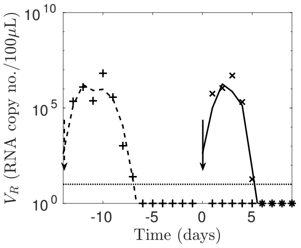

(iv) 14-day interval

Figure A: **A subset of the synthetic data.** (i) The line shows the simulated 'true' viral load for a single infection, with the arrow showing the time of exposure. The simulated viral load with noise is shown as crosses. The horizontal line indicates the observation threshold (10 RNA copy no./100μL); observations below this threshold are plotted below this line. Values below the observation threshold were treated as censored. (ii–iv) For sequential infections with the labelled inter-exposure interval, the dashed and dotted lines show the simulated 'true' viral load for a primary and challenge infection respectively; the arrows show the times of the primary and challenge exposures. The simulated viral load with noise is shown as crosses.

Results

Verification of the fitting procedure

Paralleling the main text, we first verified that our model fitting procedure recovers the simulated 'true' viral load. As the single infection data is the same for the low and high cross-reactivity datasets, we focused on results for sequential infection data.

Figure B presents 95% credible intervals for the viral load. The credible intervals included the 'true' viral load, confirming accurate recovery.

Comparing the immunological information in each dataset

Next, we compared the behaviour of the fitted models to the behaviour of the 'true' parameters, to determine the information in each dataset on

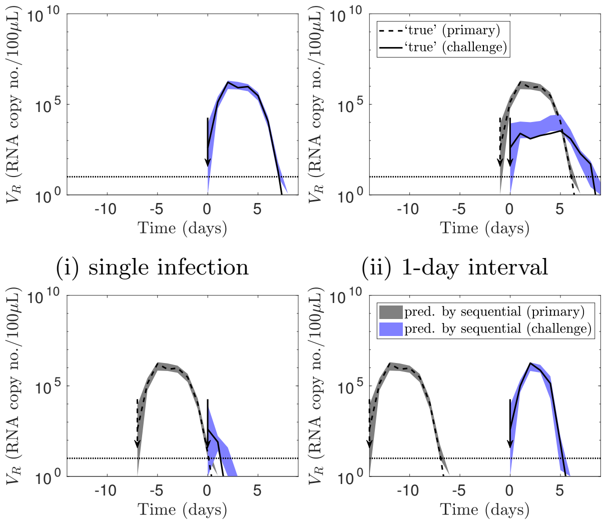

(i) single infection (ii) 1-day interval

(iii) 7-day interval (iv) 14-day interval

Figure B: **Verification that the fitting procedure recovered the viral load.** (i) For a single infection, the shaded area is the 95% credible interval for the viral load (in the absence of noise), as predicted by the models fitted to the sequential infection data. (ii–iv) For sequential infections with the labelled inter-exposure interval, the grey and blue areas show the 95% credible intervals for the primary and challenge viral load respectively, predicted by the model fitted to sequential infection data. The other elements of the figure are identical to Fig 1 in the main text: the dashed and dotted lines show the simulated 'true' viral load for a primary and challenge infection respectively; the arrows show the times of the primary and challenge exposures; and the horizontal line indicates the observation threshold.

- the effect of each immune component in controlling a single infection;
- cross-protection between strains; and
- each immune component's contribution to cross-protection.

The effect of each immune component in controlling a single infection

In Fig. C, we removed various immune components from the model. We arrived at the same conclusion as in the main text. The sequential infection, high cross-reactivity dataset enabled recovery of the times at which each immune component took effect. It also enabled accurate prediction of the viral load when adaptive immunity was suppressed. However, it did not enable accurate prediction of the viral load when innate, humoral, cellular adaptive immunity or all immunity was suppressed.

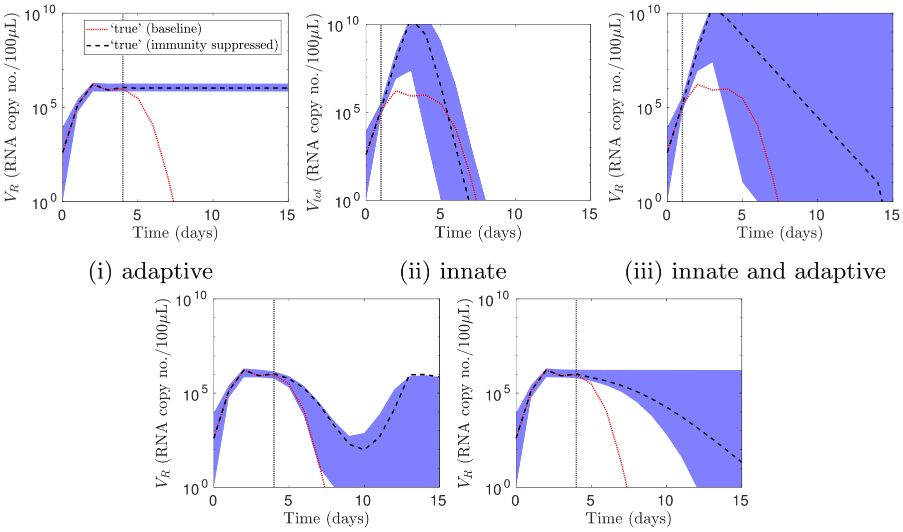

(i) adaptive (ii) innate (iii) innate and adaptive

(iv) humoral adaptive (v) cellular adaptive

Figure C: **Predicting the viral load for a single infection when various immune components were absent.** The vertical lines indicate, for the 'true' parameter values, the times at which the immune components labelled under each panel took effect. These times were determined by when the viral load for the baseline model (red dotted line) deviated from the viral load when the immune components were absent (black dashed line). These times could be recovered using sequential infection data in all of the panels (95% prediction intervals for the viral load in blue). In addition, the viral load when adaptive immunity was suppressed was accurately predicted (i). However, the viral load was not accurately predicted in the remaining scenarios (ii–v). Prediction intervals were constructed without measurement noise.

Cross-protection between strains

Given the above mixed results, we then tested whether sequential infection data accurately captured the timing and extent of cross-protection, by simulating the viral load for inter-exposure intervals other than those where data was provided.

Figure D shows prediction intervals for the challenge viral load for inter-exposure intervals of 2, 6 and 20 days. Like in the main text, the blue areas, which correspond to the fitted model, accurately predict the viral load for the challenge strain. These predictions include (i) co-infection with the two strains; (ii) prevention of the challenge infection; and (iii) shortening of the challenge infection.

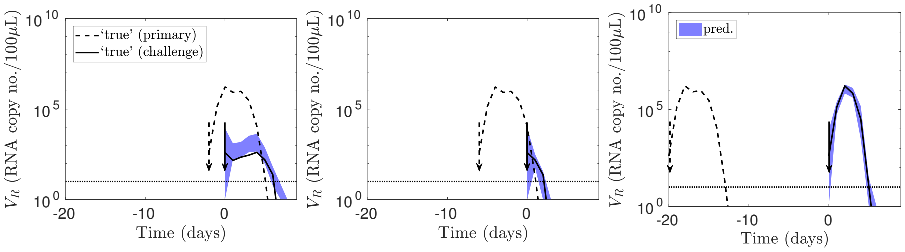

(i) 2-day interval (ii) 6-day interval (iii) 20-day interval

Figure D: **Predicting the outcomes of further sequential infection experiments.** The lines show the simulated 'true' viral loads for inter-exposure intervals of (i) 2, (ii) 6 and (iii) 20 days. The shaded areas show the 95% prediction intervals for the challenge viral load.

Each immune component's contribution to cross-protection

Having accurately recovered the timing and extent of cross-protection between strains, we then asked whether such cross-protection could be attributed to the 'correct' mechanisms (the same mechanisms as given by the 'true' parameters). These mechanisms are

- target cell depletion due to the infection and subsequent death of cells;
- innate immunity; and
- cellular adaptive immunity.

Before analysing the behaviour of the fitted models, we quantified how each immune component contributed to cross-protection for the 'true' parameters. In Fig. E, for a one, seven, or 14-day inter-exposure interval, we plotted in red the challenge viral load for the baseline model (the original model fitted to the data, where all three of the above immune components could mediate cross-protection). We observed that for a one-day inter-exposure interval, the viral load was initially suppressed relative to a single infection. For a seven-day inter-exposure interval, the challenge infection was prevented, while for a 14-day inter-exposure interval, the challenge infection was shortened.

We then modified the baseline model such that only a subset of immune components mediate cross-protection, as detailed in the Materials and Methods section. We used the modified model to predict the viral load (in black), and compared it with the baseline viral load.

For example, in Fig. Ei, for a one-day inter-exposure interval, we modified the baseline model such that only cellular adaptive immunity, and not target cell depletion or innate immunity, can mediate cross-protection. We denoted this modified model 'model XC'. Unlike the baseline model (red dotted line), the challenge viral load for model XC was not delayed (black solid line); in fact, it closely resembled that for a single infection. Comparing the two simulations

led to the conclusion that cellular adaptive immunity did not play a major part in cross-protection for a one-day inter-exposure interval.

We then modified the baseline model such that both target cell depletion and innate immunity can mediate cross-protection, but cellular adaptive immunity cannot do so. We denoted this model 'model XIT'. The challenge viral loads according to model XIT and the baseline model were similarly delayed (Fig. Eii). Hence, for the 'true' parameters, cross-protection was mediated by innate immunity and/or target cell depletion. The deviation between the baseline model and model XIT indicates that when the challenge infection is delayed, cellular adaptive immunity mediated timely resolution of infection.

To distinguish between these two mechanisms, we constructed model XI, where only innate immunity, and not target cell depletion or cellular adaptive immunity, can mediate cross-protection. The challenge viral load was very similar between model XI and model XIT (Fig. Eiii). We also constructed model XT, where only target cell depletion, and not innate immunity or cellular adaptive immunity, can mediate cross-protection. The challenge viral load for model XT was not delayed, and resembled that for a single infection (Fig. Eiv). We concluded that the cross-protection was largely mediated by innate immunity.

For seven and 14-day inter-exposure intervals, only cellular adaptive immunity mediated cross-protection (either preventing or shortening the second infection). This is evidenced by the protection observed for model XC (Fig. Ev, ix) but not models XIT, XI or XT (Fig. Evi–viii, x–xii).

We then sampled parameter sets from the joint posterior distributions obtained by fitting the baseline model to sequential infection data, and used them as inputs for models XC, XIT, XI and XT respectively, to generate the blue areas in Fig. E. If the modified models made the same predictions using the fitted parameters and the 'true' parameters, then the fitted model attributed cross-protection to the 'correct' mechanisms.

In each scenario, the model fitted to sequential infection, high cross-reactivity data accurately predicted challenge outcomes (shaded areas). This result demonstrates that the fitted model captures the cross-protection conferred by cellular adaptive immunity, target cell depletion, and innate immunity.

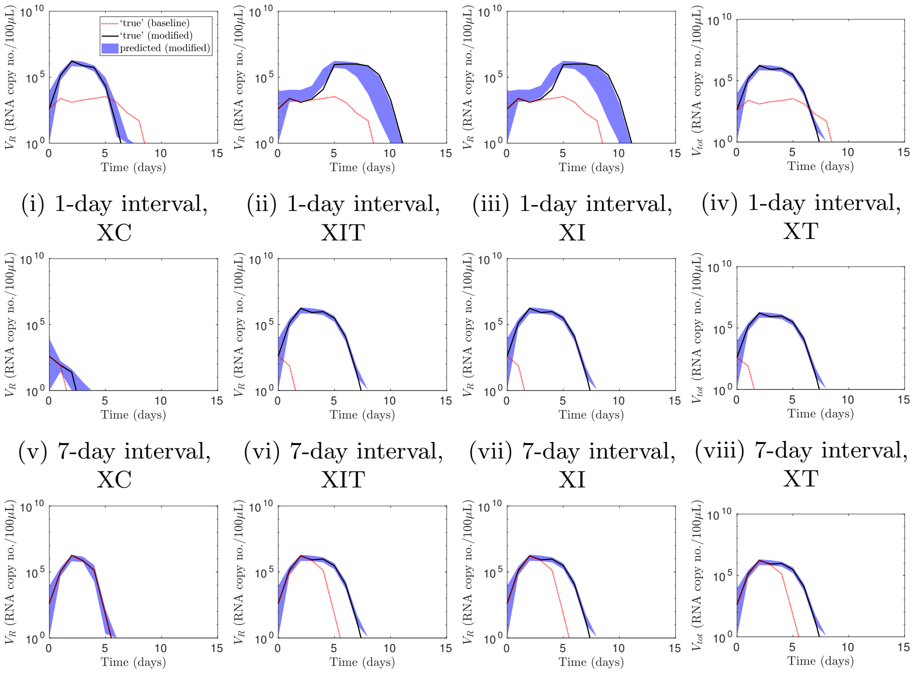

(i) 1-day interval, XC (ii) 1-day interval, XIT (iii) 1-day interval, XI (iv) 1-day interval, XT

(v) 7-day interval, XC (vi) 7-day interval, XIT (vii) 7-day interval, XI (viii) 7-day interval, XT

(ix) 14-day interval, XC (x) 14-day interval, XIT (xi) 14-day interval, XI (xii) 14-day interval, XT

Figure E: **Predictions of the challenge viral load when the mechanisms mediating cross-protection were restricted.** The challenge viral load for the 'true' parameter values when the mechanisms mediating cross-protection were restricted (models XC, XIT, XI and XT, black solid lines) is compared to the viral load for the baseline model (red dotted lines). For a one-day inter-exposure interval, innate immunity delayed the second infection, whereas cellular adaptive immunity was responsible for timely resolution of the infection. For seven-day and 14-day inter-exposure intervals, cellular adaptive immunity mediated cross-protection. The fitted model accurately predicted the challenge outcomes (95% prediction intervals shaded).

References

1. Laurie KL, Guarnaccia TA, Carolan LA, Yan AWC, Aban M, Petrie S, et al. Interval between infections and viral hierarchy are determinants of viral interference following influenza virus infection in a ferret model. J Infect Dis. 2015;212(11):1701–1710. doi:10.1093/infdis/jiv260.
