# Supplementary material for: Sequential infection experiments for quantifying innate and adaptive immunity during influenza infection
Source: PLoS Comput Biol. 2019 Jan 17;15(1):e1006568. doi: 10.1371/journal.pcbi.1006568 (PMC6353225; doi:10.1371/journal.pcbi.1006568)
Supplement: S3 File — (PDF) [file pcbi.1006568.s009.pdf]

Sequential infection experiments for quantifying innate and adaptive immunity during influenza infection

File S3:
Convergence diagnostics for the MCMC chains

Figures Ai–ii show trace plots for the log likelihood of an MCMC chain starting at a random point in parameter space for the single infection dataset. Figure Ai shows the first 100 iterations during the calibration period; the likelihood of the random parameter values at the start of the chain was much lower than that of the values used to generate the synthetic data (horizontal line), but the likelihood quickly increased with the number of iterations. Figure Aii shows the iterations after burn-in; the log likelihood fluctuated about its value for the parameters used to generate the synthetic data. (Because noise was added to the simulated data, we did not expect the true parameters to have the maximum likelihood.) This suggests that convergence has occurred. Figures Aiii–iv show similar results for the sequential infection dataset.

Tables A and B show the potential scale reduction factor and effective sample size for each of the parameters for the single infection and sequential infection dataset, after the burn-in iterations were discarded. The potential scale reduction factors were below 1.1 for every parameter, suggesting convergence; the effective sample size for each parameter was also large.

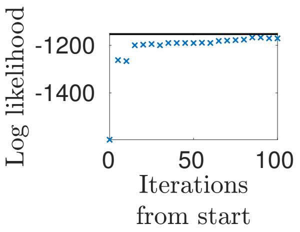

(i) single infection:
initial iterations

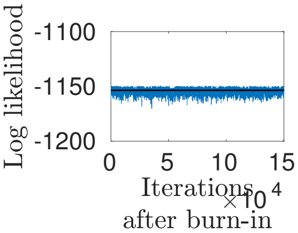

(ii) single infection:
after burn-in

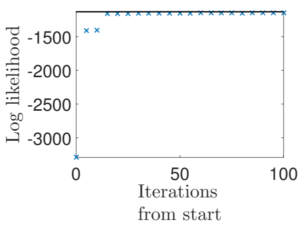

(iii) sequential infection:
initial iterations

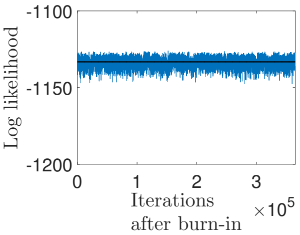

(iv) sequential infection:
after burn-in

Figure A: Log likelihood trace plots for the fits to the (top) single infection and (bottom) sequential infection datasets. (Left) the first 100 iterations during the calibration period; (right) the iterations after burn-in. The horizontal line shows the log likelihood for the parameter values used to generate the data. The log likelihood is plotted every 5 iterations.

	Point est.	Upper C.I.	Effective sample size
$\log_{10} g$	1.00	1.00	51570.28
$\log_{10} \delta_F$	1.00	1.01	4461.84
$\log_{10} \phi$	1.00	1.01	7721.11
$\log_{10} T_0$	1.00	1.01	18165.31
$\log_{10} \rho$	1.00	1.00	14156.31
$\log_{10} \alpha$	1.01	1.02	3290.13
$\log_{10} R_0$	1.02	1.07	1825.87
$\log_{10} r$	1.01	1.02	2102.56
$\log_{10} \delta_I$	1.01	1.03	2120.19
$\log_{10}(\delta_{Vinf} - \delta_{Vtot})$	1.00	1.00	12768.72
$\log_{10} \delta_{Vtot}$	1.01	1.03	3304.28
$\log_{10} \kappa_F$	1.01	1.02	3441.87
$\log_{10} s$	1.02	1.05	4609.35
$\log_{10} \kappa_A$	1.02	1.07	7376.80
$\log_{10} \delta_A$	1.00	1.00	112908.19
$\log_{10} \delta_B$	1.00	1.01	16218.57
$\log_{10} k_B$	1.00	1.01	26469.95
$\log_{10} \beta_B$	1.00	1.01	19694.83
$\log_{10} \tau_B$	1.01	1.03	15776.17
$\log_{10} p_{Vratio}$	1.02	1.07	2488.58
$\log_{10} \gamma$	1.00	1.00	196077.05
$\log_{10} V_0$	1.00	1.00	6059.54
$\log_{10} \beta_C$	1.00	1.01	2880.26
$\log_{10} \delta_E$	1.01	1.03	2721.70
$\log_{10} \tau_M$	1.00	1.00	80758.58
$\log_{10} \tau_E$	1.01	1.02	2439.96
$\log_{10} \epsilon$	1.00	1.00	59634.66
$\log_{10} k_{C11}$	1.01	1.03	2967.73
$\log_{10} k_{C12}$	1.00	1.00	4373.99
$\log_{10} \kappa_{E11}$	1.01	1.03	2147.61
σ	1.00	1.00	92620.87

Table A: Potential scale reduction factor (point estimate and upper bound of 95% confidence interval) and effective sample size for each parameter, for the model fitted to the single infection dataset. The first 111,000 iterations of each chain are discarded, leaving 290,001 samples from each chain and 570,003 samples across the three chains.

	Point est.	Upper C.I.	Effective sample size
$\log_{10} g$	1.00	1.00	28609.24
$\log_{10} \delta_F$	1.00	1.01	3223.48
$\log_{10} \phi$	1.00	1.01	9489.14
$\log_{10} T_0$	1.00	1.00	39185.98
$\log_{10} \rho$	1.00	1.00	25699.60
$\log_{10} \alpha$	1.01	1.02	1478.19
$\log_{10} R_0$	1.01	1.02	1475.90
$\log_{10} r$	1.01	1.01	2347.94
$\log_{10} \delta_I$	1.01	1.01	1439.54
$\log_{10}(\delta_{Vinf} - \delta_{Vtot})$	1.01	1.05	6465.66
$\log_{10} \delta_{Vtot}$	1.03	1.07	1969.53
$\log_{10} \kappa_F$	1.00	1.01	4532.63
$\log_{10} s$	1.00	1.00	2527.72
$\log_{10} \kappa_A$	1.01	1.02	2285.39
$\log_{10} \delta_A$	1.00	1.00	95220.75
$\log_{10} \delta_B$	1.01	1.03	3047.21
$\log_{10} k_B$	1.01	1.03	3964.78
$\log_{10} \beta_B$	1.01	1.03	3503.46
$\log_{10} \tau_B$	1.03	1.10	3762.41
$\log_{10} p_{Vratio}$	1.03	1.06	1229.04
$\log_{10} \gamma$	1.00	1.00	164376.88
$\log_{10} V_0$	1.02	1.06	1636.96
$\log_{10} \beta_C$	1.01	1.02	6123.05
$\log_{10} \delta_E$	1.01	1.03	5036.48
$\log_{10} \tau_M$	1.00	1.00	170820.42
$\log_{10} \tau_E$	1.01	1.02	4979.02
$\log_{10} \epsilon$	1.00	1.00	161894.61
$\log_{10} k_{C11}$	1.01	1.03	9151.38
$\log_{10} k_{C12}$	1.01	1.04	14663.48
$\log_{10} \kappa_{E11}$	1.03	1.08	2291.62
σ	1.00	1.00	44387.40

Table B: Potential scale reduction factor (point estimate and upper bound of 95% confidence interval) and effective sample size for each parameter, for the model fitted to the sequential infection dataset. The first 35,000 iterations of each chain are discarded, leaving 165,001 samples from each chain and 495,003 samples across the three chains.
