# Supplementary material for: Sequential infection experiments for quantifying innate and adaptive immunity during influenza infection
Source: PLoS Comput Biol. 2019 Jan 17;15(1):e1006568. doi: 10.1371/journal.pcbi.1006568 (PMC6353225; doi:10.1371/journal.pcbi.1006568)
Supplement: S4 File — (PDF) [file pcbi.1006568.s010.pdf]

Sequential infection experiments for quantifying innate and adaptive immunity during influenza infection

File S4:
Marginal posterior distributions for the model parameters

This document shows histograms of samples from the marginal posterior distributions of each parameter. We emphasise that the aim of the study was not to estimate model parameters, but to quantify the roles of each immune component in controlling infection. Nonetheless, the marginal posterior distributions provide a way to gauge the additional information provided by sequential infection experiments over single infection experiments.

Furthermore, if the parameter values used to generate the synthetic data can be re-estimated, then fitting the model to real experimental data will reveal information about the rates at which processes occur in the system, which may be useful when determining reasonable parameter values for future models. On the other hand, if the marginal posterior distributions do not reflect the true parameter values, this cautions against relying on them for accurate parameter estimates.

Because of the large number of parameters, particular parameters are selected as examples of qualitative behaviours observed; the full set of marginal posterior distributions is presented in Figs. E–H later in this document.

Some parameters were estimated accurately by the fitted models. Figure A shows two examples of these parameters: the initial viral load growth rate r and the total viral decay rate δ_{Vtot}. To assess whether the data are informative, the marginal posterior distributions were compared to the prior distributions (leftmost column). The prior distributions were obtained by sampling from the joint prior distribution using the Metropolis algorithm as outlined in the main text, but setting $P(\mathbf{y}|\boldsymbol{\theta})$ to a constant.

The remaining columns show the marginal posterior distributions of the same parameter for the model fitted to the single infection and sequential infection datasets. The parameter values used to generate the datasets are marked by the vertical line.

The marginal posterior distributions indicate that the value of r was accurately estimated from each of the datasets, which was perhaps unsurprising because r is the initial slope of the viral load trajectory. However, the model fitted to the sequential infection data (Fig. Aiii) estimated r much more precisely. In particular, the model fitted to single infection data only (Fig. Aii) could not

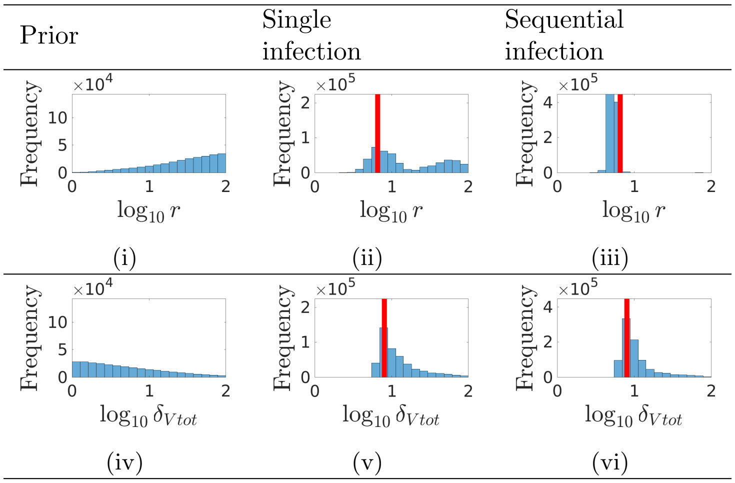

Figure A: *Some parameters were recovered accurately by fitting to sequential infection data.* Leftmost column: the marginal prior distribution of the parameter in the horizontal axis label. Remaining columns: the marginal posterior distributions of the same parameter for the model fitted to the dataset indicated. The parameter value used to generate the data is marked by the vertical line.

exclude the possibility that r was orders of magnitude larger than its true value. For the predicted viral load trajectory to fit the data given these large values of r, its growth rate must have slowed down very early on due to target cell depletion or innate immunity. The model fitted to sequential infection data was able to exclude this possibility.

Similarly, the value of the total viral load decay rate, δ_{Vtot}, was estimated accurately from the data. The accurate recovery of this parameter value was because in the absence of infected cells and infectious virions, the total viral load decayed at rate δ_{Vtot}, so the steepest negative gradient of the viral load curve during the resolution phase (on a log scale) provided a lower bound for δ_{Vtot}.

In contrast, Figs. Bi–iii show that the marginal posterior distributions for the antibody decay rate, δ_A, were the same as the marginal prior distribution. This non-identifiability occurred because the continued growth of antibodies after the end of a primary infection did not impact its viral load, and also did not impact a second infection due to the lack of cross-reactivity in the antibody response.

However, even if the marginal posterior distribution remained unchanged from the prior distribution, such that a parameter was not practically identifiable by itself, the data may contain information about a combination of parameters. Figures Biv–vi show that the marginal posterior distributions for the B cell decay rate, δ_B, were also the same as the prior distribution. However, when the value of δ_B was changed while holding all other parameters constant, the

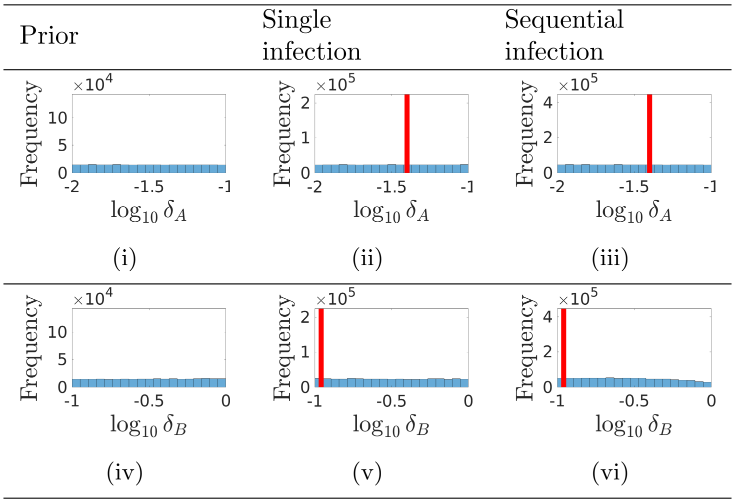

Figure B: *Some parameters were poorly estimated by fitting to sequential infection data.* Leftmost column: the marginal prior distribution of the parameter in the horizontal axis label. Remaining columns: the marginal posterior distributions of the same parameter for the model fitted to the dataset indicated. The parameter value used to generate the data is marked by the vertical line.

likelihood changed drastically (Fig. Cii), whereas there was no such change for δ_A (Fig. Ci). Hence, one could not determine the identifiability of a parameter in combination with others by examining the marginal posterior distributions alone.

To better quantify the practical identifiability of each parameter, one could use the profile likelihood method [1]. For each parameter, as the value of the parameter is changed, the likelihood over all remaining parameters would be maximised. The maximised likelihood would only change if the target parameter can be identified from the data independently from all others. However, maximising over the large number of parameters in this model is extremely computationally costly.

Despite the lack of information in the marginal posterior distribution of some parameters (Fig. B as well as many parameters in Figs. E–H), the model fitted the viral load data well, as shown in the main text. As the estimation of parameter values was not our ultimate goal, rather than relying on the marginal posterior distributions only, we used prediction methods in the main text to directly investigate how each model quantitatively attributed control of an infection to each immune component.

Further reason to use prediction methods, rather than only the marginal posterior distributions, was the apparent bias in the marginal posterior distributions for some parameters. Figure D shows that the marginal posterior distributions for the basic reproduction number R_0 were biased towards much lower values than the value used to construct the synthetic datasets.

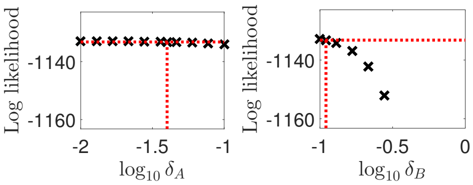

(i) (ii)

Figure C: *The log likelihood remained roughly constant as the value of δ_A was changed in isolation, but changed drastically when δ_B was changed.* The log likelihood for the sequential infection dataset given the model parameters as (i) δ_A and (ii) δ_B were varied while the other parameters were kept at the values used to generate the synthetic data. The vertical line indicates the true parameter value, and the horizontal line indicates the log likelihood of the data given the true set of parameter values.

The discrepancy between the value of R_0 used to generate the synthetic data and the estimated values is of interest because previous studies have shown that R_0 can be written as a function of the initial viral growth rate r, the infectious cell decay rate δ_I and the infectious virion decay rate δ_V [2] — each of which are estimated in an unbiased manner (Fig. E). Moreover, in the simplest population-scale equivalent of a viral dynamics model — the SIR model — R_0 can be inferred reliably from data [3].

We hypothesise that the estimated value of R_0 has a lesser effect in our model because clearance is driven by the immune response. By contrast, in a viral dynamics model without a time-dependent immune response, clearance is driven by target cell depletion. Similarly, in the SIR model, the epidemic ends through depletion of susceptibles. This difference between the effects of R_0 in different models arises because in the models where resolution is driven by depletion of susceptibles (cells/individuals), the decay rate towards the end of the infection curve is either the removal rate of infectious individuals (SIR model) or a function of the non-specific decay rates of virions and infected cells (δ_I and δ_V). These quantities appear in R_0. On the other hand, if resolution is driven by the immune response, the decay rate at the end of the infection is a combination of adaptive immune parameters and non-specific decay of infected cells and virions, the former of which do not appear in R_0. Hence, although the underlying reason for the bias in the marginal posterior distribution of R_0 remains to be investigated, this parameter has less influence on the viral load than may be expected, and the viral load is nonetheless recovered accurately. This example again highlights the caution required when interpreting marginal posterior distributions.

Figures E–H show the posterior distributions for the full set of parameters.

Prior	Single infection	Sequential infection

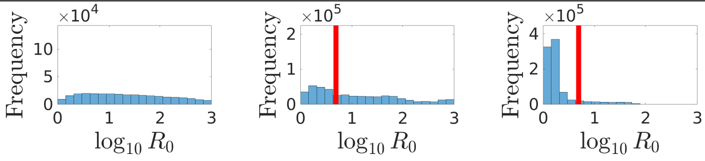

(i) (ii) (iii)

Figure D: *The marginal posterior distributions for R_0 were biased towards low values.* Leftmost column: the marginal prior distribution of the parameter in the horizontal axis label. Remaining columns: the marginal posterior distributions of the same parameter for the model fitted to the dataset indicated. The parameter value used to generate the data is marked by the vertical line.

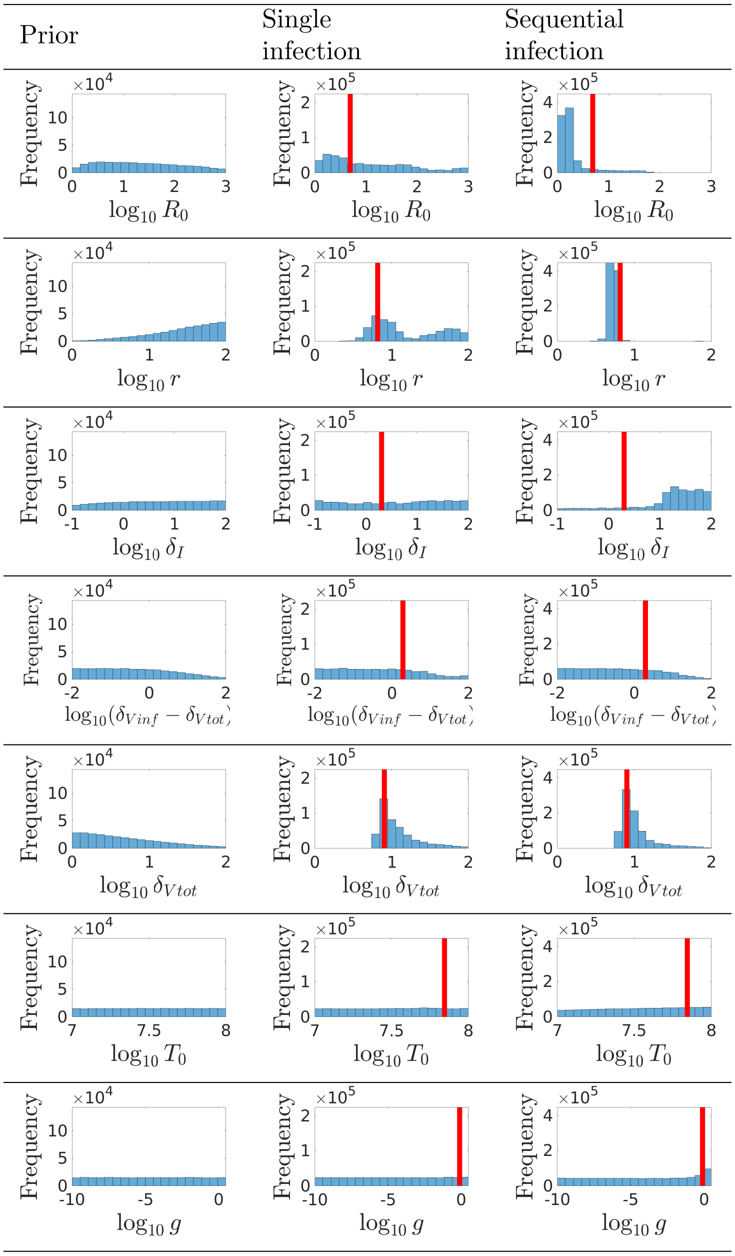

Figure E: The full set of marginal posterior distributions. Leftmost column: the marginal prior distribution of the parameter in the horizontal axis label. Remaining columns: the marginal posterior distributions of the same parameter for the model fitted to the dataset indicated. The parameter value used to generate the data is marked by the vertical line.

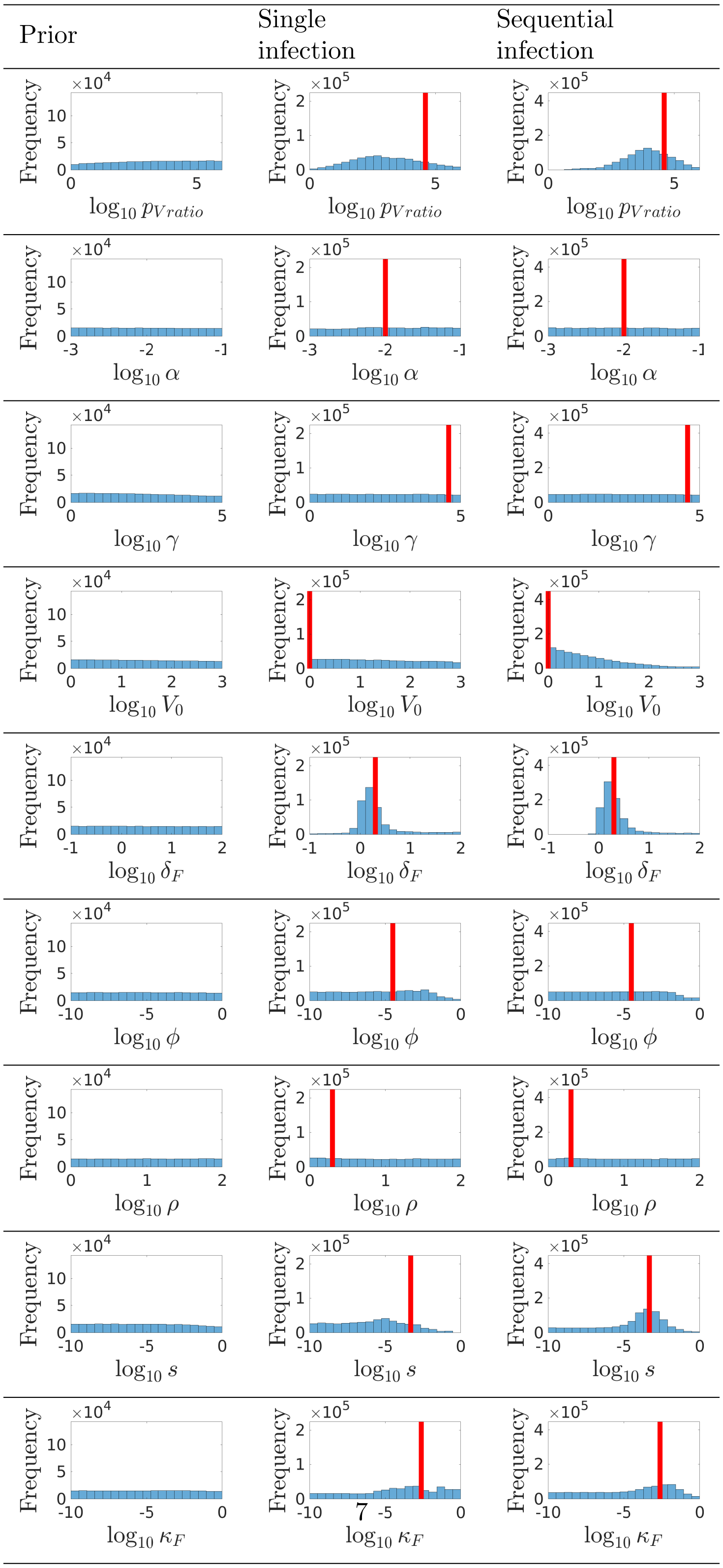

Figure F: Marginal distributions (continued from Fig. E).

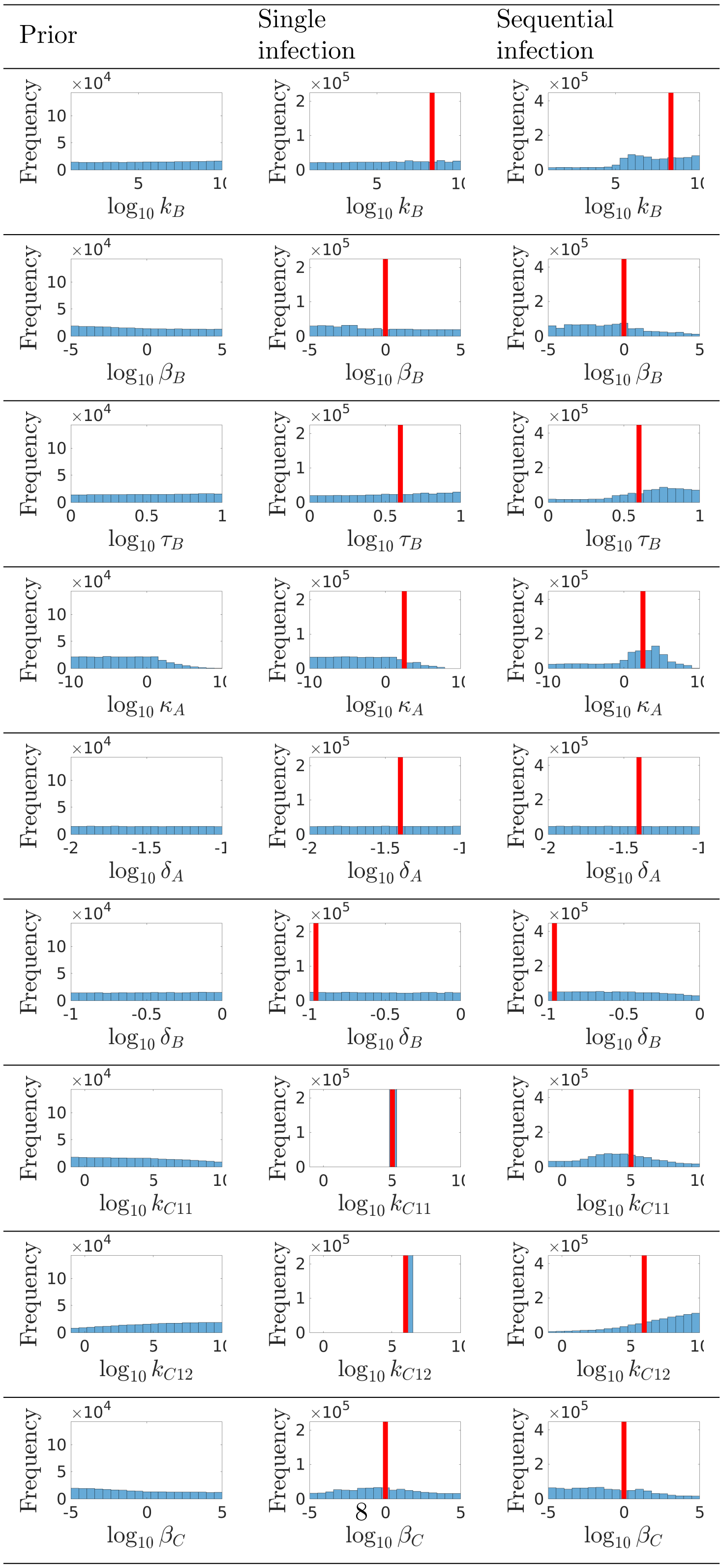

Figure G: Marginal distributions (continued from Fig. F).

Prior	Single infection	Sequential infection

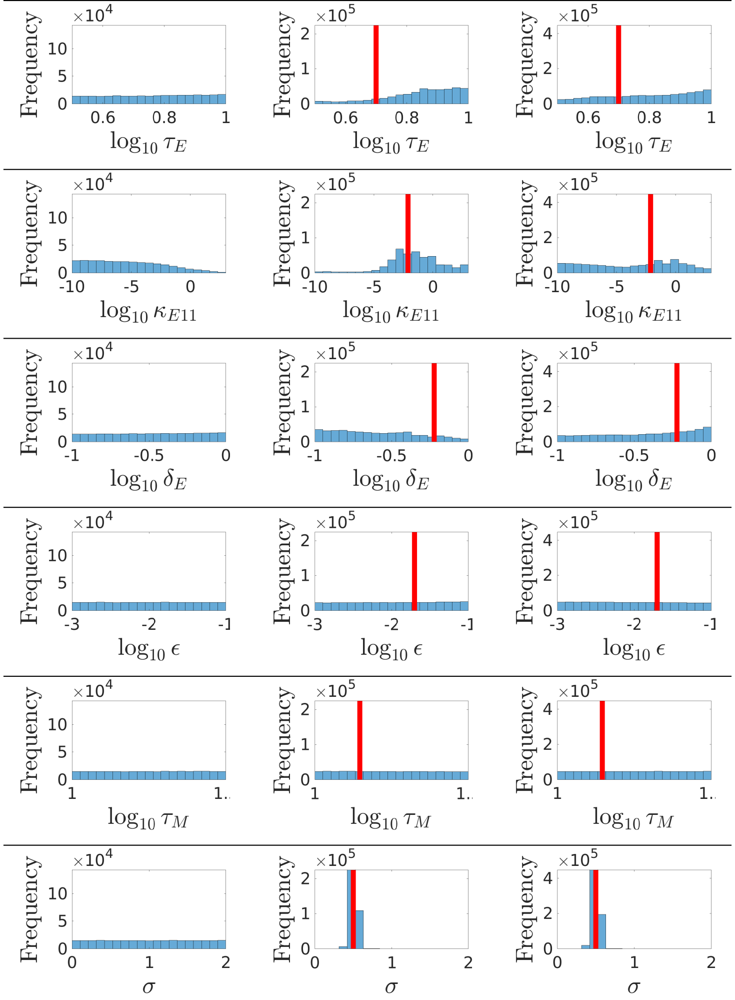

Figure H: Marginal distributions (continued from Fig. G).

References

1. Raue A, Kreutz C, Maiwald T, Bachmann J, Schilling M, Klingmüller U, et al. Structural and practical identifiability analysis of partially observed dynamical models by exploiting the profile likelihood. Bioinformatics. 2009;25(15):1923–1929. doi:10.1093/bioinformatics/btp358.

2. Nowak MA, Lloyd AL, Vasquez GM, Wiltrout TA, Wahl LM, Bischofberger N, et al. Viral dynamics of primary viremia and antiretroviral therapy in simian immunodeficiency virus infection. J Virol. 1997;71(10):7518–25.

3. Heffernan JM, Smith RJ, Wahl LM. Perspectives on the basic reproductive ratio. J R Soc Interface. 2005;2(4):281–293. doi:10.1098/rsif.2005.0042.
