# Supplementary material for: Sequential infection experiments for quantifying innate and adaptive immunity during influenza infection
Source: PLoS Comput Biol. 2019 Jan 17;15(1):e1006568. doi: 10.1371/journal.pcbi.1006568 (PMC6353225; doi:10.1371/journal.pcbi.1006568)
Supplement: S5 File — (PDF) [file pcbi.1006568.s011.pdf]

Sequential infection experiments for quantifying innate and adaptive immunity during influenza infection

File S5:
Results for a different set of 'true' parameters

Parameter selection

The aim of this document is to test whether the findings in the main text are robust to the choice of 'true' parameters. To facilitate this, a new set of parameters for the model in the main text was chosen to generate the qualitative behaviour observed in the study by Laurie *et al.* [1] for infection with influenza A followed by influenza B, or vice versa. Because of the non-linearities present in the model, it is difficult to manually choose a completely new set of parameters to reproduce the same qualitative behaviour. In File S6, we tested the robustness of the results in the main study to model misspecification, by using a different model to generate data reproducing the desired qualitative behaviour, then fitting our model to the new data. As parameter sets from the joint posterior distribution in File S6 produce the required behaviour, we chose one of these parameter sets at random for use in this document.

The new parameters are given in Tables A–D. When compared to the parameters in the main text, these parameters represent a stronger cellular adaptive immune response, and weaker innate and humoral adaptive immune responses. Synthetic data was generated using these parameters and measurement error distributed with standard deviation $\sigma = 0.5$.

Figure A shows a subset of the synthetic data.

Parameter	Value	Units
$\log_{10} R_0$	1.8866	
$\log_{10} r$	1.0320	day^{-1}
$\log_{10} \delta_I$	-0.4450	day^{-1}
$\log_{10}(\delta_{Vinf} - \delta_{Vtot})$	-0.5094	day^{-1}
$\log_{10} \delta_{Vtot}$	0.4444	day^{-1}
$\log_{10} T_0$	7.3888	target cell
$\log_{10} g$	-0.6192	day^{-1}
$\log_{10} p_{Vratio}$	0.5570	
$\log_{10} \alpha$	-1.5403	RNA copies/100μL $virion^{-1}$
$\log_{10} \gamma$	0.4388	
$\log_{10} V_{inf0}$	1.5195	virion

Table A: Viral replication parameter values.

Parameter	Value	Units
$\log_{10} \delta_F$	1.7625	day^{-1}
$\log_{10} \phi$	-6.0012	day^{-1}
$\log_{10} \rho$	1.1965	day^{-1}
$\log_{10} s$	-4.7167	day^{-1}
$\log_{10} \kappa_F$	-3.2587	day^{-1}

Table B: Innate immune response parameter values.

Experiment	Parameter	Value	Units
Single infection	$\log_{10} k_{C11}$	5.2430	infected cell
	$\log_{10} k_{C21}$	8.7840	infected cell
	$\log_{10} \kappa_{E11}$	-0.2997	day^{-1}
Sequential infection	$\log_{10} k_{C11} = \log_{10} k_{C22}$	5.2430	infected cell
	$k_{C12} = k_{C21}$	∞	infected cell
	$\log_{10} k_{C31} = \log_{10} k_{C32}$	8.7840	infected cell
	$\log_{10} \kappa_{E11}$	-0.2997	day^{-1}

Table C: Values for the cross-reactivity parameters in the cellular adaptive immune response.

Parameter	Value	Units
$\log_{10} k_B$	5.8616	RNA copy no./100μL
$\log_{10} \beta_B$	-1.9355	day^{-1}
$\log_{10} \tau_B$	0.0107	day
$\log_{10} \kappa_A$	-2.6421	day^{-1}
$\log_{10} \delta_A$	-1.4795	day^{-1}
$\log_{10} \delta_B$	-0.7007	day^{-1}
$\log_{10} \beta_C$	-0.4627	day^{-1}
$\log_{10} \tau_E$	0.8960	day
$\log_{10} \delta_E$	-0.5481	day^{-1}
$\log_{10} \epsilon$	-2.3938	
$\log_{10} \tau_M$	1.4735	day

Table D: Adaptive immune response parameter values.

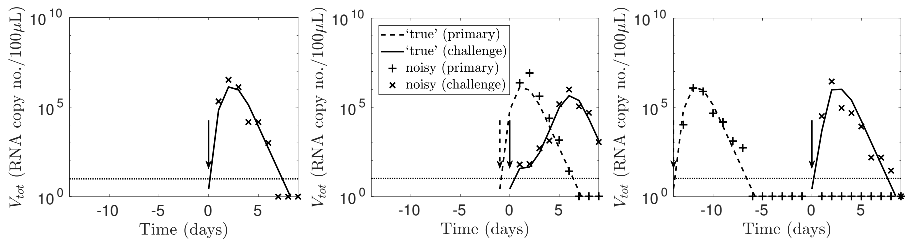

(i) single infection (ii) 1-day interval (iii) 14-day interval

Figure A: **A subset of the synthetic data.** (i) The line shows the simulated 'true' viral load for a single infection, with the arrow showing the time of exposure. The simulated viral load with noise is shown as crosses. The horizontal line indicates the observation threshold (10 RNA copy no./100μL); observations below this threshold are plotted below this line. Values below the observation threshold were treated as censored. (ii–iii) For sequential infections with the labelled inter-exposure interval, the dashed and dotted lines show the simulated 'true' viral load for a primary and challenge infection respectively; the arrows show the times of the primary and challenge exposures. The simulated viral load with noise is shown as crosses.

Results

Verification of the fitting procedure

Paralleling the main text, we first verified that our model fitting procedure recovers the simulated 'true' viral load.

Figure B presents 95% credible intervals for the viral load. The credible intervals included the 'true' viral load, confirming accurate recovery.

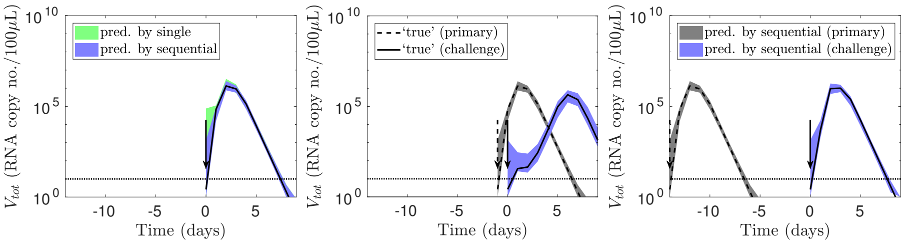

(i) single infection (ii) 1-day interval (iii) 14-day interval

Figure B: **Verification that the fitting procedure recovered the viral load.** (i) For a single infection, the blue and green areas are the 95% credible intervals for the viral load (in the absence of noise), as predicted by the models fitted to the sequential infection and single infection data respectively. (ii–iii) For sequential infections with the labelled inter-exposure interval, the grey and blue areas show the 95% credible intervals for the primary and challenge viral load respectively, predicted by the model fitted to sequential infection data. The other elements of the figure are identical to Fig 1 in the main text: the dashed and dotted lines show the simulated 'true' viral load for a primary and challenge infection respectively; the arrows show the times of the primary and challenge exposures; and the horizontal line indicates the observation threshold.

Comparing the immunological information in each dataset

Next, we compared the behaviour of the fitted models to the behaviour of the 'true' parameters, to determine the information in each dataset on

- the effect of each immune component in controlling a single infection;
- cross-protection between strains; and
- each immune component's contribution to cross-protection.

The effect of each immune component in controlling a single infection

In Fig. C, we removed various immune components from the model. Similar to the main text, removing adaptive immunity caused infection to become chronic (Fig. Ci); removing innate immunity increased the peak viral load (Fig. Cii); and removing innate and adaptive immunity increased the peak viral load and delayed resolution of the infection (Fig. Ciii). However, unlike the parameters in the main text, removing humoral adaptive immunity had no effect (Fig. Civ), and removing cellular adaptive immunity caused the infection to become chronic (Fig. Cv).

We then compared predictions of the viral load for a single infection by the models fitted to the two datasets. The model fitted to sequential infection data was able to estimate the timing of immune components. For the fitted model, the viral load without adaptive immunity deviated from the baseline at three days post-infection, and the viral load without innate immunity deviated from the baseline one day post-infection (Figs. Ci–ii). These timings were the same as for the 'true' parameters. However, unlike in the main text, the fitted model was unable to predict the viral load in the absence of adaptive immunity, as shown by the wide credible interval in Fig. Ci. A possible explanation for the different result is that for the parameters in the main text, the viral load showed a clear plateau while innate but not adaptive immunity was active, enabling the fitted model to predict that the viral load would stay at that plateau in the absence of adaptive immunity. By contrast, the viral load in Fig. A in this text did not show a clear plateau during the innate immunity phase, possibly reducing the fitted model's ability to infer the viral load in the absence of adaptive immunity. The model fitted to sequential infection data was also unable to predict the viral load in the absence of innate immunity, or distinguish between humoral and cellular adaptive immunity (Figs. Cii–v). These results are consistent with the main text. Compared to sequential infection data, single infection data contains less information on the timing of immune components, which is also consistent with the main text. However, in the main text, the timing of adaptive immunity and not innate immunity was inferred, while the opposite holds for the data in this text. The inability of the model fitted to this single infection data set to infer the timing of adaptive immunity could be again due to the lack to a clear plateau in the viral load, the end of which signals a rise in adaptive immunity. On the other hand, the weaker innate immunity in this single infection data

set gave limited scope for the viral load in the absence of innate immunity to deviate from the baseline viral load, possibly aiding inference of the timing of innate immunity using the single infection data set. Also consistent with the main text, the credible intervals for the viral load predicted by the model fitted to single infection data were wider than those predicted by the model fitted to sequential infection data, regardless of the immune component(s) removed.

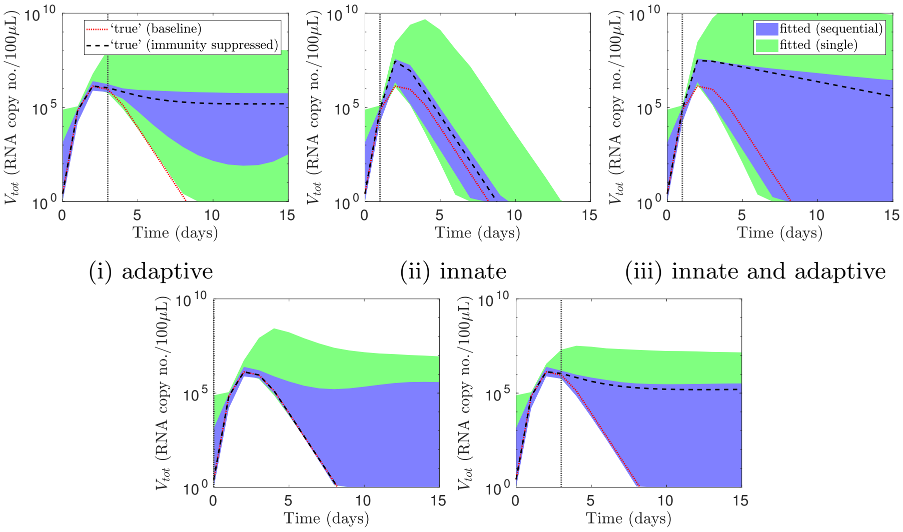

(i) adaptive (ii) innate (iii) innate and adaptive

(iv) humoral adaptive (v) cellular adaptive

Figure C: **Predicting the viral load for a single infection when various immune components were absent.** The vertical lines indicate, for the 'true' parameter values, the times at which the immune components labelled under each panel took effect. (This line is not plotted for humoral adaptive immunity as removing it had no effect.) These times were determined by when the viral load for the baseline model (red dotted line) deviated from the viral load when the immune components were absent (black dashed line). These times could be recovered using sequential infection data, but single infection data could only recover the timing of innate immunity. Credible intervals for the model fitted to sequential infection data were tighter than for the model fitted to single infection data. Prediction intervals were constructed without measurement noise.

Cross-protection between strains

Given the above mixed results, we then tested whether sequential infection data accurately captured the timing and extent of cross-protection, by simulating the viral load for inter-exposure intervals other than those where data was provided.

Figure D shows prediction intervals for the challenge viral load for inter-exposure intervals of (i) 2 and (ii) 20 days. Like in the main text, the blue areas,

which correspond to the model fitted to sequential infection data, accurately predict the viral load for the challenge strain. By contrast, the green areas, which correspond to the model fitted to single infection data, do not accurately predict the viral load for the challenge strain.

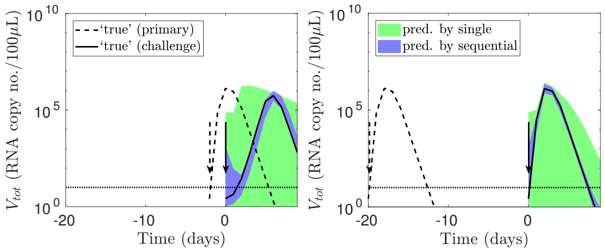

(i) 2-day interval (ii) 20-day interval

Figure D: **Predicting the outcomes of further sequential infection experiments.** Sequential infection data, but not single infection data, enabled prediction of further sequential infection experiment outcomes. The lines show the simulated 'true' viral loads for inter-exposure intervals of (i) 2 and (ii) 20 days. The shaded areas show the 95% prediction intervals for the challenge viral load.

Each immune component's contribution to cross-protection

Having accurately recovered the timing and extent of cross-protection between strains, we then asked whether such cross-protection could be attributed to the 'correct' mechanisms (the same mechanisms as given by the 'true' parameters). These mechanisms are

- target cell depletion due to the infection and subsequent death of cells;
- innate immunity; and
- cellular adaptive immunity.

Before analysing the behaviour of the fitted models, we quantified how each immune component contributed to cross-protection for the 'true' parameters. In Fig. E, for a one-day inter-exposure interval, we plotted in red the challenge viral load for the baseline model (all three of the above immune components could mediate cross-protection). We observed that for a one-day inter-exposure interval, the challenge infection was delayed.

We then modified the baseline model such that only a subset of immune components mediated cross-protection. We used different modified models to predict the viral load (in black), and compared them with the baseline viral load.

In Fig. Ei, we modified the baseline model such that only cellular adaptive immunity, and not target cell depletion or innate immunity, can mediate cross-protection. We denoted this modified model 'model XC'. Unlike the baseline model (red dotted line), the challenge viral load for model XC was not delayed (black solid line); in fact, it closely resembled that for a single infection. Comparing the two simulations led to the conclusion that cellular adaptive immunity did not play a major part in cross-protection for a one-day inter-exposure interval.

We then modified the baseline model such that both target cell depletion and innate immunity can mediate cross-protection, but cellular adaptive immunity cannot do so. We denoted this model 'model XIT'. The challenge viral loads according to model XIT and the baseline model were similarly delayed (Fig. Eii). Hence, for the 'true' parameters, cross-protection was mediated by innate immunity and/or target cell depletion.

To distinguish between these two mechanisms, we constructed model XI, where only innate immunity, and not target cell depletion or cellular adaptive immunity, can mediate cross-protection. The challenge viral load for model XI was delayed compared to a primary infection, but less so than for model XIT (Fig. Eiii). We also constructed model XT, where only target cell depletion, and not innate immunity or cellular adaptive immunity, can mediate cross-protection. The challenge viral load for model XT was similar to that for a primary infection (Fig. Eiv). We concluded that the cross-protection was largely mediated by innate immunity, with some contribution by target cell depletion.

We then sampled parameter sets from the joint posterior distributions obtained by fitting the model in the main text to sequential infection data, and used them as inputs for models XC, XIT, XI and XT respectively, to generate the areas in Fig. E. If the modified models made the same predictions using the fitted parameters and the 'true' parameters, then the fitted model attributed cross-protection to the 'correct' mechanisms.

The results were similar to the main text. Models XC and XIT made the same predictions using the fitted parameters (shaded area) and the 'true' parameters (black line), so sequential infection data enabled us to accurately attribute cross-protection to target cell depletion and/or innate immunity, rather than cellular adaptive immunity (Fig. Ei–ii). On the other hand, the fitted parameters did not consistently predict the challenge outcome for model XI, although predictive performance was better for model XT (Figs. Eiii–iv). Hence, we could not use sequential infection data to consistently quantify the contributions of target cell depletion and innate immunity to cross-protection.

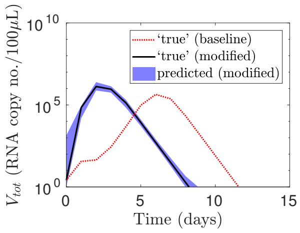

(i) XC

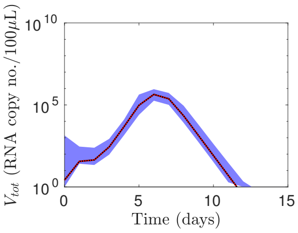

(ii) XIT

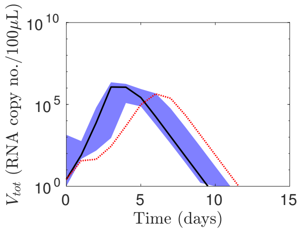

(iii) XI

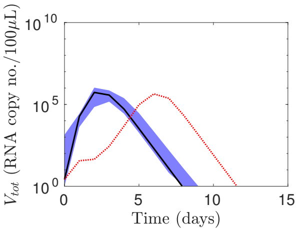

(iv) XT

Figure E: **Predictions of the challenge viral load for a one-day inter-exposure interval when the mechanisms mediating cross-protection were restricted.** The black solid lines show the challenge viral load for the 'true' parameter values when the mechanisms mediating cross-protection were restricted. The red dotted lines show the viral load for the baseline model. Comparing the two sets of lines reveals that target cell depletion and innate immunity mediated cross-protection, whereas cellular adaptive immunity did little to mediate cross-protection. The model fitted to sequential infection data accurately predicted the challenge outcomes for models XC, XIT and XT, but not model XI (95% prediction intervals shown). It thus correctly attributed cross-protection to target cell depletion and/or innate immunity, but could not definitively distinguish between the two. The viral load for the primary infection is not shown, to improve clarity of the figure.

References

1. Laurie KL, Guarnaccia TA, Carolan LA, Yan AWC, Aban M, Petrie S, et al. Interval between infections and viral hierarchy are determinants of viral interference following influenza virus infection in a ferret model. J Infect Dis. 2015;212(11):1701–1710. doi:10.1093/infdis/jiv260.
