# Supplementary material for: Sequential infection experiments for quantifying innate and adaptive immunity during influenza infection
Source: PLoS Comput Biol. 2019 Jan 17;15(1):e1006568. doi: 10.1371/journal.pcbi.1006568 (PMC6353225; doi:10.1371/journal.pcbi.1006568)
Supplement: S6 File — (PDF) [file pcbi.1006568.s012.pdf]

Sequential infection experiments for quantifying innate and adaptive immunity during influenza infection

File S6:
Results for different noisy datasets with the same 'true' parameters

We tested whether results would have changed had we generated the data in the main text using the same 'true' parameters but using a different random number generator seed, by generating two additional datasets with different random number generator seeds. Figure A shows a subset of this synthetic data.

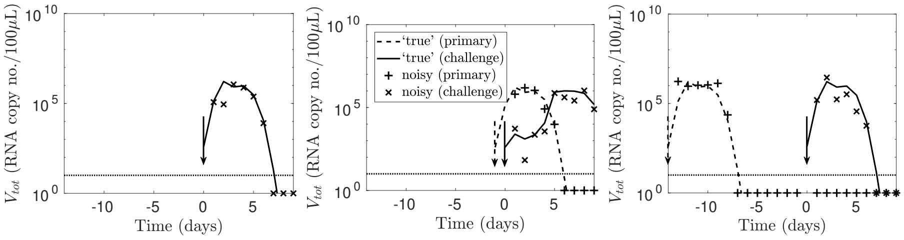

(i) single infection, dataset 2

(ii) 1-day interval, dataset 2

(iii) 14-day interval, dataset 2

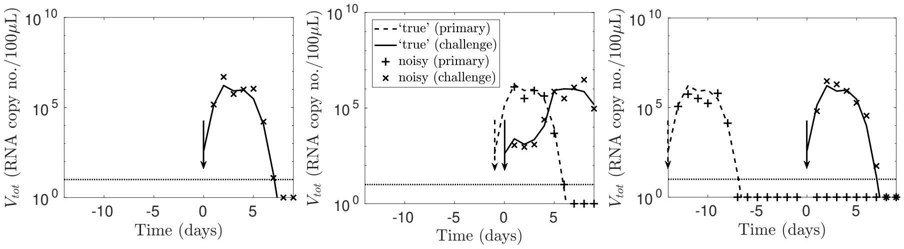

(iv) single infection, dataset 3

(v) 1-day interval, dataset 3

(vi) 14-day interval, dataset 3

Figure A: **A subset of the synthetic data.** (i, iv) The line shows the simulated 'true' viral load for a single infection, with the arrow showing the time of exposure. The simulated viral load with noise is shown as crosses. The horizontal line indicates the observation threshold (10 RNA copy no./100μL); observations below this threshold are plotted below this line. Values below the observation threshold were treated as censored. (ii–iii, v–vi) For sequential infections with the labelled inter-exposure interval, the dashed and dotted lines show the simulated 'true' viral load for a primary and challenge infection respectively; the arrows show the times of the primary and challenge exposures. The simulated viral load with noise is shown as crosses.

Results

Verification of the fitting procedure

Paralleling the main text, we first verified that our model fitting procedure recovers the simulated 'true' viral load.

Figure B presents 95% credible intervals for the viral load. The credible intervals included the 'true' viral load, confirming accurate recovery.

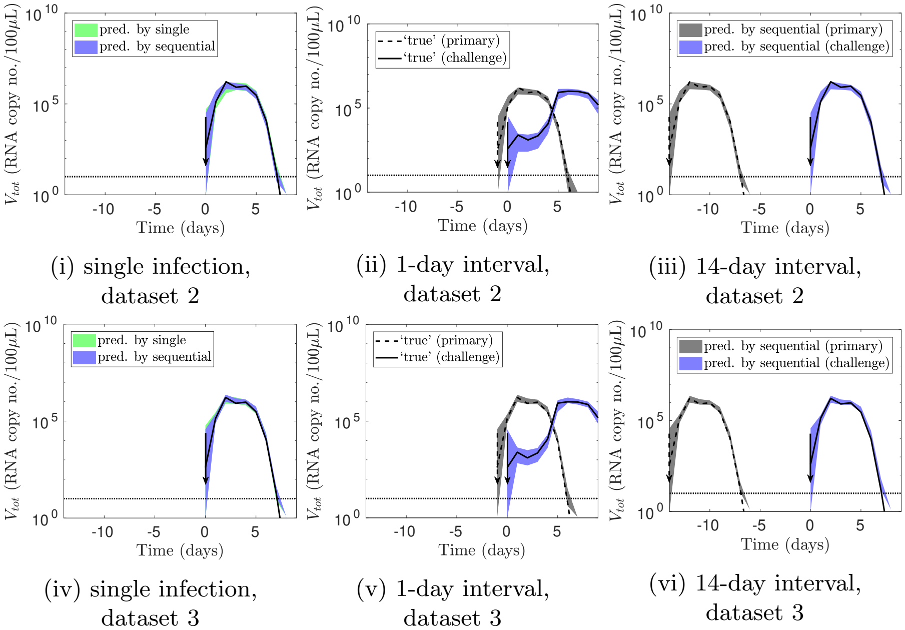

(i) single infection, dataset 2

(ii) 1-day interval, dataset 2

(iii) 14-day interval, dataset 2

(iv) single infection, dataset 3

(v) 1-day interval, dataset 3

(vi) 14-day interval, dataset 3

Figure B: **Verification that the fitting procedure recovered the viral load.** (i, iv) For a single infection, the blue and green areas are the 95% credible intervals for the viral load (in the absence of noise), as predicted by the models fitted to the sequential infection and single infection data respectively. (ii–iii, v–vi) For sequential infections with the labelled inter-exposure interval, the grey and blue areas show the 95% credible intervals for the primary and challenge viral load respectively, predicted by the model fitted to sequential infection data. The other elements of the figure are identical to Fig 1 in the main text: the dashed and dotted lines show the simulated 'true' viral load for a primary and challenge infection respectively; the arrows show the times of the primary and challenge exposures; and the horizontal line indicates the observation threshold.

Comparing the immunological information in each dataset

Next, we compared the behaviour of the fitted models to the behaviour of the 'true' parameters, to determine the information in each dataset on

- the effect of each immune component in controlling a single infection;
- cross-protection between strains; and
- each immune component's contribution to cross-protection.

The effect of each immune component in controlling a single infection

In Fig. C, we removed various immune components from the model. To recap from the main text, removing adaptive immunity caused infection to become chronic (Figs. Ci-ii); removing innate immunity increased the peak viral load (Figs. Ciii-iv); and removing innate and adaptive immunity increased the peak viral load and delayed resolution of the infection (Figs. Cv-vi). Removing humoral adaptive immunity caused the viral load to rebound instead of continuing to decrease (Figs. Cvii-viii), and removing cellular adaptive immunity delayed resolution of the infection(Figs. Cix-x).

We then compared predictions of the viral load for a single infection by the models fitted to the sequential and single infection datasets. Results were the same as for the main text. Chronic infection in the absence of adaptive immunity was only predicted using sequential infection data (Figs. Ci-ii). Single infection data did not enable consistent prediction of this outcome, as indicated by the broadening prediction interval. However, both datasets enabled recovery of the time at which the viral loads in the presence and absence of adaptive immunity deviated (the vertical line in Figs. Ci–ii). Hence, the timing of adaptive immunity was accurately estimated using either dataset.

Figs. Ciii-iv shows that sequential infection data enabled accurate inference of when the viral loads in the presence and absence of innate immunity deviated, hence recovering the timing of innate immunity. By contrast, the model fitted to single infection data predicted that the viral loads could deviate much earlier. Neither model accurately predicted how the infection resolved in the absence of innate immunity; however, the prediction intervals for the model fitted to sequential infection data were tighter, and the peak viral load was consistently predicted to be higher than for the baseline model. Similarly, when both innate and adaptive immunity were absent, the model fitted to sequential infection data recovered the timing of overall immunity, but could not predict the viral load in the absence of immunity (Figs. Cv-vi).

Without innate immunity, the viral load peaks due to target cell depletion, and without any immune response, the infection resolves due to target cell depletion. The lack of predictive ability indicates that both datasets lack information on how target cells would hypothetically become depleted, and how this depletion would affect the viral load, in the absence of the immune response. One is thus cautioned against using parameter values from a model fitted to data

in immunocompetent hosts to make predictions in situations where target cells may become severely depleted, such as if individuals are immunocompromised.

Figs. Cv–viii show that neither dataset enabled prediction of how the viral load changed when (v-vi) the humoral adaptive immune response or (vii-viii) the cellular adaptive immune response was removed. This implies that sequential infection data (of the type reported in Laurie *et al.* [1]) cannot be used to distinguish the contributions of antibodies and cellular adaptive immunity to resolution of infection.

Cross-protection between strains

Given the above mixed results, we then tested whether sequential infection data accurately captured the timing and extent of cross-protection, by simulating the viral load for inter-exposure intervals other than those where data was provided.

Figure D shows prediction intervals for the challenge viral load for inter-exposure intervals of (i, iii) 2 and (ii, iv) 20 days. Like in the main text, the blue areas, which correspond to the model fitted to sequential infection data, accurately predict the viral load for the challenge strain. By contrast, the green areas, which correspond to the model fitted to single infection data, do not accurately predict the viral load for the challenge strain.

Each immune component's contribution to cross-protection

Having accurately recovered the timing and extent of cross-protection between strains, we then asked whether such cross-protection could be attributed to the 'correct' mechanisms (the same mechanisms as given by the 'true' parameters). These mechanisms are

- target cell depletion due to the infection and subsequent death of cells;
- innate immunity; and
- cellular adaptive immunity.

Before analysing the behaviour of the fitted models, we quantified how each immune component contributed to cross-protection for the 'true' parameters. In Fig. E, for a one-day inter-exposure interval, we plotted in red the challenge viral load for the baseline model (all three of the above immune components could mediate cross-protection). We observed that for a one-day inter-exposure interval, the challenge infection was delayed.

We then modified the baseline model such that only a subset of immune components mediated cross-protection. We used different modified models to predict the viral load (in black), and compared them with the baseline viral load.

In Figs. Ei–ii, we modified the baseline model such that only cellular adaptive immunity, and not target cell depletion or innate immunity, can mediate cross-protection. We denoted this modified model 'model XC'. Unlike the baseline model (red dotted line), the challenge viral load for model XC was not

delayed (black solid line); in fact, it closely resembled that for a single infection. Comparing the two simulations led to the conclusion that cellular adaptive immunity did not play a major part in cross-protection for a one-day inter-exposure interval.

We then modified the baseline model such that both target cell depletion and innate immunity can mediate cross-protection, but cellular adaptive immunity cannot do so. We denoted this model 'model XIT'. The challenge viral loads according to model XIT and the baseline model were similarly delayed (Figs. Eiii-iv). Hence, for the 'true' parameters, cross-protection was mediated by innate immunity and/or target cell depletion.

To distinguish between these two mechanisms, we constructed model XI, where only innate immunity, and not target cell depletion or cellular adaptive immunity, can mediate cross-protection. The challenge viral load for model XI was delayed compared to a primary infection, but less so than for model XIT (Figs. Ev-vi). We also constructed model XT, where only target cell depletion, and not innate immunity or cellular adaptive immunity, can mediate cross-protection. The challenge viral load for model XT was similar to that for a primary infection (Figs. Evii-viii). We concluded that the cross-protection was largely mediated by innate immunity, with some contribution by target cell depletion.

We then sampled parameter sets from the joint posterior distributions obtained by fitting the model in the main text to sequential infection data, and used them as inputs for models XC, XIT, XI and XT respectively, to generate the areas in Fig. E. If the modified models made the same predictions using the fitted parameters and the 'true' parameters, then the fitted model attributed cross-protection to the 'correct' mechanisms.

The results were similar to the main text. Models XC and XIT made the same predictions using the fitted parameters (shaded area) and the 'true' parameters (black line), so sequential infection data enabled us to accurately attribute cross-protection to target cell depletion and/or innate immunity, rather than cellular adaptive immunity (Figs. Ei–iv). On the other hand, the fitted parameters did not consistently predict the challenge outcome for models XI and XT; model predictions were accurate for dataset 2, but not dataset 3 (Figs. Ev–viii). Hence, we could not use sequential infection data to consistently quantify the contributions of target cell depletion and innate immunity to cross-protection.

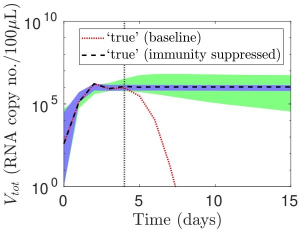

(i) adaptive, dataset 2

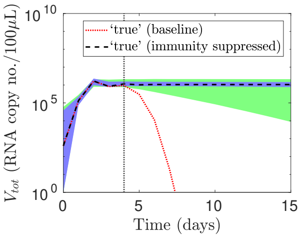

(ii) adaptive, dataset 3

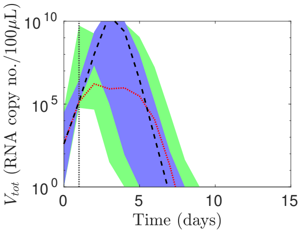

(iii) innate, dataset 2

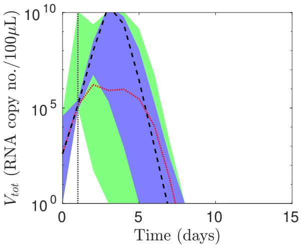

(iv) innate, dataset 3

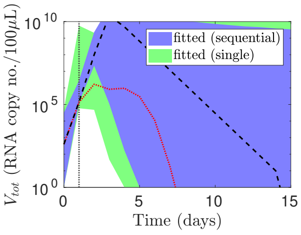

(v) innate and adaptive, dataset 2

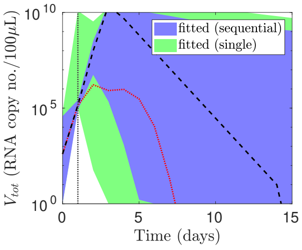

(vi) innate and adaptive, dataset 3

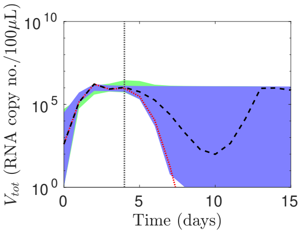

(vii) humoral adaptive, dataset 2

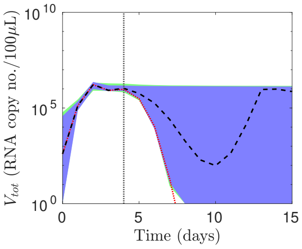

(viii) humoral adaptive, dataset 3

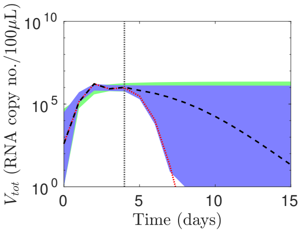

(ix) cellular adaptive, dataset 2

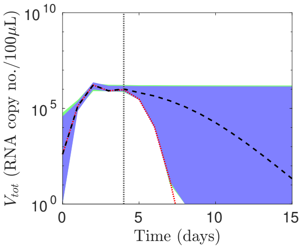

(x) cellular adaptive, dataset 3

Figure C: **Predicting the viral load for a single infection when various immune components were absent.** The vertical lines indicate, for the 'true' parameter values, the times at which the immune components labelled under each panel took effect. These times were determined by when the viral load for the baseline model (red dotted line) deviated from the viral load when the immune components were absent (black dashed line). These times could be recovered using sequential infection data, but single infection data could only recover the timing of innate immunity. Credible intervals for the model fitted to sequential infection data were tighter than for the model fitted to single infection data. Prediction intervals were constructed without measurement noise.

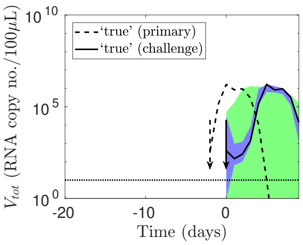

(i) 2-day interval, dataset 2

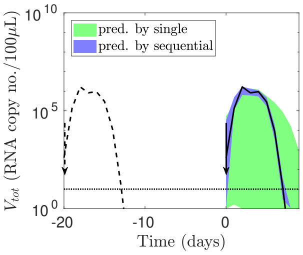

(ii) 20-day interval, dataset 2

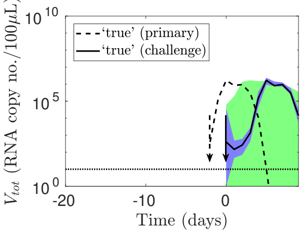

(iii) 2-day interval, dataset 3

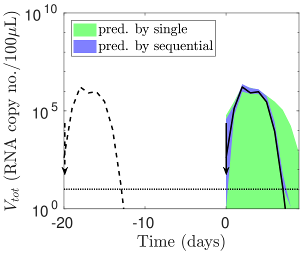

(iv) 20-day interval, dataset 3

Figure D: **Predicting the outcomes of further sequential infection experiments.** Sequential infection data, but not single infection data, enabled prediction of further sequential infection experiment outcomes. The lines show the simulated 'true' viral loads for inter-exposure intervals of (i, iii) 2 and (ii, iv) 20 days. The shaded areas show the 95% prediction intervals for the challenge viral load.

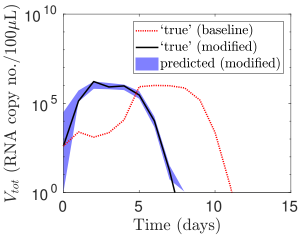

(i) XC, dataset 2

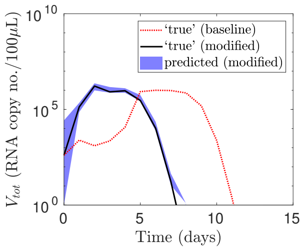

(ii) XC, dataset 3

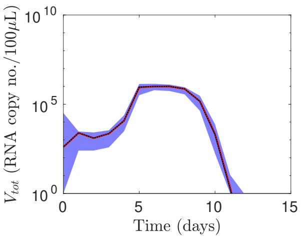

(iii) XIT, dataset 2

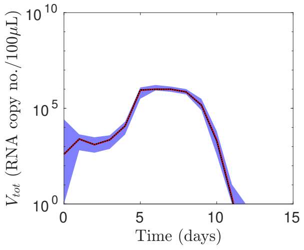

(iv) XIT, dataset 3

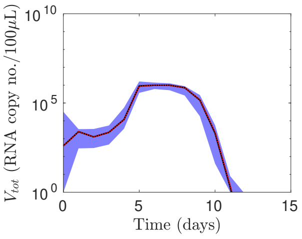

(v) XI, dataset 2

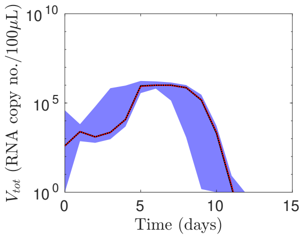

(vi) XI, dataset 3

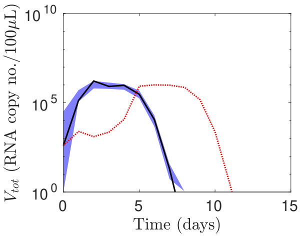

(vii) XT, dataset 2

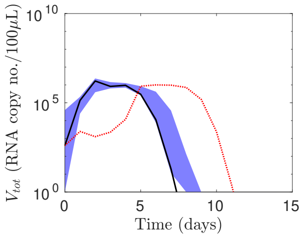

(viii) XT, dataset 3

Figure E: **Predictions of the challenge viral load for a one-day inter-exposure interval when the mechanisms mediating cross-protection were restricted.** The black solid lines show the challenge viral load for the 'true' parameter values when the mechanisms mediating cross-protection were restricted. The red dotted lines show the viral load for the baseline model. Comparing the two sets of lines reveals that target cell depletion and innate immunity mediated cross-protection, whereas cellular adaptive immunity did little to mediate cross-protection. The model fitted to sequential infection data accurately predicted the challenge outcomes for models XC and XIT, but not models XI and XIT (95% prediction intervals shown). It thus correctly attributed cross-protection to target cell depletion and/or innate immunity, but could not definitively distinguish between the two. For clarity, the viral load for the primary infection is not presented in this figure.

References

1. Laurie KL, Guarnaccia TA, Carolan LA, Yan AWC, Aban M, Petrie S, et al. Interval between infections and viral hierarchy are determinants of viral interference following influenza virus infection in a ferret model. J Infect Dis. 2015;212(11):1701–1710. doi:10.1093/infdis/jiv260.
