# Supplementary material for: Sequential infection experiments for quantifying innate and adaptive immunity during influenza infection
Source: PLoS Comput Biol. 2019 Jan 17;15(1):e1006568. doi: 10.1371/journal.pcbi.1006568 (PMC6353225; doi:10.1371/journal.pcbi.1006568)
Supplement: S7 File — (PDF) [file pcbi.1006568.s013.pdf]

Sequential infection experiments for quantifying innate and adaptive immunity during influenza infection

File S7:
Results for a data set generated using a different model

The model

Because the model in the main text does not capture every biological detail of the experimental system, we tested whether our findings were robust to model misspecification. We generated data using a model from a study by Zarnitsyna *et al.* [1], modified to include a variable degree of cross-reactivity between strains. This model is regarded as the 'true' model for this supplementary file. The model equations are given by

$$
\begin{aligned}
\frac{dT}{dt} &= -\beta T \sum_{q=1}^{2} V - k_M M T + g\left(T_0 - T - \sum_{q=1}^{2} I_q\right), \\
\frac{dI_q}{dt} &= \beta T V_q - \sum_{j=1}^{J} k_{Rjq} T_{Rj} I_q - \delta I_q, \qquad q = 1, 2, \\
\frac{dV_q}{dt} &= p I_q - c V_q, \\
\frac{dM}{dt} &= \frac{\sigma_M \sum_{q=1}^{2} I_q}{\phi_M + \sum_{q=1}^{2} I_q}(1 - M) - d_M M, \\
\frac{dA_q}{dt} &= \gamma V_q - d_A A_q, \\
\frac{dT_{Pj}}{dt} &= -\rho T_{Pj} \frac{\sum_{q=1}^{2}(A_q/\phi_{jq})}{1 + \sum_{q=1}^{2}(A_q/\phi_{jq})}, \qquad j = 1, \ldots, J, \\
\frac{dT_{Ej}}{dt} &= \rho\left(T_{Pj} + T_{Ej}\right) \frac{\sum_{q=1}^{2}(A_q/\phi_{jq})}{1 + \sum_{q=1}^{2}(A_q/\phi_{jq})} \\
&\quad - (\alpha + r)\, T_{Ej} \left[1 - \frac{\sum_{q=1}^{2}(A_q/\phi_{jq})}{1 + \sum_{q=1}^{2}(A_q/\phi_{jq})}\right] - \mu T_{Ej} M, \\
\frac{dT_{Mj}}{dt} &= r T_{Ej} \left[1 - \frac{\sum_{q=1}^{2}(A_q/\phi_{jq})}{1 + \sum_{q=1}^{2}(A_q/\phi_{jq})}\right], \\
\frac{dT_{Rj}}{dt} &= \mu T_{Ej} M - d_R T_{Rj}.
\end{aligned}
\tag{1}
$$

Target cells (T) become infected by free virus (V_q, where $q = 1, 2$ denotes the strain number); infected cells (I_q) produce free virus; and infected cells and free virus decay at a constant rate. Target cells also regrow at a rate proportional to the number of dead cells, with a carrying capacity of T_0. The innate immune response is mediated by cytokines (M) which are stimulated by infected cells. Cytokines render target cells temporarily refractory (this compartment is not explicitly tracked). Refractory cells revert to target cells at the same rate as target cells regrow. The cellular adaptive immune response is mediated by T cells. Free virus presents antigen (A), which stimulate the conversion of precursor T cells (T_P) into proliferating T cells (T_E). The T cell pool number is labelled j; for the baseline model, the number of T cell pools is $J = 3$. Proliferating T cells reproduce through stimulation by antigen. Cytokines trigger the migration of proliferating T cells to the site of infection, where they become resident T cells (T_R). These resident T cells (T_R) increase the death rate of infected cells. In the absence of antigen, proliferating T cells convert into memory T cells (T_M). The humoral immune response is absent from this model.

The adaptations from the study by Zarnitsyna *et al.* [1] were:

- The model was extended to two strains, sharing a target cell pool and innate immunity;
- The model was extended to multiple pools of T cells, which have different degrees of cross-reactivity with each strain;
- Target cells regrow;
- Refractory cells revert to target cells.

The main differences between the model in the main text of our study and the model in Eq. 1 are:

- Our model has two additional innate immune mechanisms;
- In our model, innate and adaptive immunity function independently, while in the model in Eq. 1, cytokines stimulate migration of proliferating T cells to the site of infection;
- In our model, memory T cells can be reactivated on the timescale of interest (1-14 days), while in the model in Eq. 1, memory T cells are not reactivated during this time;
- Our model includes humoral adaptive immunity;
- Our model has separate equations for infectious and total virus, while the model in Eq. 1 assumes that the amount of infectious and total virus is the same;
- Our model does not distinguish between proliferating and resident T cells;
- Our model assumes that antigen is proportional to viral load (in the case of B cell stimulation) or infected cell count (in the case of T cell stimulation), while in the model in Eq. 1, free virus converts to antigen.

The observation model is the same as for the model in the main text.

Table A shows the parameters and initial values used the generate the data. All compartments without specified initial values are set to zero. They were modified from the parameters in the study by Zarnitsyna *et al.* [1], to generate the qualitative behaviour observed in the study by Laurie *et al.* [2] for infection with influenza A followed by influenza B, or vice versa.

Figure A shows a subset of the synthetic data.

Parameter	Description	Value	Units
β	Virus infectivity	3×10^{-5}	(RNA copies/100μL)$^{-1}$ day^{-1}
k_M	Rate at which target cells become refractory	8	cell day^{-1}
p	Virus production per cell	0.04	RNA copies/100μL day^{-1}
c	Rate of virus clearance	3	day^{-1}
δ	Rate of infected cell clearance	1	day^{-1}
σ_M	Maximum activation rate for innate immunity	2	day^{-1}
ϕ_M	Number of infected cells for half-maximum activation of M	2	cells
d_M	Decay rate for innate immunity	0.4	day^{-1}
γ	Rate of virus conversion to antigen	0.6	day^{-1}
d_A	Decay rate of antigen	3.4	day^{-1}
ρ	T cell proliferation rate	4.3	day^{-1}
μ	Rate of migration to site of infection	2.4	cell^{-1} day^{-1}
r	Rate of conversion of T_E to T_M	0.14	day^{-1}
α	Rate of apoptosis for T_E	0.8	day^{-1}
d_R	Death rate of T_R	0.2	day^{-1}
g	Target cell regrowth rate and rate of reversion from refractory to target cell state	0.5	day^{-1}
T_0	Initial number of target cells	4×10^8	cells
V_0	Initial viral load	10	RNA copies/100μL
M_0	Initial cytokine level	10^{-6}	normalised
$T_{Pj,0}$	Initial precursor T cell level	1	cell
ϕ_{jq}	Antigen for half-maximum T cell proliferation	$\phi_{11} = \phi_{12} = 50.51$<br>$\phi_{21} = \phi_{12} = \infty$<br>$\phi_{31} = \phi_{32} = 5000$	RNA copies/100μL
k_{Rjq}	Rate of killing of infected cells by T_R	$k_{R11} = k_{R22} = 0.01386$<br>$k_{R12} = k_{R21} = 0$<br>$k_{R31} = k_{R32} = 0.00014$	cell^{-1} day^{-1}

Table A: Parameters used to generate the data.

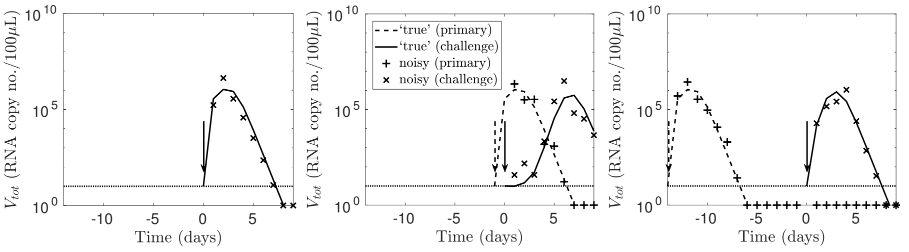

(i) single infection (ii) 1-day interval (iii) 14-day interval

Figure A: **A subset of the synthetic data.** (i) The line shows the simulated 'true' viral load for a single infection, with the arrow showing the time of exposure. The simulated viral load with noise is shown as crosses. The horizontal line indicates the observation threshold (10 RNA copy no./100μL); observations below this threshold are plotted below this line. Values below the observation threshold were treated as censored. (ii–iii) For sequential infections with the labelled inter-exposure interval, the dashed and dotted lines show the simulated 'true' viral load for a primary and challenge infection respectively; the arrows show the times of the primary and challenge exposures. The simulated viral load with noise is shown as crosses.

Results

Verification of the fitting procedure

We fitted the model in the main text to the data in Fig. A, to test whether the simulated 'true' viral load can be recovered despite model misspecification. Figure B presents 95% credible intervals for the viral load. The credible intervals included the 'true' viral load, confirming accurate recovery.

Comparing the immunological information in each dataset

Next, we compared the behaviour of the fitted models to the behaviour of the model in Eq. 1, to determine the information in each dataset on

- the effect of each immune component in controlling a single infection;
- cross-protection between strains; and
- each immune component's contribution to cross-protection.

The effect of each immune component in controlling a single infection

In Fig. C, we removed various immune components from the model in Eq. 1. Adaptive immunity was removed by setting k_R, the rate of killing of infected cells by resident T cells, to zero. For the model in Eq. 1, this caused the infection to become chronic (Fig. Ci). Since the viral load without adaptive immunity

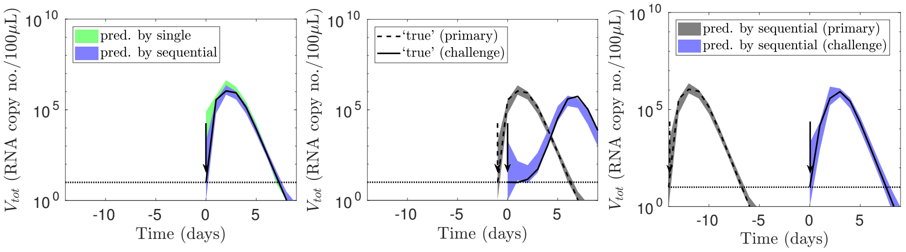

(i) single infection (ii) 1-day interval (iii) 14-day interval

Figure B: **Verification that the fitting procedure recovered the viral load.** (i) For a single infection, the blue and green areas are the 95% credible intervals for the viral load (in the absence of noise), as predicted by the models fitted to the sequential infection and single infection data respectively. (ii–iii) For sequential infections with the labelled inter-exposure interval, the grey and blue areas show the 95% credible intervals for the primary and challenge viral load respectively, predicted by the model fitted to sequential infection data. The other elements of the figure are identical to Fig 1 in the main text: the dashed and dotted lines show the simulated 'true' viral load for a primary and challenge infection respectively; the arrows show the times of the primary and challenge exposures; and the horizontal line indicates the observation threshold.

deviated from that with adaptive immunity at two to three days post-infection, we inferred that adaptive immunity was activated at two to three days post-infection in the baseline model. (The deviation between two and three days post-infection is small, making it difficult to precisely define the timing of adaptive immunity for these parameters.) Innate immunity was removed by setting k_M, the rate at which cytokines convert target cells to the refractory state, to 0. We allowed the stimulation of adaptive immunity by innate immunity to remain. (If we do not allow the stimulation of adaptive immunity by innate immunity to remain, removing innate immunity has the same effect as removing both innate and adaptive immunity.) Removing innate immunity increased the peak viral load without delaying resolution of the infection (Fig. Cii). Removing both innate and adaptive immunity increased the peak viral load as well as causing the infection to become chronic (Fig. Ciii). As humoral adaptive immunity is absent from the model, removing humoral adaptive immunity had no effect (Fig. Civ), and removing cellular adaptive immunity was the same as removing all adaptive immunity (Fig. Cv).

The model fitted to sequential infection data was able to estimate the timing of immune components. For the fitted model, the viral load without adaptive immunity deviated from the baseline around three days post-infection, which is similar to the two to three days observed for the 'true' model and parameters (Fig. Ci). The viral load without innate immunity deviated from the baseline one day post-infection, which is the same as for the 'true' model and parameters (Fig. Cii). However, unlike in the main text, the fitted model was unable to

predict the viral load in the absence of adaptive immunity, as shown by the wide credible interval in Fig. Ci. A possible explanation for the different result is that for the parameters in the main text, the viral load showed a clear plateau while innate but not adaptive immunity was active, enabling the fitted model to predict that the viral load would stay at that plateau in the absence of adaptive immunity. By contrast, the viral load in Fig. A in this text did not show a clear plateau during the innate immunity phase, possibly reducing the fitted model's ability to infer the viral load in the absence of adaptive immunity. The fitted model was also unable to predict the viral load in the absence of innate immunity, or distinguish between humoral and cellular adaptive immunity (Figs. Cii–v). These results are consistent with the main text. The model fitted to single infection data was only able to infer the timing of adaptive immunity and not innate immunity. Also, the credible intervals for the viral load predicted by the model fitted to single infection data were wider. These results are consistent with the main text.

Cross-protection between strains

Given the above mixed results, we then tested whether sequential infection data accurately captured the timing and extent of cross-protection, by simulating the viral load for inter-exposure intervals other than those where data was provided.

Figure D shows prediction intervals for the challenge viral load for inter-exposure intervals of (i) 2 and (ii) 20 days. Like in the main text, the blue areas, which correspond to the model fitted to sequential infection data, accurately predict the viral load for the challenge strain. By contrast, the green areas, which correspond to the model fitted to single infection data, do not accurately predict the viral load for the challenge strain.

Each immune component's contribution to cross-protection

Having accurately recovered the timing and extent of cross-protection between strains, we then asked whether such cross-protection could be attributed to the 'correct' mechanisms (the same mechanisms as given by the 'true' model and parameters). These mechanisms are

- target cell depletion due to the infection and subsequent death of cells;
- innate immunity; and
- cellular adaptive immunity.

Before analysing the behaviour of the fitted models, we quantified how each immune component contributed to cross-protection for the 'true' model parameters. In Fig. E, for a one-day inter-exposure interval, we plotted in red the challenge viral load for the baseline model in Eq. 1, where all three of the above immune components could mediate cross-protection. We observed that for a one-day inter-exposure interval, the challenge infection was delayed.

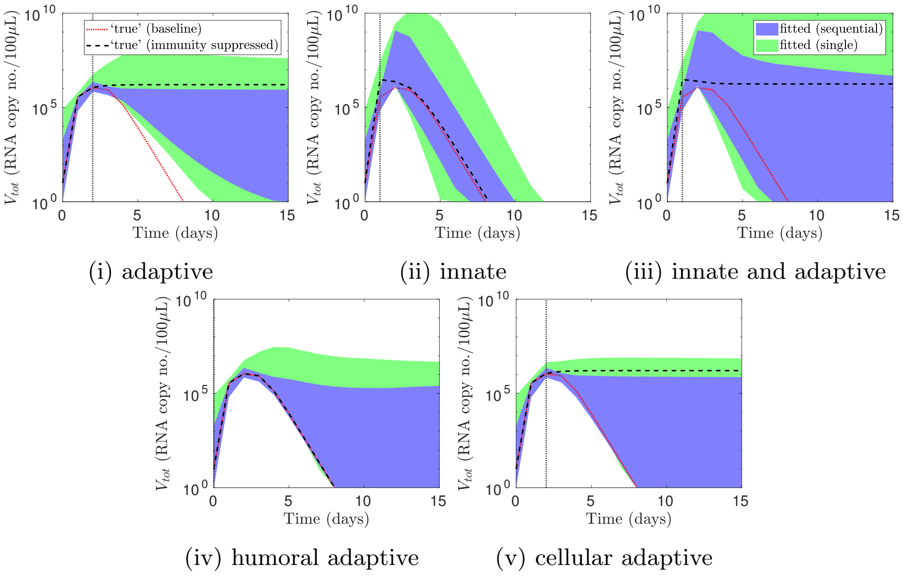

Figure C: **Predicting the viral load for a single infection when various immune components were absent.** The vertical lines indicate, for the ‘true’ model and parameter values, the times at which the immune components labelled under each panel took effect. (This line is not plotted for humoral adaptive immunity as removing it had no effect.) These times were determined by when the viral load for the baseline model (red dotted line) deviated from the viral load when the immune components were absent (black dashed line). These times could be recovered using sequential infection data, but single infection data could only recover the timing of adaptive immunity. Credible intervals for the model fitted to sequential infection data were tighter than for the model fitted to single infection data. Prediction intervals were constructed without measurement noise.

We then modified the baseline model such that only a subset of immune components mediate cross-protection, as detailed in the following section. We used the modified model to predict the viral load (in black), and compared it with the baseline viral load.

In Fig. Ei, we modified the baseline model such that only cellular adaptive immunity, and not target cell depletion or innate immunity, can mediate cross-protection. We denoted this modified model ‘model XC’. Unlike the baseline model (red dotted line), the challenge viral load for model XC was not delayed (black solid line); in fact, it closely resembled that for a single infection. Comparing the two simulations led to the conclusion that cellular adaptive immunity did not play a major part in cross-protection for a one-day inter-exposure interval.

We then modified the baseline model such that both target cell depletion

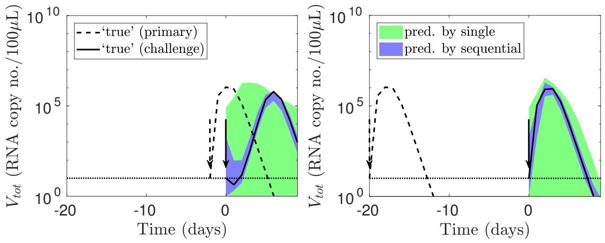

(i) 2-day interval (ii) 20-day interval

Figure D: **Predicting the outcomes of further sequential infection experiments.** Sequential infection data, but not single infection data, enabled prediction of further sequential infection experiment outcomes. The lines show the simulated 'true' viral loads for inter-exposure intervals of (i) 2 and (ii) 20 days. The shaded areas show the 95% prediction intervals for the challenge viral load.

and innate immunity can mediate cross-protection, but cellular adaptive immunity cannot do so. We denoted this model 'model XIT'. The challenge viral loads according to model XIT and the baseline model were similarly delayed (Fig. Eii). Hence, for the 'true' parameters, cross-protection was mediated by innate immunity and/or target cell depletion.

To distinguish between these two mechanisms, we constructed model XI, where only innate immunity, and not target cell depletion or cellular adaptive immunity, can mediate cross-protection. The challenge viral load for model XI was delayed compared to a primary infection, but less so than for model XIT (Fig. Eiii). We also constructed model XT, where only target cell depletion, and not innate immunity or cellular adaptive immunity, can mediate cross-protection. The challenge viral load for model XT was similar to that for a primary infection (Fig. Eiv). We concluded that the cross-protection was largely mediated by innate immunity, with some contribution by target cell depletion.

We then sampled parameter sets from the joint posterior distributions obtained by fitting the model in the main text to sequential infection data, and used them as inputs for models XC, XIT, XI and XT respectively, to generate the areas in Fig. E. If the modified models made the same predictions using the fitted parameters and the 'true' parameters, then the fitted model attributed cross-protection to the 'correct' mechanisms.

The results were similar to the main text. Models XC and XIT made the same predictions using the fitted parameters (shaded area) and the 'true' parameters (black line), so sequential infection data enabled us to accurately attribute cross-protection to target cell depletion and/or innate immunity, rather than cellular adaptive immunity (Figs. Ei–ii). On the other hand, the fitted parameters did not consistently predict the challenge outcome for model XI, although predictive performance was better for model XT (Figs. Eiii–iv). Hence, we could not use sequential infection data to consistently quantify the contributions of

target cell depletion and innate immunity to cross-protection.

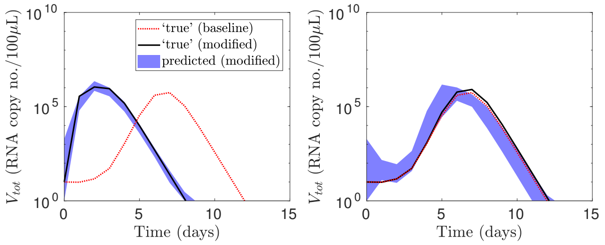

(i) XC　　(ii) XIT

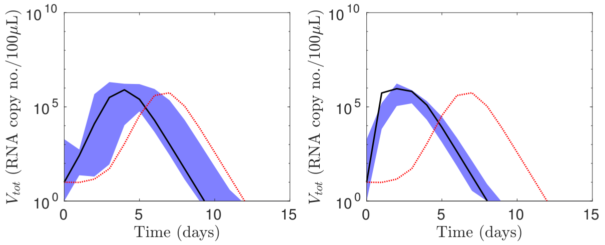

(iii) XI　　(iv) XT

Figure E: **Predictions of the challenge viral load for a one-day inter-exposure interval when the mechanisms mediating cross-protection were restricted.** The black solid lines show the challenge viral load for the 'true' parameter values when the mechanisms mediating cross-protection were restricted. The red dotted lines show the viral load for the baseline model. Comparing the two sets of lines reveals that target cell depletion and innate immunity mediated cross-protection, whereas cellular adaptive immunity did little to mediate cross-protection. The model fitted to sequential infection data accurately predicted the challenge outcomes for models XC, XIT and XT, but not model XI (95% prediction intervals shown). It thus correctly attributed cross-protection to target cell depletion and/or innate immunity, but could not definitively distinguish between the two. The viral load for the primary infection is not shown, to improve clarity of the figure.

Equations for models XC, XI, XT and XIT

In model XC, cross-protection is mediated by cellular adaptive immunity only, and not target cell depletion or innate immunity. Unlike the baseline model, cytokines M_q are strain-specific. Cells infected with strain q induce the production of cytokines specific to that strain. These cytokines M_q only render target cells temporarily resistant to infection with strain q. However, stimulation of T cell migration by cytokines remains non-strain-specific. This stimulation is now proportional to the mean cytokine level across strains, introducing a factor of 2 in the last line of Eq. 2. In addition, each strain now targets a separate pool of

uninfected cells; the size of each target cell pool is T_0 (identical for both strains). This alternative model is not meant to reflect a biologically realistic situation; however, it enables *in silico* thought experiments to determine, for a given set of parameters, the contribution of each immune component to cross-protection.

Explicitly, the changed equations (relative to Eq. 1) for model XC are given by

$$\begin{aligned}
\frac{dT_q}{dt} &= -\beta T_q V_q - k_M M_q T_q + g(T_0 - T_q - I_q), \qquad q = 1, 2, \\
\frac{dI_q}{dt} &= \beta T_q V_q - \sum_{j=1}^{J} k_{Rjq} T_{Rj} I_q - \delta I_q, \\
\frac{dM_q}{dt} &= \frac{\sigma_M I_q}{\phi_M + I_q}(1 - M_q) - d_M M_q, \\
\frac{dT_{Rj}}{dt} &= \mu T_{Ej} \sum_{q=1}^{2} M_q/2 - d_R T_{Rj}, \qquad j = 1, \dots, J.
\end{aligned} \tag{2}$$

The other equations in Eq. 1 remain unchanged.

In model XI, cross-protection is mediated by innate immunity, but not target cell depletion or cellular adaptive immunity. The model is altered from the baseline model such that the number of T cell pools is $J = 4$. T cell pools 3 and 4 have the same parameters as pool 3 in the baseline model, but T cell pool 3 is stimulated by antigen from strain 1 only, and targets cells infected with strain 1 only, while T cell pool 4 is stimulated by antigen from strain 2 only, and targets cells infected with strain 2 only. (Explicitly, the cross-reactivity parameters for T cell pools 3 and 4 are $\phi_{R31} = \phi_{R42}$ equal to $\phi_{R31} = \phi_{R32}$ in the baseline model; $\phi_{R32} = \phi_{R41} = \infty$; $k_{R31} = k_{R42}$ equal to $k_{R31} = k_{R32}$ in the baseline model; and $k_{R32} = k_{R42} = 0$.) The cross-reactivity parameters for pools 1 and 2 remain the same. In addition, each strain now has its own target cell pool.

The equations for model XI which are altered relative to Eq. 1 are

$$\begin{aligned}
\frac{dT_q}{dt} &= -\beta T_q V_q - k_M M T_q + g(T_0 - T_q - I_q), \qquad q = 1, 2, \\
\frac{dI_q}{dt} &= \beta T_q V_q - \sum_{j=1}^{J} k_{Rjq} T_{Rj} I_q - \delta I_q, \qquad q = 1, 2.
\end{aligned} \tag{3}$$

In the model where cross-protection is mediated by target cell depletion and/or innate immunity, but not cellular adaptive immunity (model XIT), the cellular adaptive immune response is no longer cross-reactive. Like for model XI, the model is altered from the baseline model such that the number of T cell pools is $J = 4$. However, like the baseline model, target cells are shared between the two strains. Hence, the alterations in Eq. 3 are not made for model XIT.

In the model where cross-protection is mediated by target cell depletion only, the model is altered from the baseline model such that the number of T cell pools is $J = 4$. In addition, cytokines M_q are strain-specific. Cells infected

with strain q induce the production of cytokines specific to that strain. These cytokines M_q only render target cells temporarily resistant to infection with strain q. However, the virus strains still share a target cell pool, so to implement target cells becoming temporarily resistant to infection with strain q, we now explicitly track refractory cells resistant to each strain, R_q, and refractory cells resistant to both strains, R_{12}.

The changed equations (relative to Eq. 1) for model XT are given by

$$
\begin{aligned}
\frac{dT}{dt} &= -\beta T \sum_{q=1}^{2} V_q - k_M \sum_{q=1}^{2} M_q T + g\left(T_0 - T - \sum_{q=1}^{2} I_q - R_{12}\right), \\
\frac{dR_q}{dt} &= k_M M_q T - \beta R_q \sum_{q' \neq q} V_{q'} - k_M \sum_{q' \neq q} M_{q'} R_q + g R_{12}, \qquad q = 1, 2, \\
\frac{dR_{12}}{dt} &= k_M \sum_{q=1}^{2} \left(\sum_{q' \neq q} M_{q'} R_q \right) - 2 g R_{12}, \\
\frac{dI_q}{dt} &= \beta \left(T + \sum_{q' \neq q} R_{q'} \right) V_q - \sum_{j=1}^{J} k_{Rjq} T_{Rj} I_q - \delta I_q, \\
\frac{dM_q}{dt} &= \frac{\sigma_M I_q}{\phi_M + I_q}(1 - M_q) - d_M M_q, \\
\frac{dT_{Rj}}{dt} &= \mu T_{Ej} \sum_{q=1}^{2} M_q/2 - d_R T_{Rj}, \qquad j = 1, \ldots, J.
\end{aligned}
\tag{4}
$$

References

1. Zarnitsyna VI, Handel A, McMaster SR, Hayward SL, Kohlmeier JE, Antia R. Mathematical model reveals the role of memory CD8 T cell populations in recall responses to influenza. Front Immunol. 2016;7(May):165. doi:10.3389/fimmu.2016.00165.

2. Laurie KL, Guarnaccia TA, Carolan LA, Yan AWC, Aban M, Petrie S, et al. Interval between infections and viral hierarchy are determinants of viral interference following influenza virus infection in a ferret model. J Infect Dis. 2015;212(11):1701–1710. doi:10.1093/infdis/jiv260.
