# Supplementary material for: Sequential infection experiments for quantifying innate and adaptive immunity during influenza infection
Source: PLoS Comput Biol. 2019 Jan 17;15(1):e1006568. doi: 10.1371/journal.pcbi.1006568 (PMC6353225; doi:10.1371/journal.pcbi.1006568)
Supplement: S1 Table — Note that the values and prior bounds are given in logarithmic space. For example, the value of log10 R0 was log104.9 and the prior bounds were [0, 3]. Hence, the value of R0 was 4.9 and the prior bounds of R0 were [1, 1000]. β and pVinf were not directly fitted, but their values as recovered from Eqs 7 and 9 could not exceed the bounds given. Because total virions include infectious virions, the total virion decay rate should be slower than the infectious virion decay rate. Hence, the difference between the infectious and total virion decay rates δVinf − δVtot, rather than the infectious virion decay rate δVinf, was fitted to ensure that the former quantity was positive. Notes on biologically plausible ranges for the parameters ptot, α and γ are given in S2 Text. (PDF) [file pcbi.1006568.s014.pdf]

Table S1

Parameter	Description	Value	Prior bounds	Units
$\log_{10} R_0$	basic reproduction number	$\log_{10} 4.9$	$[0, 3]$ [1, 2, 3, 4, 5, 6]	
$\log_{10} r$	initial viral growth rate	$\log_{10} 6.56$	$[0, 2]$ [1, 2, 3, 4, 5, 6]	day^{-1}
$\log_{10} \delta_I$	infected cell decay rate	$\log_{10} 2$	$[-1, 2]$ [7]	day^{-1}
$\log_{10}(\delta_{Vinf} - \delta_{Vtot})$	difference between infectious and total virion decay rates	$\log_{10} 2$	$[-2, 2]$ [8]	day^{-1}
$\log_{10} \delta_{Vtot}$	total virion decay rate	$\log_{10} 8$	$[0, 2]$ [7]	day^{-1}
$\log_{10} T_0$	initial number of target cells	$\log_{10}(7 \times 10^7)$	$[7, 8]$ [8]	target cell
$\log_{10} g$	target cell regrowth rate	$\log_{10} 0.8$	$[-10, 0.5]$ [9, 10, 11, 12, 13]	day^{-1}
$\log_{10} p_{Vratio}$	ratio of production of total to infectious virions	$\log_{10}(4 \times 10^4)$	$[0, 6]$	
$\log_{10} \alpha$	the number of RNA copies/100μL of nasal wash corresponding to one virion	$\log_{10} 0.01$	$[-3, -1]$	RNA copies/100μL virion^{-1}
$\log_{10} \gamma$	the initial ratio of total to infectious virions	$\log_{10}(4 \times 10^4)$	$[0, 5]$	
$\log_{10} V_{inf0}$	the number of infectious virions upon exposure	$\log_{10} 1$	$[0, 3]$	virion
$\log_{10} \beta$	infectivity parameter	$\log_{10}(5 \times 10^{-7})$	$[-12, -4]$ [7]	virion^{-1} day^{-1}
$\log_{10} p_{Vinf}$	production rate of infectious virions by infected cells	$\log_{10} 12.6$	$[-6, 6]$ [7]	virion infected cell^{-1} day^{-1}

References

[1] Handel A, Longini Jr IM, Antia R. Neuraminidase inhibitor resistance in influenza: assessing the danger of its generation and spread. PLoS Comput Biol. 2007;3(12):e240. doi:10.1371/journal.pcbi.0030240.

[2] Miao H, Hollenbaugh JA, Zand MS, Holden-Wiltse J, Mosmann TR, Perelson AS, et al. Quantifying the early immune response and adaptive immune response kinetics in mice infected with influenza A virus. J Virol. 2010;84(13):6687–6698. doi:10.1128/JVI.00266-10.

[3] Pawelek KA, Huynh GT, Quinlivan M, Cullinane A, Rong L, Perelson AS. Modeling within-host dynamics of influenza virus infection including immune responses. PLoS Comput Biol. 2012;8(6):e1002588. doi:10.1371/journal.pcbi.1002588.

[4] Saenz RA, Quinlivan M, Elton D, MacRae S, Blunden AS, Mumford JA, et al. Dynamics of influenza virus infection and pathology. J Virol. 2010;84(8):3974–3983. doi:10.1128/JVI.02078-09.

[5] Smith AM, Perelson AS. Influenza A virus infection kinetics: quantitative data and models. Wiley Interdiscip Rev Syst Biol Med. 2011;3(4):429–445. doi:10.1002/wsbm.129.

[6] Baccam P, Beauchemin C, Macken CA, Hayden FG, Perelson AS. Kinetics of influenza A virus infection in humans. J Virol. 2006;80(15):7590–7599. doi:10.1128/JVI.01623-05.

[7] Petrie SM, Butler J, Barr IG, McVernon J, Hurt AC, McCaw JM. Quantifying relative within-host replication fitness in influenza virus competition experiments. J Theor Biol. 2015;382:259–271. doi:10.1016/j.jtbi.2015.07.003.

[8] Petrie SM, Guarnaccia T, Laurie KL, Hurt AC, McVernon J, McCaw JM. Reducing uncertainty in within-host parameter estimates of influenza infection by measuring both infectious and total viral load. PLoS ONE. 2013;8(5):e64098. doi:10.1371/journal.pone.0064098.

[9] Möhler L, Flockerzi D, Sann H, Reichl U. Mathematical model of influenza A virus production in large-scale microcarrier culture. Biotechnol Bioeng. 2005;90(1):46–58. doi:10.1002/bit.20363.

[10] Handel A, Longini Jr IM, Antia R. Towards a quantitative understanding of the within-host dynamics of influenza A infections. J R Soc Interface. 2010;7(42):35–47. doi:10.1098/rsif.2009.0067.

[11] Lee HY, Topham DJ, Park SY, Hollenbaugh J, Treanor J, Mosmann TR, et al. Simulation and prediction of the adaptive immune response to influenza A virus infection. J Virol. 2009;83(14):7151–7165. doi:10.1128/JVI.00098-09.

[12] Hancioglu B, Swigon D, Clermont G. A dynamical model of human immune response to influenza A virus infection. J Theor Biol. 2007;246(1):70–86. doi:10.1016/j.jtbi.2006.12.015.

[13] Bocharov GA, Romanyukha AA. Mathematical model of antiviral immune response III. Influenza A virus infection. J Theor Biol. 1994;167(4):323–360.
