# Supplementary material for: Sequential infection experiments for quantifying innate and adaptive immunity during influenza infection
Source: PLoS Comput Biol. 2019 Jan 17;15(1):e1006568. doi: 10.1371/journal.pcbi.1006568 (PMC6353225; doi:10.1371/journal.pcbi.1006568)
Supplement: S2 Table — (PDF) [file pcbi.1006568.s015.pdf]

Table S2

Parameter	Description	Value	Prior bounds	Units
$\log_{10} \delta_F$	interferon decay rate	$\log_{10} 2$	$[-1, 2]$ [1, 2]	day^{-1}
$\log_{10} \phi$	rate at which target cells become resistant to infection	$\log_{10}(3.3 \times 10^{-5})$	$[-10, 0]$ [3, 1]	day^{-1}
$\log_{10} \rho$	rate at which resistant cells become susceptible to infection	$\log_{10} 2$	$[0, 2]$ [2, 4, 5, 6, 1]	day^{-1}
$\log_{10} s$	factor by which the production rate of virions is decreased by type I interferon	5×10^{-4}	$[-10, 0]$	day^{-1}
$\log_{10} \kappa_F$	clearance rate of infected cells by natural killer cells	$\log_{10}(2.5 \times 10^{-3})$	$[-10, 0]$ [1]	day^{-1}

References

[1] Pawelek KA, Huynh GT, Quinlivan M, Cullinane A, Rong L, Perelson AS. Modeling within-host dynamics of influenza virus infection including immune responses. PLoS Comput Biol. 2012;8(6):e1002588. doi:10.1371/journal.pcbi.1002588.

[2] Bocharov GA, Romanyukha AA. Mathematical model of antiviral immune response III. Influenza A virus infection. J Theor Biol. 1994;167(4):323–360.

[3] Saenz RA, Quinlivan M, Elton D, MacRae S, Blunden AS, Mumford JA, et al. Dynamics of influenza virus infection and pathology. J Virol. 2010;84(8):3974–3983. doi:10.1128/JVI.02078-09.

[4] Hancioglu B, Swigon D, Clermont G. A dynamical model of human immune response to influenza A virus infection. J Theor Biol. 2007;246(1):70–86. doi:10.1016/j.jtbi.2006.12.015.

[5] Heffernan JM, Keeling MJ. An in-host model of acute infection: measles as a case study. Theor Popul Biol. 2008;73(1):134–147. doi:10.1016/j.tpb.2007.10.003.

[6] Chang DB, Young CS. Simple scaling laws for influenza A rise time, duration, and severity. J Theor Biol. 2007;246(4):621–635. doi:10.1016/j.jtbi.2007.02.004.
