# Supplementary material for: Sequential infection experiments for quantifying innate and adaptive immunity during influenza infection
Source: PLoS Comput Biol. 2019 Jan 17;15(1):e1006568. doi: 10.1371/journal.pcbi.1006568 (PMC6353225; doi:10.1371/journal.pcbi.1006568)
Supplement: S3 Table — The number of infected cells for half-maximal stimulation of naive/memory CD8+ T cells kCjq and the clearance rate of infected cells by effector CD8+ T cells κE11. (PDF) [file pcbi.1006568.s016.pdf]

Table S3

Experiment	Parameter	Value	Prior bounds	units
Single infection	$\log_{10} k_{C11}$	5.05	$[-1, 10]$	infected cell
	$\log_{10} k_{C21}$	6	$[-1, 10]$	infected cell
	$\log_{10} \kappa_{E11}$	$\log_{10} 0.0081$	$[-10, 3]$ [1]	day^{-1}
Sequential infection	$\log_{10} k_{C11} = \log_{10} k_{C22}$	5.05	$[-1, 10]$	infected cell
	$k_{C12} = k_{C21}$	∞	fixed	infected cell
	$\log_{10} k_{C31} = \log_{10} k_{C32}$	6	$[-1, 10]$	infected cell
	$\log_{10} \kappa_{E11}$	$\log_{10} 0.0081$	$[-10, 3]$ [1]	day^{-1}

References

[1] Barchet W, Oehen S, Klenerman P, Wodarz D, Bocharov G, Lloyd AL, et al. Direct quantitation of rapid elimination of viral antigen-positive lymphocytes by antiviral CD8(+) T cells in vivo. Eur J Immunol. 2000;30(5):1356–63. doi:10.1002/(SICI)1521-4141(200005)30:5¡1356::AID-IMMU1356¿3.0.CO;2-K.
