# Supplementary material for: Sequential infection experiments for quantifying innate and adaptive immunity during influenza infection
Source: PLoS Comput Biol. 2019 Jan 17;15(1):e1006568. doi: 10.1371/journal.pcbi.1006568 (PMC6353225; doi:10.1371/journal.pcbi.1006568)
Supplement: S4 Table — (PDF) [file pcbi.1006568.s017.pdf]

Table S4

Parameter	Description	Value	Prior bounds	Units
$\log_{10} k_B$	concentration of viral RNA for half-maximal stimulation of naive B cells	$\log_{10}(2 \times 10^8)$	$[1, 10]$	RNA copy no./100μL
$\log_{10} \beta_B$	maximal stimulation rate of naive B cells	$\log_{10} 1$	$[-5, 5]$	day^{-1}
$\log_{10} \tau_B$	total proliferation time of plasmablasts	$\log_{10} 4$	$[0, 1]$ [1, 2]	day
$\log_{10} \kappa_A$	neutralisation rate of virions by antibodies	$\log_{10} 360$	$[-10, 10]$ [3]	day^{-1}
$\log_{10} \delta_A$	antibody decay rate	$\log_{10} 0.04$	$[-2, -1]$ [4, 5]	day^{-1}
$\log_{10} \delta_B$	plasmablast and plasma cell decay rate	$\log_{10} 0.11$	$[-1, 0]$ [6, 7]	day^{-1}
$\log_{10} \beta_C$	maximal stimulation rate of naive/memory CD8$^+$ T cells	$\log_{10} 1$	$[-5, 5]$	day^{-1}
$\log_{10} \tau_E$	total proliferation time of effector CD8$^+$ T cells	$\log_{10} 5$	$[0.5, 1]$ [8]	day
$\log_{10} \delta_E$	decay rate of effector CD8$^+$ T cells	$\log_{10} 0.6$	$[-1, 0]$ [9]	day^{-1}
$\log_{10} \epsilon$	proportion of effector CD8$^+$ T cells which become memory CD8$^+$ T cells	$\log_{10} 0.02$	$[-3, -1]$ [10, 11, 12]	
$\log_{10} \tau_M$	mean refractory time for memory CD8$^+$ T cells	$\log_{10} 14$	$[1, 1.5]$ [13]	day
σ	measurement error	0.5	$[0, 2]$	

References

[1] Marchuk GI, Petrov RV, Romanyukha AA, Bocharov GA. Mathematical model of antiviral immune response. I. Data analysis, generalized picture construction and parameters evaluation for hepatitis B. J Theor Biol. 1991;151(1):1–40. doi:10.1016/S0022-5193(05)80142-0.

[2] Sze DMY, Toellner KM, García de Vinuesa C, Taylor DR, MacLennan ICM. Intrinsic constraint on plasmablast growth and extrinsic limits of plasma cell survival. J Exp Med. 2000;192(6):813–821. doi:10.1084/jem.192.6.813.

[3] Miao H, Hollenbaugh JA, Zand MS, Holden-Wiltse J, Mosmann TR, Perelson AS, et al. Quantifying the early immune response and adaptive immune response kinetics in mice infected with influenza A virus. J Virol. 2010;84(13):6687–6698. doi:10.1128/JVI.00266-10.

[4] Bocharov GA, Romanyukha AA. Mathematical model of antiviral immune response III. Influenza A virus infection. J Theor Biol. 1994;167(4):323–360.

[5] Lee HY, Topham DJ, Park SY, Hollenbaugh J, Treanor J, Mosmann TR, et al. Simulation and prediction of the adaptive immune response to influenza A virus infection. J Virol. 2009;83(14):7151–7165. doi:10.1128/JVI.00098-09.

[6] Bortnick A, Allman D. What is and what should always have been: long-lived plasma cells induced by T cell-independent antigens. J Immunol. 2013;190(12):5913–5918. doi:10.4049/jimmunol.1300161.

[7] Minges Wols HA. Plasma Cells. In: eLS. 2006. Chichester, UK: John Wiley & Sons, Ltd; 2015. p. 1–11.

[8] Lehmann-Grube F, Assmann U, Löliger C, Moskophidis D, Löhler J. Mechanism of recovery from acute virus infection. I. Role of T lymphocytes in the clearance of lymphocytic choriomeningitis virus from spleens of mice. J Immunol. 1985;134(1):608–15.

[9] Veiga-Fernandes H, Walter U, Bourgeois C, McLean A, Rocha B. Response of naïve and memory CD8+ T cells to antigen stimulation in vivo. Nat Immunol. 2000;1(1):47–53. doi:10.1038/76907.

[10] Murali-Krishna K, Altman JD, Suresh M, Sourdive DJ, Zajac AJ, Miller JD, et al. Counting antigen-specific CD8 T cells: a reevaluation of bystander activation during viral infection. Immunity. 1998;8(2):177–87. doi:10.1016/S1074-7613(00)80470-7.

[11] De Boer RJ, Oprea M, Antia R, Murali-Krishna K, Ahmed R, Perelson AS. Recruitment times, proliferation, and apoptosis rates during the CD8+ T-cell response to lymphocytic choriomeningitis virus. J Virol. 2001;75(22):10663–10669. doi:10.1128/JVI.75.22.10663-10669.2001.

[12] Chao DL, Davenport MP, Forrest S, Perelson AS. A stochastic model of cytotoxic T cell responses. J Theor Biol. 2004;228(2):227–240. doi:10.1016/j.jtbi.2003.12.011.

[13] Kaech SM, Hemby S, Kersh E, Ahmed R. Molecular and functional profiling of memory CD8 T cell differentiation. Cell. 2002;111(6):837–851. doi:10.1016/s0092-8674(02)01139-x.
